# Bone Adhered Sediments as a Source of Target and Environmental DNA and Proteins

**DOI:** 10.1093/molbev/msaf202

**Published:** 2025-08-26

**Authors:** Toni de-Dios, Biancamaria Bonucci, Rémi Barbieri, Alena Kushniarevich, Eugenia D’Atanasio, Jenna Dittmar, Craig Cessford, Anu Solnik, John E Robb, Christina Warinner, Ester Oras, Christiana L Scheib

**Affiliations:** Institute of Genomics, University of Tartu, Tartu 51010, Estonia; Institute of Genomics, University of Tartu, Tartu 51010, Estonia; Institute of Genomics, University of Tartu, Tartu 51010, Estonia; Institute of Genomics, University of Tartu, Tartu 51010, Estonia; Institute of Molecular Biology and Pathology, National Research Council, Rome 00185, Italy; Department of Anatomical Sciences, Edward Via College of Osteopathic Medicine, Monroe 71203, LA, USA; Cambridge Archaeological Unit, University of Cambridge, Cambridge, UK; Core Lab Facility, Institute of Genomics, University of Tartu, Tartu 51010, Estonia; Department of Archaeology, University of Cambridge, Cambridge CB2 3EJ, UK; Department of Archaeogenetics, Max Planck Institute for Evolutionary Anthropology, Leipzig, Germany; Department of Anthropology, Harvard University, Cambridge, MA, USA; Department of Archaeology, University of Tartu, Tartu 51005, Estonia; Institute of Chemistry, University of Tartu, Tartu 5041, Estonia; Swedish Collegium for Advanced Study (SCAS), Uppsala 752 38, Sweden; Institute of Genomics, University of Tartu, Tartu 51010, Estonia; St John's College, University of Cambridge, Cambridge CB2 1TP, UK; Department of Zoology, University of Cambridge, Cambridge CB2 3EJ, UK

**Keywords:** ancient DNA, metagenomics, paleoproteomics, population genetics

## Abstract

In recent years, sediments from cave environments have provided invaluable insights into ancient hominids, as well as past fauna and flora. Unfortunately, however, sediments are not always collected during excavation. In this study, we analyzed an overlooked but abundant resource in archaeological collections - sediments adhered to bone. We performed metagenomics and metaproteomics analysis on sediment from several human skeletal elements, originating from Neolithic to Medieval sites in England. We were able to reconstruct a partial human genome, the genetic profile of which matches that recovered from the original skeletal element. Additionally, aDNA sequences matching the genomes of endogenous gut microbiome bacteria were identified. We also found the presence of genetic sequences corresponding to animals and plants. In particular, we managed to retrieve the partial genome and proteome of a Black Rat (*Rattus rattus*), sharing close genetic affinities to other medieval *Rattus rattus*. Our results demonstrate that material that is usually ignored or discarded, can be used to reveal information about the individual and the environmental conditions at the time of their death.

## Introduction

The study of sedimentary ancient DNA (sedaDNA) has profoundly broadened our knowledge about deep human genetic history (Slon et al. 2017). This advance is of crucial importance for certain cases, such as identifying the presence of Neanderthals or the Denisovans, where high quality skeletal samples suitable for ancient DNA (aDNA) studies are scarce or practically nonexistent (Zhang et al. 2020; Zavala et al. 2021). The application of sedaDNA techniques has also allowed the successful reconstruction of ancient environments, providing valuable data about past flora and fauna (Pedersen et al. 2016; Massilani et al. 2022; Gelabert et al. 2025). This has been key to understanding changes in vegetation composition over time, spanning different environmental and climatological conditions (Pedersen et al. 2013; Alsos et al. 2016). The use of sedaDNA is not limited to qualitative reconstructions but also allows for phylogenetic and population genetic analysis of ancient fauna (Gelabert et al. 2021; Wang et al. 2021; Kjær et al. 2022). Furthermore, sedaDNA has also been used to retrieve ancient microbial and viral communities (Armbrecht et al. 2022; Pérez et al. 2022; Fernandez-Guerra et al. 2023). Unfortunately, suitable conditions for sedaDNA preservation are mostly restricted to polar latitudes where sediments are frozen in the form of permafrost, caves where temperatures remain constant year-round, or lake sediments (Dabney et al. 2013; Pedersen et al. 2013; Slon et al. 2022). Other types of sediment like materials have been tested, such is the case of stone surrounding skeletons, in which endogenous human aDNA was recovered, in addition to environmental and decomposing bacteria (Sarhan et al. 2021).

Conversely, palaeoproteomics can be applied in a broader range of environmental conditions, including from equatorial, semitropical, high temperature, and temperate deposit conditions (Demarchi et al. 2016; Hendy et al. 2018; Le Meillour et al. 2018; Cappellini et al. 2019; Welker et al. 2019), offering complementary and generally better preservation and abundance of biological materials over time. Although proteins provide less phylogenetic information than aDNA, they offer unique and valuable insights into biological evolution, heritable traits, and functional information of ancient organisms. Palaeoproteomics have significantly enhanced our understanding of ancient hominin diversity, particularly in cases where aDNA has long since degraded. These methods have enabled key discoveries, such as determining the sex and phylogenetic placement of a *Homo antecessor* specimen (Welker et al. 2020), and identifying Denisovans solely through protein analysis (Chen et al. 2019). In contrast, aDNA analysis offers higher-resolution insights into population structure (Olalde et al. 2023; Carrión et al. 2024), dietary habits (Weyrich et al. 2017), and interbreeding events humans and archaic hominins (Reich et al. 2010; Sümer et al. 2025).

Ancient proteins also help to resolve the taxonomic relationships of various animal species, including extinct birds and mammals (Buckley 2019; Horn et al. 2019; Rao et al. 2020). While palaeoproteomics has mainly been applied to analyzing human or animal remains, adhesives and paint binders, or food residues in pottery (Hendy et al. 2018; Bleasdale et al. 2021; Runge et al. 2021), recent advancements have extended its theoretical application to archaeological sediments (Kanerva et al. 2013). To the date, however, metaproteomics of ancient sediments has only been used successfully to identify and isolate silk proteins from historical periods in specific archaeological contexts (Giuffrida et al. 2018; Hendy et al. 2018; Solazzo et al. 2019; Li et al. 2021).

In contrast to aforementioned biomolecules sources, sediments adhered to human skeletal remains are a readily available and abundant material that is often ignored or discarded during excavation, postexcavation cleaning, or laboratory processing (Damgaard et al. 2015; Pinhasi et al. 2019). Bone-adhered sediment is extremely persistent in skeleton collections and can be found after meticulous cleaning or years of storage. In some cases, these sediments could be the last remaining material that provides environmental data of a site years after its excavation. Despite its abundance, the role of bone-adhered sediments (not including dental calculus) as a reliable source of ancient biomolecules is poorly understood. Only a few studies have analyzed their capability of harnessing authentic ancient microbial DNA or protein sequences (Kazarina et al. 2019), and none has investigated its potential for endogenous or environmental biomolecules preservation.

Here, we present a detailed metagenomic and metaproteomic study of bone-adhered sediments from archaeological contexts spanning from the Early Neolithic to the Middle Ages (3762 BCE to 1538 CE). In it, we demonstrate that this novel source of ancient biomolecules can provide valuable information not otherwise directly retrievable from bones. Additionally, although not comparable to skeletal elements, this study demonstrates the value of bone adhered sediments as a nondestructive alternative sampling method for valuable skeletal remains.

## Results

**Fig. 1.**
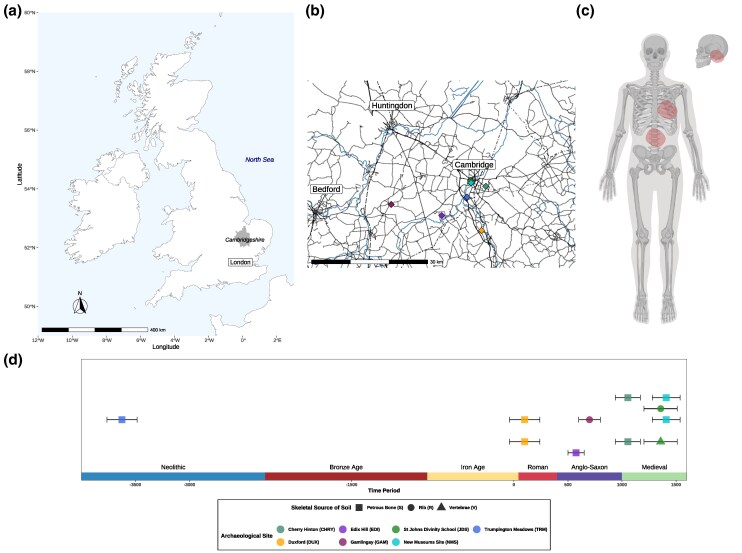
Geographical, chronological, and osteological sampling areas used in this study. a) Map of the United Kingdom with the Cambridgeshire area highlighted. b) Map of the sites used in this study. c) Highlighted with circles, skeletal elements from which the sediments were sampled for this study (Created in BioRender. Bonucci, B. [2025] https://BioRender.com/xok2pd2). d) Timeline of the chronological order of the sites used in this study (legend below). Skeletal elements designated by shape.

Focusing on 11 individuals from 7 sites in Cambridgeshire, England (Fig. 1a, b) (supplementary information SI1 and figs. S1-S13, Supplementary Material online), we have retrieved a total of 11 samples of bone-adhered sediment originating from temporal bone, rib, and vertebrae (Fig. 1c and  Table 1), which after DNA extraction, custom NEBNext® library preparation (see Methods), and shotgun sequencing (dsDNA libraries using Illumina NextSeq500/550), each yielded between 15 and 32 million DNA sequences (supplementary table S1 and figure S14a, Supplementary Material online). An initial metagenomic screening of the samples using Kraken2 (Wood et al. 2019), with the NCBI *nt* database (see Methods section *Metagenomic screening*) classified between 0.98% and 5.2% of the DNA sequences (supplementary figure S14b, c, Supplementary Material online). Such a limited range of assignable sequences is expected due to incompleteness of microbial and eukaryotic genomic databases (Chorlton 2024).

**Table 1:** Provenance and summary information for samples used in this study

Lab ID	Individual ID	Site name	Culture of the site	Sample age	Context	Skeletal element	Mass (mg)
JDS123B	223	St John's Divinity School	Medieval	1049 to 1390 CE (site estimate)	Urban	Vertebrae	6.94
JDS157B	403	St John's Divinity School	Medieval	1049 to 1390 CE (site estimate)	Urban	Rib	3.43
DUX010B	458	Duxford	Iron Age/Roman	29 to 207 calCE	Rural	Petrous	10.04
DUX012B	460	Duxford	Iron Age/Roman	30 to 207 calCE	Rural	Petrous	13.75
NMS031B	509	New Museums Site	Late Mediaeval	1290 to 1538 CE (site estimate)	Urban	Petrous	10.76
NMS022B	518	New Museums Site	Late Mediaeval	1290 to 1538 CE (site estimate)	Urban	Petrous	10.7
EDI013B	559	Barrington Edix Hill A	Early Mediaeval/Anglosaxon	439 to 650 CE (site estimate)	Rural	Petrous	8.63
TRM003B	616	Trumpington Meadows	Neolithic	3762 to 3648 calBCE	Rural	Petrous	15.58
GAM042B	842	Gamlingay	Early Mediaeval/Anglosaxon	500 to 100 CE (site estimate)	Rural	Rib	6.74
CHRY038B	947	Cherry Hinton	Medieval	940 to 1170 CE (site estimate)	Rural	Petrous	14.68
CHRY051B	1155	Cherry Hinton	Medieval	940 to 1170 CE (site estimate)	Rural	Petrous	14.05

Individual ID is the project specific number from the After the Plague Project. Mass is the mass of the sediments used in the extraction. Some radiocarbon dates for the skeletal elements used have previously been published (Scheib et al. 2019; Hui et al. 2024; Scheib et al. 2024). For more information see supplementary material SI1, Supplementary Material online: Archaeological sites.

Proteins were also extracted from the sediment samples using a single-pot, solid-phase protocol modified from (Cleland 2018; Wilkin et al. 2021). Raw data was analyzed using pFind (Wang et al. 2007; Chi et al. 2018), with searches conducted against SwissProt and cRAP databases (Boutet et al. 2007), and only considering peptides with a score below or equal to 0.01 and at least two peptide-spectrum matches (PSMs). For protein hits, they were considered if they presented at least 2 peptides matching a protein (see Methods section for peptide identification and validation criteria). A total of 5,709 peptides were recovered across all samples from 815 different proteins. According to the top hits obtained from pFind, peptides originating from human, bacteria, nonhuman vertebrates, plants and viruses were identified (supplementary fig. S15 and S16, and table S2, Supplementary Material online). We also used *novor*.*cloud* to have additional certainty for protein validation (Ma 2015; supplementary materials SM3, Supplementary Material online).

### Endogenous Human aDNA and Proteins

**Fig. 2.**
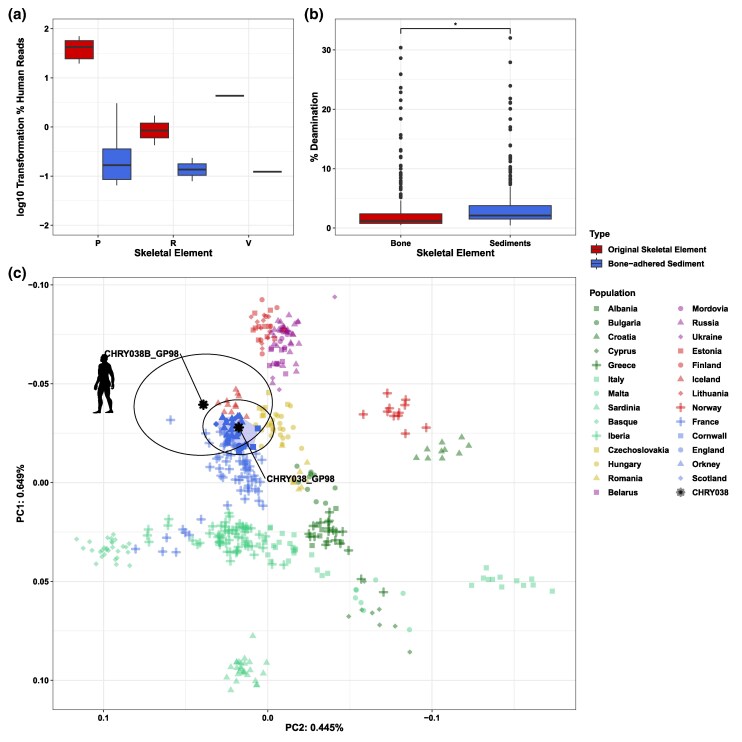
Human DNA content. a) Log10 transformation of the percentage of endogenous human aDNA from original skeletal element versus sediments recovered from that element. b) Deamination proportion at the 30 last positions in both reads' ends from the original skeletal element versus sediments recovered from that element. Significance was assessed using a Welch's *t*-test, with significance annotated using * (*P* ≤ 0.05). c) Principal component analysis of the human aDNA from the sediment sample of CHRY038B and the tooth sample of CHRY038. The PCA was created using smartpca, with 95 CI displayed by black circle using ellconf: 0.95, and projecting our ancient samples against 442 modern samples from Europe. Modern British samples are highlighted in the polygon within the small circle. CHRY038 falls within the 95 CI of the sediment sample, CHRY038B.

Human aDNA sequences were recovered from all samples with the exception of samples from the Duxford site (supplementary table S3, Supplementary Material online). The abundance of endogenous human aDNA does not seem to relate to the endogenous abundance found in the original bone (Hui et al. 2024; Scheib et al. 2024) (Fig. 2a), but the terminal C to T deamination is higher in sediment samples (Welch's *t*-test *P*-value = 0.01547) (Fig. 2b). Genetic sex characterization of the human sequences retrieved from the sediment mostly matches the characterization performed using the original skeletal element sequences (supplementary fig. S17, Supplementary Material online). It is important to remark that for some cases the endogenous content is low, and estimates may not be accurate. A special case is sample CHRY038B (Petrous bone sediment), which yielded a human genome at an average depth of roughly 0.01× (3% of endogenous DNA), making it a potential candidate for population genetic analysis. Genetic sex and mitochondrial haplotype match those of the original element (XX, U5b3) (Hui et al. 2024) (supplementary table S9B, Supplementary Material online), with 0% of contamination based on heterogeneity at sites at the mitochondrial DNA. The pseudo-haploid-called nuclear genome overlapped 8,580 SNPs from the Human Origins (HO) dataset (Lazaridis et al. 2016). We also imputed the genome using QUILT (Davies et al. 2021) and the Haplotype Reference Consortium (HRC) reference panel (McCarthy et al. 2016), and projected it against a panel of modern European populations (Lazaridis et al. 2016). After filtering positions with a minor allele frequency (MAF) under 5% in the HRC reference panel and keeping SNPs with a genotype probability (GP) of 98%, we retrieve a total of 24,469 imputed SNPs for CHRY038B (224,460 SNPs in CHRY038, the original skeletal element). We used READv2 (Alaçamlı et al. 2024) to compare the original bone sample and sediments library, resulting in “IDENTICAL,” both with and without imputation (*P*0 = 0.0665; *P*0 = 0.1535) (supplementary fig. S18 and table S4-S5, Supplementary Material online). The imputed sample falls close in the PCA to the skeleton library, among the samples from Western Europe (95% confidence interval for sediment and bone) overlapping (Fig. 2c; supplementary fig. S19, Supplementary Material online). We also pulled imputed phenotypes to check the concordance between information in the original bone sample and bone-adhered sediments (supplementary table S6, Supplementary Material online). Applying a genotype dosage (DS) of 0.1, we observed a concordance of ∼73.4% with 29 genotypes of the total 109 phenotypic-informative markers differing between the two sample sources (supplementary table S6, Supplementary Material online).

Peptides were assigned to *Homo sapiens* by pFind in each sample, with an average of 22% of the total identified peptides per sample originating from human proteins. A more detailed analysis using Unipept, combined with manual BLASTp verification, revealed that peptides with 100% sequence coverage and specificity only to *Homo sapiens* were further detected in five samples: JDS123B, JDS157B, TRM003B, GAM042B, and GAM042. These peptides correspond to the proteins alpha-2-HS-glycoprotein, Serpin family F member 1, and Hornerin (supplementary table S2, Supplementary Material online). In contrast, the majority of peptides initially assigned to *Homo sapiens* by pFind—mainly collagen—were ultimately attributed to the Hominidae family, lacking species-level specificity.

### Microbial Analysis

**Fig. 3.**
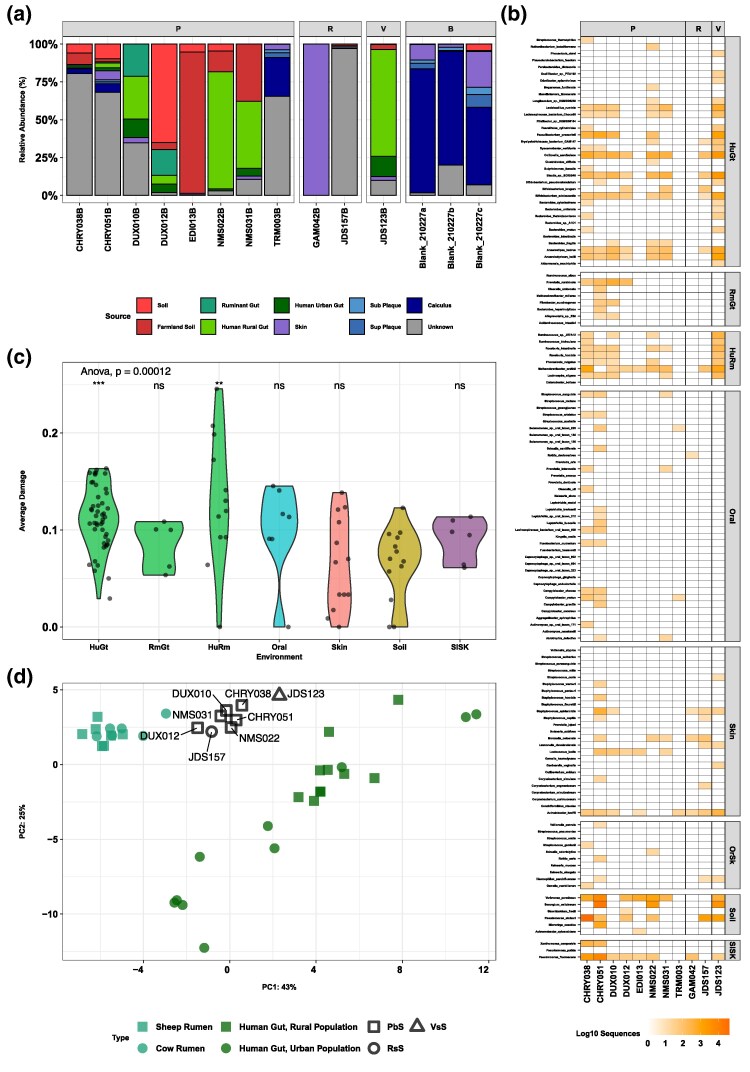
Bacterial composition of the samples in this study. a) Stacked bar chart of MST results estimated by SourceTracker2. b) Presence of source characteristic bacteria per sample inferred by mapping. c) Average terminal damage by each source. Source means were compared using an ANOVA test. Statistical significance is marked using * (*P* ≤ 0.05), ** (*P* ≤ 0.01), and *** (*P* ≤ 0.001). d) Principal component analysis of gut-associated bacteria in the analyzed samples and published gut microbiome data from humans and domesticated ruminants (cow and sheep).

After reanalyzing the metagenomic data using KrakenUniq (Breitwieser et al. 2018; Pockrandt et al. 2022), we generated a microbial assignment dataset based on hits with an E-score above 7 (see Methods). The microbial source tracking (MST) analysis of the samples using SourceTracker2 (McGhee et al. 2020) revealed heterogeneous source attributions, with no clear pattern consistent with anatomical sampling location (Fig. 3a; supplementary table S7A, Supplementary Material online).

NMS022B and NMS031B (Petrous bone—PbS), and JDS123B (Vertebrae—VsS) display a high abundance of microbial species originating from the human gut microbiome (78.7%, 49.4%, and 84%, respectively). DUX10B and DUX12B (PbS) also exhibit a moderate proportion of human gut microbiota (40.5% and 11.5%), as well as species associated with the rumen of domestic animals (21.3% and 17%). CHRY038B, CHRY051B, and TRM003B (PbS) present mostly traces of human oral bacteria (3.2%, 8.2%, and 30.8%), with the remainder of the community being unassignable. Finally, EDI013B (PbS), GAM042B, and JDS157B (Rib–RsS) seem to be composed in their totality of environmental or contaminant sources: farmland soil, skin, and an unknown source, respectively (supplementary table S7B–D, Supplementary Material online). A nonmetric multidimensional scaling (nMDS) from the same dataset using Bray–Curtis distances reflects similar tendencies, with most of the samples falling in between the diversity of the reference sources, with the exception of TRM003B and GAM042B, which fall close to the blanks (supplementary fig. S20, Supplementary Material online). We excluded potential contaminant taxa as found in extraction blanks using decontam with a threshold of 0.7 (Davis et al. 2018). Those included 121 microbial species (supplementary table S7E and fig. S21, Supplementary Material online).

A closer inspection of the KrakenUniq screened data using source-specific species assigned by SourceTracker2 *per_sink_feature_assignments* mode (see supplementary methods S1, Supplementary Material online) reveals patterns not obvious using SourceTracker2 default mode alone (Fig. 3b). Most of the PbS samples (CHRY, DUX, and NMS) contain traces of human gut and/or ruminant gut microbial species. Finally, we have detected and validated the presence of the pathogenic bacterium *Mycobacterium leprae* sequences in CHRY051B (supplementary fig. S22 and table S8, Supplementary Material online).

Mapping of the sequences assigned to the source-characteristic species and subsequent aDNA damage pattern characterization reveals the presence of deamination levels consistent with those expected for authentic ancient DNA sequences (Fig. 3c; supplementary fig. S23, Supplementary Material online). The damage levels vary depending on the inferred source, but not the skeletal element from which the sediments were sampled (supplementary table S9, Supplementary Material online), being significantly higher in species from the human gut microbiomes than from environmental species (*P*-value < 0.05 compared to Soil).

Given the abundance of gut-associated microbial sequences in the samples, we decided to further explore the composition by performing a Principal Component Analysis (PCA). This included 79 microbial species found across 33 of the included reference metagenomes (10 Human Urban Gut, 10 Human Rural Gut and 13 Ruminants' Gut) (Rampelli et al. 2015; Stewart et al. 2019; Tisza and Buck 2021; Su et al. 2022) and eight of our sediment samples (CHRY, DUX, NMS, and JDS) (Fig. 3d). The reference samples for human urban gut, human rural gut and ruminant gut form three distinct clusters within the PCA (Fig. 3d; supplementary fig. S24, Supplementary Material online). All sediments samples fall within the diversity described by the PCA, forming a cline between samples with higher levels of inferred rumen microbes falling closer to the ruminant gut and samples more closely the gut microbial taxa typical of nonindustrialized populations. This indicates a certain degree of metagenomic overlap between microbial species abundances originating from humans and domestic animals.

In regard to potential bacterial proteins, the DUX012B sample has a high proportion of identified bacterial peptides (38%) compared to the other samples (supplementary figs. S15 and S16, Supplementary Material online). However, none of the bacterial peptides identified in the samples could be exclusively linked to a pathogen, or to microbes associated with human microbiota.

### Nonhuman Eukaryotes Metagenomics and Metaproteomics

The metagenomic data were screened for the presence of eukaryotic species using a custom pipeline (see methods, supplementary methods S2, figs. S25-S26, and table S10A, B, Supplementary Material online). We removed all reads assigned to microbial species and possible contaminants as identified by KrakenUniq (Breitwieser et al. 2018). The remaining sequences were rescreened using a database of plastid and mitochondrial DNA from RefSeq (O’Leary et al. 2016; supplementary figs. S27-S28, Supplementary Material online). Sequences were validated according to their E-score, with the goal of finding a suitable reference genome. We selected species with more than three mitochondrial hits per genus and with an E-score above 7. Consequently, we performed a set of competitive mappings between the selected reference genomes to determine the best match. Additionally, we calculated edit distance distribution, terminal damage patterns, read length distribution, and read coverage of the mapped sequences for each sample and species. Organisms such as “Plants” or “Algae” were only classified down to order level due to the high level of homology between retrieved sequences. Despite lower resolution, analysis of terminal damage reveals damage levels compatible with authentic aDNA data (supplementary fig. S29, Supplementary Material online).

**Fig. 4.**
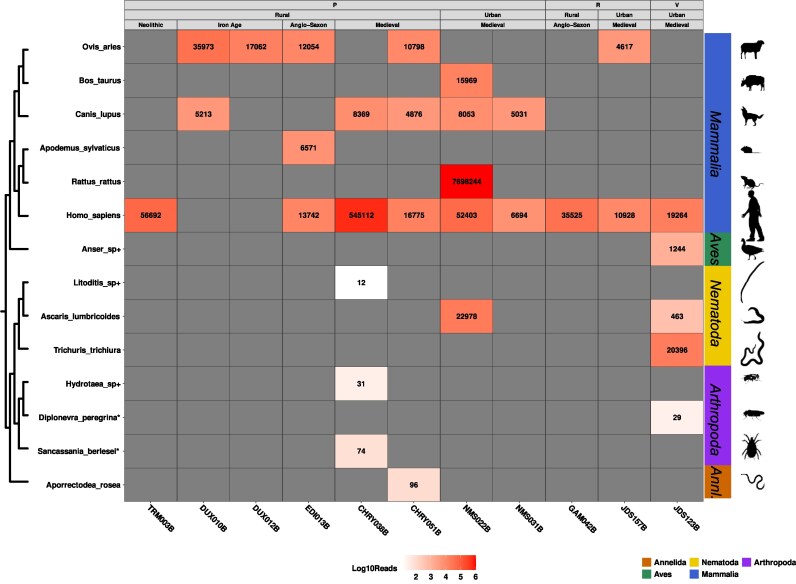
Number of reads from a select panel of animal species present in the samples. Read assignments were determined using a custom screening pipeline described in this study (see methods). Vertical axis is ordered by the taxonomic location of detected species, with some species marked by * (Only mitochondrial reference available) and + (Taxonomic level could not be determined below genus level). Horizontal axis is the sample. Samples are grouped by their sampling origin (P = Petrous Bone sediments, R = Rib sediments, V = Vertebrae sediments), context of site (Rural vs. Urban), and site dating (Neolithic, Iron Age, Anglo-Saxon, and Medieval). Read numbers are in log10 scale with higher numbers displayed with a darker cell filling. The bar on the right denotes the taxonomic group in the following order: *Mammalia*, *Aves*, *Nematoda*, *Arthropoda*, and *Annelida*).

After whole genome mapping, we recovered sequences belonging to *Homo* (1,744 to 545,112), *Ovis* (35,913 to 4,617), *Bos* (15,969), *Canis* (8,369 to 4,876), *Trichuris* (20,396), *Ascaris* (22,978 to 463), *Apodemus* (6,571), and *Rattus* (7,698,244) genera (Fig. 4 and Table 2; supplementary table S10C, Supplementary Material online). We also found and reported low abundance species (supplementary fig. S30, Supplementary Material online). Most of the sequences display features such as length, terminal deamination, and edit distance distribution compatible with ancient genomes (supplementary figs. S31-S33, Supplementary Material online). Putative contaminant data found in extraction blanks (*Canis*, *Homo*, and *Meleagris)* did not display aDNA associated damage (supplementary table S10D, Supplementary Material online), validating the authenticity of the sequences assigned to *Canis* and *Homo* in the sediment data. Coincidentally, two of the samples presenting lower damage *Ovis arie*s aDNA sequences (DUX010B and DUX012B) were also showing traces of recent ruminant gut associated microbes DNA in the MST. We discarded the presence of human sequences in those same samples due to the low number of sequences left over after reclassification, high mean edit distance (indicative erroneous mapping), and relatively low terminal deamination. In the vertebral sample (JDS123B), we have found sequences assigned to parasitic species (*Ascaris lumbricoides*, roundworm; *Trichuris* spp., whipworm). Unfortunately, we could not discern the anatomical origin of vertebral remains due to the conservation state of the bone. Finally, we also find DNA from possible scavengers (*Diptonevra peregrina*, fly; *Hydrotaea* spp., fly; *Sancassania berlesi*, mite; and *Apodemus sylvaticus*, wood/field mouse).

**Table 2:** Mapping statistics of different animals retrieved

Individual	Specie	Mapped reads	Av. damage last 3 bp	Av. edit distance	Average coverage
CHRY038B	*Homo sapiens*	545,112	0.138	0.62	0.0099×
CHRY038B	*Canis lupus*	8,369	0.119	1.348	0.0002×
CHRY051B	*Homo sapiens*	16,775	0.109	0.608	0.0004×
CHRY051B	*Canis lupus*	4,876	0.145	1.41	0.0001×
CHRY051B	*Ovis aries*	10,798	0.143	1.39	0.0003×
DUX010B	*Canis lupus*	5,213	0.075	1.214	0.0001×
DUX010B	*Ovis aries*	35,973	0.086	0.89	0.0007×
DUX012B	*Ovis aries*	17,062	0.067	0.918	0.0003×
EDI013B	*Apodemus sylvaticus*	6,571	0.112	1.1472	0.0001×
EDI013B	*Homo sapiens*	13,742	0.186	0.962	0.0002×
EDI013B	*Ovis aries*	12,054	0.167	1.275	0.0002×
GAM042B	*Homo sapiens*	35,525	0.134	0.829	0.0005×
JDS123B	*Anser* spp.	1,244	0.097	2.174	0×
JDS123B	*Trichuris trichiura*	20,386	0.128	1.433	0.0152×
JDS123B	*Ascaris lumbricoides*	463	0.118	1.687	0.0001×
JDS123B	*Homo sapiens*	19,264	0.159	0.878	0.0003×
JDS157B	*Homo sapiens*	10,928	0.072	0.906	0.0002×
JDS157B	*Ovis aries*	4,617	0.086	1.631	0.0001×
NMS022B	*Rattus rattus*	7,698,226	0.065	0.551	0.1842×
NMS022B	*Ascaris lumbricoides*	22,978	0.142	1.149	0.0042×
NMS022B	*Homo sapiens*	52,403	0.141	0.92	0.0009×
NMS022B	*Canis lupus*	8,053	0.126	1.328	0.0002×
NMS022B	*Bos taurus*	15,969	0.108	1.079	0.0003×
NMS031B	*Homo sapiens*	6,694	0.142	0.944	0.0001×
NMS031B	*Canis lupus*	5,031	0.133	1.3267	0.0001×
TRM003B	*Homo sapiens*	56,692	0.22	0.847	0.0009×

Mapping statistics of the identified animals in the samples, those include number of mapped sequences, average edit distance, average deamination in the last 3 bp of each read and average depth of coverage. Only Animals with whole genome data have been included.

**Fig. 5.**
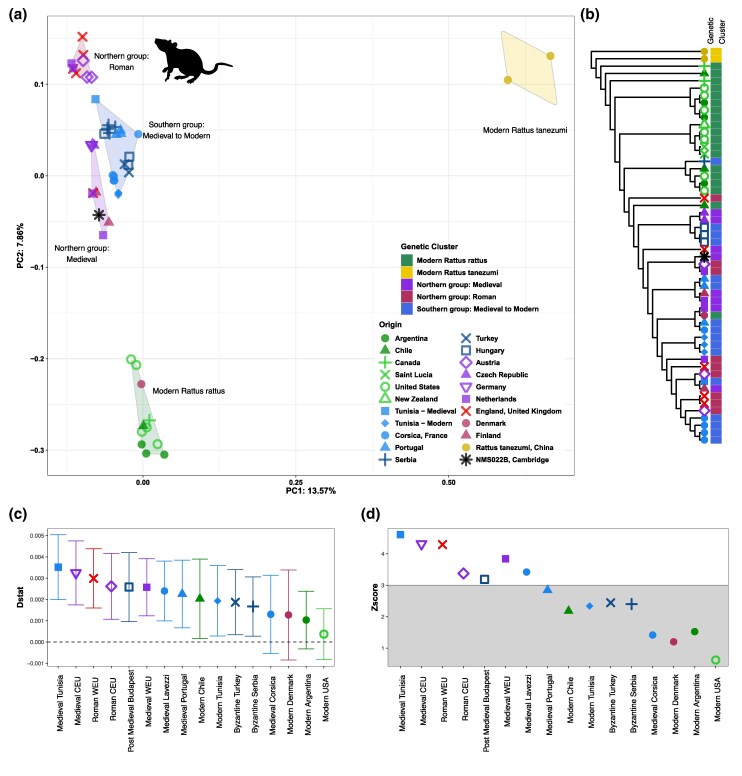
Population genomic analyses of the ancient *R. rattus* genome recovered from NMS022B. a) PCA created by PCAngsd using 513,655 variable positions found in NMS022B and 51 modern and ancient *R*. *rattus*. Cluster names are annotated in the figure. PC1 and PC2 account for 13.57% and 7.86% of the described variance respectively. b) ML Tree created using RAxML, a dataset of 3,467 SNPs found across 53 modern and ancient *R*. *rattus* and NMS022B. Clusters are annotated in color-coded format. c) f4-statistics (Dstat) under the test relationship f4 (*R. tanemuzi*, NMS022B, Modern_Canada, X) of NMS022B sample and other historical and modern *R*. *rattus* populations. d) Z-scores under the test relationship assessed through block jack-knife resampling.

Perhaps most interesting, within NMS022B, we found a large number of sequences assigned to the black rat (*Rattus rattus*), with an average depth of coverage of 0.1877× for the nuclear and 8.09× for the mitochondrial genomes. By analyzing the heterozygosity level of the mitochondrial genome (0 after filtering for potential aDNA damage by removing transitions), we conclude that the sequences most likely originate from a single male individual, given the ratio of X and Y chromosome sequences (1/1, 1 to 2 autosomes) (supplementary fig. S34, Supplementary Material online). We created a genome-wide SNP dataset of historical (Yu et al. 2022) and modern (Puckett et al. 2016) *R*. *rattus* and then performed a PCA and mitochondrial ML tree, including our Medieval genome (Fig. 5a, b; supplementary fig. S35, Supplementary Material online). The NMS022B rat genome displays affinities to other Western and Northern European Medieval and Early Modern rats. We formally validated this result conducting an *f*4 analysis (D statistics) in AdmixTools (Patterson et al. 2012) concluding NMS022B is genetically closer to other medieval black rats (Fig. 5c; supplementary fig. S36, Supplementary Material online).

**Fig. 6.**
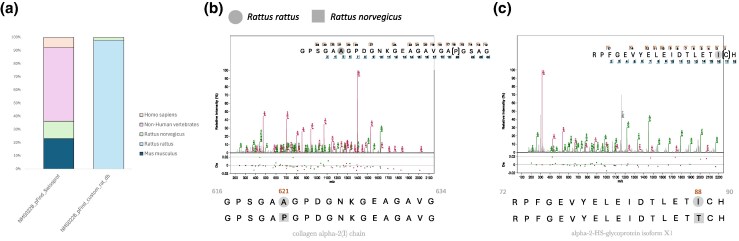
*R. rattus* specific peptide identification from sample NMS022B. a) Stacked bar plot showed the relative abundance of peptide origins based on SwissProt identification from pFind output and our custom *R*. *rattus* database. b) Spectrum from GPSGAAGPDGNKGEAGAVGAPGSAG (PSM count = 26) (collagen alpha-2 [I] chain) identified with pFind specific to *R*. *rattus*.Amino acids shown in parentheses indicate hydroxylated proline residues. Sequence variation at position 621 of the protein (highlighted by a circle for *R. rattus* and by a square for *R. norvegicus*) was used to distinguish between the two species. c) Spectrum of the peptide RPFGEVYELEIDTLETICH (PSM count = 2) (alpha-2-HS-glycoprotein isoform X1) identified with pFind as specific to *R*. *rattus*. Amino acids shown in parentheses represent carbamidomethylation of cysteine residue. Sequence variation at position 88 of the peptide (highlighted by a circle for *R. rattus* and a square for *R. norvegicus*) was used to distinguish between the two species.

The palaeoproteomic data also supports the presence of *R*. *rattus* in the sample. NMS022B contains the highest number of total unique peptides (1,141) and proteins (125) across all samples. Furthermore, this sample also contains the highest number of peptides assigned to Rodents; *R*. *norvegicus* (148) or *M*. *musculus* (269). Because the SwissProt database contains protein sequences for *R*. *norvegicus* but not *R*. *rattus*, we created a database incorporating all published protein sequences from both species on NCBI (supplementary file S1, Supplementary Material online). Analyzing the data using this database, we identified a total of 2,758 peptides; with 2,719 assigned to *R*. *rattus*, and 39 to *R*. *norvegicus* (Fig. 6a). This supports *R*. *rattus* as the most probable source of *Rattus genus* peptides found in NMS022B.

We next focused on analyzing specific peptides to distinguish between these two species. In order to confirm that the peptides come from *R*. *rattus*, we focused on the GPSGAAGPDGNKGEAGAVGAPGSAG (PSMs count = 26) peptide sequence from collagen alpha-2 (1) chain, and the RPFGEVYELEIDTLETICH (PSMs count = 2) peptide sequence from the alpha-2-HS-glycoprotein isoform XI protein. This scrutiny pinpoints differences at two amino acid positions that allow the differentiation of *R. rattus* from *Rattus norvegicu*s (Fig. 6b, c). Overall, we successfully identified 44 partial proteins (≥5% of coverage) belonging to *R. rattus* (supplementary table S12, Supplementary Material online).

## Discussion

Our results show that authentic traces of ancient biomolecules from the host, its microbiome (gut and oral), and the environment can be retrieved from bone-adhered sediments sampled from archaeological remains. Even though the bones used in this study were cleaned at the time of excavation, years before sampling, and were stored in various controlled and noncontrolled environments, residual sediments were still present and provided valuable biomolecular information. Human aDNA preservation in bone-adhered sediments varies greatly between samples and sample type. It would be necessary to emphasize that this kind of samples are metagenomic in nature (Slon et al. 2017), and that genomic material from several individuals could be contained in the data, rendering downstream analysis difficult to interpret or even infeasible. Therefore, modern contamination estimates, followed by estimation of the presence of one or more individuals should be performed to avoid biases or erroneous results. However, a previous study with stones under skeletal remains (Sarhan et al. 2021), and the example provided with the bone attached sediments from CHRY038B in this study, seem to point that this type of materials surrounding human remains tend to contain mostly endogenous sequences. In scenarios like this, enough coverage can be obtained to reveal valuable information including ancestry, genetic sex, and even phenotypes. Furthermore, other individuals show enough endogenous DNA content to make them suitable candidates for target capture (Sarhan et al. 2021; Villalba-Mouco et al. 2023).

Ancient gut bacteria have been previously recovered from paleofeces (Runge et al. 2021; Wibowo et al. 2021), but these are rare finds. The potential to recover ancient gut bacteria from bone-adhered sediments opens up new opportunities in microbiome studies. Although putatively ancient, the gut bacteria we recover seem to be a mixture of both human and domestic animal gut microbiota, suggesting a high degree of environmental noise. Even if only considering human-associated gut sequences, we cannot discard contemporaneous contamination due the lack of sanitation measures at the time. Despite postmortem community alteration that could make sediments unsuitable for microbiome and community compositional changes analysis, sediments could still be helpful for the recovery of individual or target microbial species (Wibowo et al. 2021). In contrast, the simplest explanation for the presence of oral signal in the samples seems to be an endogenous source, leaching from the oral cavity to the inner ear and petrous bone area during the decomposition process (Payen et al. 1988), or by direct decomposition activity of the body by the individual's own endogenous oral microbes (Mann et al. 2018). Contamination during the excavation or laboratory procedures cannot also be ruled out. Apart from typical human commensals, we found *M*. *leprae* in individual CHRY051 (SK3463), and although we did not find genetic or palaeopathological evidence of infection in this particular individual, other individuals in the site were tested positive (Pfrengle et al. 2021). The presence of *M*. *leprae* sequences in the surrounding sediments could be evidence of DNA leaching during the decomposition process of the body. It is important to remark that the grave of this particular individual was cut through an earlier grave (SK3505), so mixture with earlier sediments is possible (Ferrante di Ruffano and Waldron 2006). Another possible explanation is that it represents an early infectious stage of the disease, infecting only soft tissues.

Despite most of the samples originating from sediments attached to the petrous bone, which is a priori relatively isolated from the environmental sediments, there is a high abundance of DNA from environmental sources. This is the case for plant aDNA, mainly assigned to shrub or grass phyla, and that we can find in most of the samples. Interestingly, the sample with the highest number of plant aDNA sequences (TRM003B) also presented four plant virus peptides. Animal sequences are also widespread across all the sample types and sites. Those include common domestic animals such as dog, cow, sheep, and a goose species; or scavengers such as the wood mouse or different fly species. This is no surprise given the abundance of animal remains at the archaeological sites (Malim 1998; Cessford and Dickens 2006; Murray and McDonald 2006; Patten 2012; Cessford 2017). In particular, in the site of Duxford, an abundant number of animal bones, associated with human consumption, were found inside and close to the burials (Lyons 2011). Those could have left DNA traces due to proximity to the deposition site or by actively interacting with the body. Further evidence supporting this is that those samples contain more *Ovis* aDNA and also have a higher content of ruminant microbial sequences. We also found potential traces of human intestinal parasites (pinworms and whipworms), and if this is a target for research, then bone-adhered sediments from the pelvic region, i.e., pelvis or vertebrae, could be a good source of parastie aDNA for such studies. Pelvic sediment is already being used as a source for parasite studies (Anastasiou et al. 2018; Gildner and Casana 2021), but if this is not available because it was not collected during past excavations, bone-adhered sediments from stored remains could still be a valuable source.

The palaeoproteomics results somewhat mirror those of the aDNA, providing further evidence for the authenticity of the ancient proteins. In addition to *Homo sapiens* specific peptides, our samples exhibit specific peptides from other mammals, such as *Oryctolagus cuniculus*, *Gallus gallus*, *Mus sp*., *Equus sp*., or from fish such as *Epinephelus* sp. Furthermore, plant, bacteria, environmental protista, and plant virus were also detected in three samples (TRM003B, DUX012B, and GAM042B). One sample (TRM003B) yielded a high abundance of aDNA sequences and peptides belonging to the *Poaceae* and *Brassicaceae* families. Unfortunately, these are not specific, so the species cannot be confirmed with proteins alone. Palaeoproteomics results confirm the feasibility of isolating and identifying ancient proteins from sediment samples using metaproteomic techniques. This allowed us to analyze for the first time sediment samples using both proteomics and aDNA techniques, generating consistent results between the two analyzed ancient biomolecules.

In the urban context, the finding of a partial genome of a black rat supported with palaeoproteomics in a site containing plague victims (Spyrou et al. 2019; Cessford et al. 2021) is particularly remarkable due to its role as a vector in spreading *Y*. *pestis* during the Black Death. Although *Y*. *pestis* has been found in ancient rat remains (Morozova et al. 2020), no trace was found in these sediments. It is possible that this rat was not a carrier or infected with the bacterium. How the ancient rat genome and proteome came to be incorporated in the sediments inside the petrous bone of this individual is unclear. Analysis of protein origin did not provide any further information, as proteins were evenly distributed between blood, collagen, bone, cartilage or other origin tissues (supplementary table S12, Supplementary Material online), and not associated with any particular tissue or body fluid that might shed light on the rat's aDNA and ancient proteins introduction in sediments. The genome of the rat is clearly ancient, but whether it is contemporary with the burial is uncertain. Rat bones were not reported near the body in the original excavation; however, due to their small size they are often missed and difficult to recover. Nevertheless, given the care taken excavating human skeletons, they would probably have been identified if present, and there was no evidence of gnawing on the bones (Cessford and Neil 2022; Cessford et al. 2022 , 2023). The black rat went extinct in most of eastern England by the 18th century, following competition from the brown rat (Barrett-Hamilton 1910; Yu et al. 2022). A possible explanation for the presence of rat aDNA could be from feces or urine deposited on or near the body before or during burial. The sediments in the grave fill appear to be the material that was excavated to create the grave. The site had been occupied for several centuries prior to becoming a cemetery, being possible that the rat aDNA predates the burial and relates to the earlier occupation. These findings have been corroborated by palaeoproteomics, validated by two independent tools: *novor*.*cloud* and pFind. To our knowledge, this represents the first reported ancient draft proteome of *R*. *rattus*, encompassing 42 partially covered proteins. This could constitute a remarkable and rare correspondence between aDNA and ancient proteins.

## Conclusion

Bone-adhered sediments are a unique and valuable resource for obtaining ancient genomic and proteomic data in a nondestructive manner. This study highlights the potential of bone-adhered sediments for studying the necrobiome, recovering ancient biomolecules of ancient bodies in a nondestructive manner. This includes sufficient human endogenous aDNA matching the original skeleton to perform genetic ancestry analysis; environment aDNA and peptides that can provide a glimpse at the conditions in which the body was deposited, and ancient microbial data. Although endogenous human biomolecules retrieved using this method are not as abundant as those recovered from traditional skeletal sampling, bone-adhered sediment can be used in a complementary fashion, providing other types of supporting information for the researcher.

## Methods

All of the laboratory work was performed in dedicated ancient DNA facilities of the Institute of Genomics, University of Tartu. The library quantification and sequencing were performed at the Estonian Biocenter Core Laboratory. The main steps of the laboratory work are detailed below.

### Selection of Archaeological Samples

Between 3.43 and 15.58 mg of sediments from the ribs surface (samples GAM042B, JDS157B), vertebra (JDS123B) and petrous bones (all other samples) was removed from the bone using a dental scraper cleaned between uses with 6% v/w NaOCl and rinsed with double distilled water and 70% ethanol. Sediments were collected into DNA lo-bind 1.5 ml tubes (Eppendorf).

### Extraction of Ancient DNA and Protein Data

To each sediment sample, 500 µl of 0.5 M EDTA pH 8.0 was added, and samples were rocked at room temperature overnight. From the extracts, 450 µl of supernatant were removed and purified according to manufacturer's instructions using buffers from the Minelute^TM^ PCR Purification Kit (Qiagen) with changes as described in the online protocol (Keller and Scheib 2023) and the following project specific update: samples were eluted in 60 µl of EB buffer instead of 100 µl. One extraction was performed per sample for screening and 30 μl used for libraries.

Library preparation was conducted using a protocol modified from the manufacturer's instructions included in the NEBNext® Library Preparation Kit for 454 (E6070S, New England Biolabs, Ipswich, MA) as detailed in Keller et al. (2023), except that libraries were single indexed and not split into two amplifications for PCR. DNA was amplified using the following PCR set up: 50 μl DNA library, 1 × PCR buffer, 2.5 mM MgCl_2_, 1 mg/ml BSA, 0.2 μM inPE1.0, 0.2 mM dNTP each, 0.1 U/μl HGS Taq Diamond and 0.2 μM indexing primer. Cycling conditions were: 5′ at 94 °C, followed by 18 cycles of 30 s each at 94 °C, 60 °C, and 68 °C, with a final extension of 7 min at 72 °C. Amplified products were purified using MinElute columns and eluted in 35 μl EB (Qiagen). Three verification steps were implemented to make sure library preparation was successful and to measure the concentration of dsDNA/sequencing libraries—fluorometric quantitation (Qubit, Thermo Fisher Scientific), parallel capillary electrophoresis (Tapestation, Agilent) and qPCR. More information about the laboratory workflow can be found at supplementary table S13, Supplementary Material online. Samples were shotgun-sequenced to a depth of ∼20 million reads on the Illumina NextSeq500/550 using the High-Output single-end 75 base pair kit at the University of Tartu Institute of Genomics Core Facility. The ancient DNA data generation from the original skeletal elements was done as described in the relevant publications (Scheib et al. 2019; Hui et al. 2024; Scheib et al. 2024).

The pellet and remaining 50 µl supernatant were used for protein isolation using a modified version of the SP3 protocol used in Bleasdale et al. (2021) and available on protocols.io (Wilkin et al. 2021). To the pellets, 150 µl of 2 M guanidine HCl was added, then 20 µl each of 100 mM CAA and TCEP. Samples were incubated at 99C for 10 min then allowed to cool. To the sample, 10 µl of a mix of Sera-Mag Carboxylate SpeedBeads (hydrophobic Cat. #45152105050250 and hydrophilic Cat. #65152105050250) was added along with 230 µl of 100% Ethanol and incubated at 24 °C for 5 min at 500 rpm. Beads were pelleted using a magnetic rack and the supernatant removed. Beads were washed three times with 80% ethanol then 150 µl of TEAB and 1 µl trypsin were added to each tube. Samples were incubated overnight at 37 °C at 500 rpm. In the morning, Pierce™ C18 Tips, 100 µl beds were used to immobilize the peptides. Tips were prepared with methanol, AT80 and 0.1% TFA. Samples were run through the tips twice and followed by two rounds of 0.1% TFA. Tips were stored at −20 °C until delivered to the Proteomics Core Facility at the University of Tartu.

Samples were eluted from C18 StageTips and reconstituted in 21 μl of 0.5% TFA. LC-MS/MS analysis was carried out by loading the entire sample to a 0.3 × 5 mm trap-column (5 µm C18 particles, Dionex) using an Ultimate 3500 RSLCnano system (Dionex, California, USA). Peptides were eluted to an in-house packed (3 µm C18 particles, Dr Maisch, Ammerbuch, Germany) analytical 50 cm × 75 µm emitter-column (New Objective, Massachusetts, USA) and separated at 250 nl/min with an A to B 5% to 35% 1.5 h gradient (buffer A: 0.1% formic acid, buffer B: 80% acetonitrile + 0.1% formic acid). Both the trap- and analytical-columns were operated at 40 °C. Separated peptides were online electrosprayed to a Q Exactive Plus (Thermo Fisher Scientific) mass spectrometer via a nano-electrospray source (positive mode, spray voltage of 2.6 kV). The MS was operated with a top 10 data-dependent acquisition strategy. Briefly, one 350 to 1,400 *m/z* full MS scan at a resolution setting of *R* = 70,000 at 200 *m/z* was followed by higher-energy collisional dissociation fragmentation (normalized collision energy of 26) of the 10 most intense ions (z: + 2 to +6) at *R* = 17,500. MS and MS/MS ion target values were 3,000,000 and 50,000 ions with 50 and 50 ms injection times, respectively. MS/MS isolation was carried out with 1.5 *m/z* isolation windows. Dynamic exclusion was limited to 30 s. Peptide match was set to “Preferred” to select isotopic features reminiscent of peptides for data-dependent scanning.

### Bioinformatic Quality Control and Initial Processing of Sequences

Raw data was returned in the form of single FASTQ files. As a preliminary step, the sequences of adaptors and indexes and poly-G tails occurring due to the specifics of the NextSeq500/550 and Hiseq2500 technology were removed from the ends of DNA sequences using AdapterRemoval2 (Schubert et al. 2016). In order to avoid spurious matches in the following analysis, we discarded all sequences shorter than 30 bp and with a quality below 20. (For further information regarding all the software used and their versions see supplementary table S14, Supplementary Material online).

### Metagenomic Screening

To further reduce the spurious assignment of generated sequences to evolutionary conserved regions, we removed low complexity sequences using prinseq and a dust value of 7 (Schmieder and Edwards 2011). Finally, we collapsed all duplicated reads using bbmap tool suite (Bushnell 2015; supplementary table S1, Supplementary Material online). Following this, an initial metagenomic screening of the deduplicated sequences was performed, using *Kraken2* (Wood et al. 2019) against the *nt* database as for 11/29/2023 (available at https://benlangmead.github.io/aws-indexes/k2) against the newly generated libraries and their skeletal element counterpart (supplementary table S15, Supplementary Material online).

For an in depth microbial metagenomic screening, we started by purging the deduplicated sequences of human reads. To do this, we mapped them against the human reference genome 37 using BWA *backtrack* (Li and Durbin 2009) with edit distance and seeding tweaked to account for aDNA damage (-n 0.01, -o 2, and -l 10,000) (Schubert et al. 2012). All sequences with mapping quality of 0 or higher were discarded, and the resultant reads were processed with KrakenUniq and the standard MicrobialDB as for 16/08/2020 (Breitwieser et al. 2018). This database includes data belonging to Bacteria, Archaea, Virus, and Eukaryotic pathogens. Additionally, it also includes the Human Reference genome 37, and a set of possible contaminant sequences.

For a more accurate detection of Eukaryotic sequences, a second screening was performed on the deduplicated sequences using KrakenUniq with a custom database. This included all published mitochondrial and plastid genomes, the human reference genome, and a dataset of known contaminants and synthetic sequences (i.e. cloning vectors, sequencing adapters, and primer sequences) as for 08/02/2022. After selecting potential targets, we performed a series of competitive mappings against their respective reference genomes in order to avoid cross mapping between phylogenetically close species (See supplementary Methods and fig. S37, Supplementary Material online). This resulted in a set of animal species filtered bam for each different sample analyzed. This pipeline was run using 8 CPUs and 20 GB of RAM memory at the Tartu University HPC. We tested the pipeline using sequences retrieved from ancient sheep bones (Z1460, Z1462, Z1463, and Z1464) as positive control, to ensure that the sequences were accurately classifiable down to genus.

### Microbial Metagenomic Analysis

Raw KrakenUniq reports were filtered by using the E-score formula described by Guellil and Borry, derived from the proportion of *kmers* by read per genome coverage in each taxonomic level (Guellil et al. 2018, 2022). Higher values of this statistic denote a higher distribution of the matching hits along the reference genome and thus, the higher the probability that the hit is genuine. We have considered as valid those microbial species with an E-score of 7 or above. To estimate the proportion of reads originating from various microbial sources, we have used Sourcetracker2, a source-prediction software with a Bayesian framework (Knights et al. 2011). The analysis with Sourcetracker was performed on normalized bacterial taxa reads abundance at the species level. We have used a custom metagenomic dataset of sources, including modern human dental calculus, human oral plaque, skin, soil, gut, and domestic animal gut (see supplementary Methods Section S1.1, figs. S38-S40, and table S15, Supplementary Material online). We then merged each filtered sample report to the reference dataset, keeping species with at least 200 reads in the Reference samples, and 50 reads in the target sample (to account for lower abundance of microbial reads in ancient samples), and discarding species with an abundance below 0.02% in the entire dataset. Sourcetracker2 was then run with our target sample as sink, using a refraction value of 100 for sinks and sources. We have used the same dataset to perform a nonmetric dimensional scaling (nMDS) from Bray–Curtis distances using R package *vegan* (Dixon 2003). Microbial contamination based on extraction blanks was estimated using decontam (Davis et al. 2018), applying a threshold of 0.7. Species susceptible to originate from contamination were subsequently discarded from downstream analysis.

Damage per source was estimated by mapping sequences to species characteristic of each source as inferred by Sourcetracker2 option *per_sink_feature_assignments*. To facilitate the process, we extracted reads by taxonomy ID using krakentools (Lu et al. 2022) and mapping those same sequences to the respective reference genome with *BWA bactrack* with astringent settings (-e 0.1). Mapped sequences were then analyzed for terminal cytosine deamination using MapDamage2 (Jónsson et al. 2013). We have calculated the average deamination ratio in the last 5 bp of the read in those species with more than 100 sequences mapped. Additionally, we analyzed GC content in the different microbial sequences retrieved (see supplementary Methods Section S1.2 and fig. S41, Supplementary Material online). To test whether the average damage for species within different environments was significantly different, we performed an ANOVA using the R-package stats (R Core Team 2023) using Soil as the reference group.

We extracted species assigned by sourcetracker2 to be characteristic of human and ruminant gut microbiomes from our samples' KrakenUniq raw reports, and from the reference's raw reports. We applied an E-score filter of ≥7 and read number of ≥10. Once the individual reports were filtered, we merged all the reports into a merged dataset, normalizing for sample library size, and discarding in the process species with a representation below 0.02% in the total of the dataset. This filtered dataset was further normalized using a *clr* transformation in the R package compositions (van den Boogaart and Tolosana-Delgado 2008), and a PCA was computed using R package *mixOmics* (Rohart et al. 2017). Resultant sample values and loadings were visualized using R. Microbial species of interest were filtered from the raw KrakenUniq report using the same E-score formula. We then extracted them from the raw fastq using krakentools and validated using a combination of megablast and MEGAN6 (Chen et al. 2015; Beier et al. 2017).

### Modern and Ancient Contamination

We have tested the presence of possible contamination sources in extraction blanks that could alter our results. To do that, we performed competitive mappings against all the target species found using the pipeline described in this article. Those reveal traces of dog (*Canis lupus*), turkey (*Meleagris gallopavo*), and human sequences (supplementary table S10D, Supplementary Material online). Those results were validated through the usage of Blast, assigning taxons to hits with a presence over 80% with MEGAN6 (Beier et al. 2017). aDNA damage patterns were characterized with MapDamage2. Since those sequences are scarce and do not present aDNA damage, we consider that they do not significantly impact the results of our analysis.

We used schmutzi to detect the presence of modern contamination in our mapped sequences. This method considers the endogenous deamination rates of the sequences (Renaud et al. 2015). We applied schmutzi to both nuclear and mitochondrial human sequences. Is important to state that schmutzi estimates can be inaccurate in when the number of sequences is low, such in the case of our samples. Is for that reason that we additionally checked mitochondrial contamination in CHRY038B and CHRY038 by manually annotating mitochondrial variants using the Integrative Genome Viewer (IGV) (Robinson et al. 2011), variants susceptible to be caused by contamination in the process (supplementary table S6C, Supplementary Material online). The contamination was calculated as the ratio of allele depth between the majority and minority allele at the annotated positions. CHRY038 revealed a contamination rate of 0.597%, while CHRY038B had a rate of 0%.

For the retrieved *R. rattus* sequences, we sought to determine if they were from a single individual, or from different specimens of black rat. To accomplish this, we generated callings for the *Rattus* mitochondria using GATK UnifiedGenotyper (McKenna et al. 2010). Afterwards, we checked the heterozygosity ratio in biallelic positions with ≥ 5 × depth, discarding those that could be originating from aDNA transition patterns (C ↔ T or G ↔ A).

### Uniparental Markers and Sex Determination Analysis

For human sequences, we called rCRS variants using GATK UnifedGenotyper, followed by creating a consensus fasta sequence with bcftools (Danecek et al. 2021). We determined the mitochondrial haplogroup using haplogrep3 (Schönherr et al. 2023). All sediment samples except CHRY038B present a haplogroup H2a2a1, haplogroup of the rCRS, indicating not enough mitochondrial sequences to estimate an haplogroup (supplementary table S6D, Supplementary Material online). To determine the genetic sex of the samples, we have used the ry_compute, considering ratio values below 0.016 as female, above 0.077 as male, and values in between as undeterminable (Skoglund et al. 2013).

For nonhuman animals, we computed the ratio of the normalized coverage in the X chromosome and the normalized coverage in the Y chromosome. Values above 1 are indicative of a male individual, while values close to 0 are indicative of a female.

### *Rattus Rattus* Dataset Building and Population Genetics Analysis

With the intention of studying the genetic background of the black rat sequences found in NMS022B, we started by creating a population genetics dataset. First, we downloaded and mapped publicly available data from *R. rattus* and *R. tanezumi* (Puckett et al. 2016; Teng et al. 2016; Massini Espino et al. 2022; Yu et al. 2022). For modern data, we trimmed the sequencing adapters, keeping sequences up to 30 bp and merging paired end reads when necessary. Following this, we used *BWA mem* with default parameters to map the reads against the *R. rattus* reference genome *Rrattus_CSIRO*. Afterwards, duplicated sequences were removed by coordinate using picard and sequences with a mapping quality equal or above 30 were kept for downstream analysis. For ancient samples, we followed the same procedure, using BWA backtrack (-l 65536 -n −0.01 -o 2 -q 0) instead of the *mem* algorithm. Basic statistics were generated using Qualimap2 (Okonechnikov et al. 2016).

We selected a total of 52 individuals (including NMS022B) with more than 0.01 × for population genetic analysis. First, we generated genotype likelihoods using angsd (Korneliussen et al. 2014) with the following options “*-uniqueOnly 1 -remove_bads 1 -trim 5 -C 50 -baq 1 -minInd 30 -nThreads 10 -skipTriallelic 1 -GL 2 -minMapQ 30 -doGlf 2 -doMajorMinor 1 -doMaf 2 -minMaf 0*.*05 -SNP_pval 1e-6*.” The resultant dataset contained 513,655 positions. We then computed a PCA using PCAngsd with 100,000 iterations (Meisner and Albrechtsen 2018). We also generated a dataset of pseudohaploid callings using angsd with the options “*-dohaplocall 1 -doCounts 1 -minMinor 0 -uniqueOnly 1 -remove_bads 1 -minMCQ 30 -minInd 40 -trim 5*,” and removed transitions, resulting in 47,828 biallelic positions. This pseudohaploid dataset was used to compute the *f4* analysis, available in AdmixtTools as *qpDstat* (Patterson et al. 2012). Population clusters described by Yu et al. (2022) were used. We tested the following tree (Rattus tanemuzi, NMS022B, Modern_Canada, X). We also tested the following tree configuration: (Rattus tanemuzi, NMS022B, Modern_Denmark, X), (Rattus tanemuzi, NMS022B, Modern_Argentina, X), and (Rattus tanemuzi, NMS022B, Modern_USA, X). Statistical significance was assessed using block jack-knife resampling.

### *Rattus Rattus* Mitochondrial Phylogeny

We used GATK UnifiedGenotyper to generate callings for all published samples and NMS022B. For the mitochondrial phylogeny, we selected biallelic SNPs with a depth ≥5 × and a genotype calling quality of ≥20. For heterozygous positions, we kept all positions with allele depth proportion of 1/9 as homozygous for the majority allele, discarding all the others in the process. Consensus mitochondrial sequences were generated for each sample with bcftools, using the filtered sets of SNPs and the *R. rattus* mitochondrial reference genome. To finalize the creation of the dataset, we masked in each sample those positions, which did not meet the filtering criteria with an N. This accounted for a total of 3,467 variable positions in the dataset.

A maximum-likelihood (ML) phylogenetic tree was created using RAxML (Stamatakis 2014). We used the GTR-GAMMA substitution model, with 100 bootstraps and *R. tanezumi* isolate RJPNAna02 as an outgroup. The resultant tree was visualized using ggtree (Yu et al. 2017).

### Human Variant Calling Genetic Imputation

Published human data from the Cherry Hinton site were mapped using *BWA bactkrack* as stated above (Hui et al. 2024). We then generated pseudohaploid callings of the published individuals and the filtered data of the sediment sample CHRY038B using *pileupcaller* (available at https://github.com/stschiff/sequenceTools) and the HO dataset as calling panel (Lazaridis et al. 2014). Dataset manipulation was done using PLINK (Purcell et al. 2007). Terminal damage postmortem deamination was estimated using MapDamage2, for both human sequences filtered from sediment and from the original published data. We compared terminal deamination levels in sediments versus skeletal elements using a Welch two sample *t*-test (implemented in R as *t*.*test*) (R Core Team 2023).

We imputed genotypes in both CHRY038B (average genomic coverage 0.01×) and CHRY038 (average genomic coverage 0.06×) samples using QUILT, a tool developed to work with low-coverage whole-genome sequences (Davies et al. 2021) and the HRC reference panel (McCarthy et al. 2016). To speed up the process, each reference chromosome was split into the overlapping chunks (*N* = 771 altogether), which were merged after imputation chromosome-wise using bcftools concat –ligate option (Danecek et al. 2021). Postimputation data processing included: (a) correction of the conflicting genotypes (GT) by GP (gp-to-get option in the bcftools tag2tag plugin); filtering out positions with MAF <5% in the HRC reference panel resulting in 5.5 M positions. Additionally, we kept only positions with GP 98% and higher (GP >0.98); this filter was applied individually at each genome that gave 51,641and 468,426 positions for CHRY038B and CHRY038, respectively. The final number of positions that overlapped with the HO dataset was 24,469 and 224,460 for CHRY038B and CHRY038, respectively (supplementary fig. S42, Supplementary Material online). As genotypes for both ancient samples were imputed from the genomes with sequence coverage (<0.1×), which is below the one indicated suitable for imputation (Hui et al. 2020), we consider our results as suggestive.

### Human Population Genetic Analysis

A PCA using 442 modern European individuals from the HO dataset was computed using *smartpca*, with our samples CHRY038B and CHRY038 projected. We used options *shrinkmode: YES*, and *outliermode: 2*. Ellipses describing 95% confidence intervals for the projected samples were generated with *optionellconf: 0*.*95*. We repeated the process including CHRY038B and CHRY038 at different stages of imputation (Raw Pseudo-Haploid callings, GP > 95 and GP > 98).

We assessed relatedness (in this particular case, if CHRY038 and CHRY038B) were identical using READv2 with default parameters (Alaçamlı et al. 2024), with our samples at different stages of imputation and 20 additional samples from the Cherry Hinton site (supplementary table S17, Supplementary Material online).

### Human Phenotype Prediction

Using the imputed genomes of CHRY038B and CHRY038 (the original sample), we extracted genotype calls for 39 of the 41 HIrisPlex-S (Chaitanya et al. 2018) variants and 74 SNPs involved in diet and diseases, coding the allele information as the number of the effective allele (0, 1, 2) using plink (supplementary table S6A, Supplementary Material online). A DS of 0,1 was used, which is the default value in plink. The diet and disease set of all 74 imputed SNPs was selected starting from lists of variants previously analyzed in aDNA studies (Olalde et al. 2014; Allentoft et al. 2015; Günther et al. 2018) prioritizing those with a role in the response to pathogenic infection. A table with the HIrisPlex-S SNP alleles per individual was uploaded on the HIrisPlex-S webtool (https://hirisplex.erasmusmc.nl/) to obtain probabilities values for eye, hair, and skin color category. This output was then interpreted following the manual to obtain the final pigmentation prediction for the original skeletal element and for the genomes from sediments (supplementary table S6B, Supplementary Material online).

### Protein Database Search and Result Filtering

Raw mass spectrometry data were searched for each samples using pFind (Chi et al. 2018; Shao et al. 2021) against all proteins in SwissProt (Release 2017/01), which contains 553,474 protein sequence entries, and the cRAP database (as for 04/03/2019) (https://www.thegpm.org/crap/). For the search, fixed modification was set to include carbamidomethylation of cysteine and variable post-translational modifications (PTMs) were set to include proline hydroxylation, glutamine and asparagine deamidation, methionine oxidation, and pyroglutamate formation from glutamine and glutamic acid. Searches were conducted with trypsin full-specific digestion. Precursor mass tolerance was set to 15 ppm and fragment mass tolerance to 0.02 Da and the false-discovery rate of PSMs equal ≤1.0%. pFind peptides were filtered based on their score. Only peptides with a score below or equal to 0.01 and at least two PSMs were considered. Protein matches were considered if they had at least two unique peptide matches. All contaminants from our samples were identified and removed using the peptide identified within our extraction blank and the cRAP database. Additionally, we compared pFind results with *novor*.*cloud* (Ma 2015) another proteomics mass spectrometry analysis software commonly used (See supplementary methods section S3, Supplementary Material online). All peptides were blasted against SwissProt using BlastP (Altschul et al. 1990) and visualized with MEGAN (version 6.24.6, built 16 Nov 2022) and analyzed using Unipept (Unipept 6.2.5 using UniProt 2025.02) (van de Moortele et al. 2024). All the key results were checked manually using BlastP. No pathogen proteins with a 100% identity and coverage were found.

### Post-translational Modifications Analysis

We measured the proportion of all identified post-translational modifications (PTMs) relative to the total number of amino acids (supplementary fig. S43, Supplementary Material online). We quantified in particular methionine oxidation and glutamine/asparagine deamidation along with the peptide length distributions.

### *Rattus Rattus* Peptide Analysis

For the search of rat peptides in the NMS022B sample, we built an in-house database with protein sequences obtained from the reference genome of *R. rattus*_CSIRO and *Rattus norvegicus*_GRCr8 (The SwissProt database contains only reference protein sequences of *R*. *norvegicus*) (supplementary File S1, Supplementary Material online). Next, all filtered peptides were searched against this custom database and NCBI_nr using the same parameters as before. In order to identify specific-species peptides, we blasted all the identified peptides using BlastP against the ncbi nr database (Altschul et al. 1990). Filtered results (100% of identity and coverage) were parsed on MEGAN. We used two species-specific peptide sequences from collagen alpha-2(I) chain, and alpha-2 HS-glycoprotein isoform X1 proteins to assign the NMS022B sample peptides within the genera of European *Rattus*. No rodent peptides were found in the extraction blank.

### Limitations

The main limitation of the study is that it is based on few individuals from each site. In addition, the storage conditions, the initial burial environment might have had an impact on the subsequent analysis. In the case anyone would want to replicate the study, we suggest picking different samples from each site and drawing comparisons between sites and sample types.

As this was a pilot study, we applied standard protocols for aDNA extraction and library building, but other protocols might yield better results. Moreover, regarding proteomics analysis, we acknowledge that there is a bias in the database used, as it is mostly used for clinical studies.

Regarding metaproteomics analysis, a key limitation of using SwissProt for identifying bacterial peptides from soil samples is its low representation of soil microbes, which can result in the under identification of bacterial proteins, along with limited coverage of plant and animal taxa, primarily focusing on model organisms and economically important species, leading to an underestimation of taxa identified through proteomics.

## Supplementary Material

msaf202_Supplementary_Data

## Data Availability

The datasets generated and analyzed during the current study are available in the ENA repository under the accession ID: PRJEB81700. Genomic data from the original skeletal elements (previously published) is available in the ENA repository under the accession ID: PRJEB59976, and the data depository of the EBC (https://evolbio.ut.ee/). The mass spectrometry proteomics data have been deposited to the ProteomeXchange Consortium via the PRIDE [Perez-Riverol Y, Bai J, Bandla C, Hewapathirana S, García-Seisdedos D, Kamatchinathan S, Kundu D, Prakash A, Frericks-Zipper A, Eisenacher M, Walzer M, Wang S, Brazma A, Vizcaíno JA (2022). The PRIDE database resources in 2022: A Hub for mass spectrometry-based proteomics evidence. Nucleic Acids Res 50 (D1): D543-D552 (PubMed ID: 34723319).] partner repository with the dataset identifier PXD057056. Custom scripts used in the study are available in GitHub (https://github.com/tonidedios94). All data needed to evaluate the conclusions in the paper are present in the paper and/or the supplementary materials, Supplementary Material online.
