# Supplementary material for: Bone Adhered Sediments as a Source of Target and Environmental DNA and Proteins
Source: Mol Biol Evol. 2025 Aug 26;42(9):msaf202. doi: 10.1093/molbev/msaf202 (PMC12409277; doi:10.1093/molbev/msaf202)
Supplement: msaf202_Supplementary_Data [file msaf202_supplementary_data.zip › Revision_Supplementary (1).pdf]

Supplementary Materials: Bone adhered sediments as a source of target and environmental DNA and proteins

Toni de-Dios, Biancamaria Bonucci, Rémi Barbieri, Alena Kushniarevich, Eugenia D'Atanasio, Jenna M. Dittmar, Craig Cessford, Anu Solnik, John E. Robb, Christina Warinner, Ester Oras, Christiana L. Scheib

Contents

Supplementary Information 3

SI1 - Archaeological sites 3

SI1.1 Trumpington Meadows 3

SI1.2 Duxford 5

SI1.3 Barrington Edix Hill A 9

SI1.4 Gamlingay 12

SI1.5 Cherry Hinton 15

SI1.6 St John’s Divinity School / Hospital of St John, Cambridge 18

SI1.7 New Museums Site / Augustinian Friary, Cambridge 21

Supplementary Methods 24

SM1 Metagenomic Microbial analysis 24

SM1.1 Reference dataset creation 24

SM1.2 GC content analysis 25

SM2 Eukaryote DNA Screening 26

SM2.1 KrakenUniq dataset creation 26

SM2.2 Screening 27

SM2.3 Whole genome mapping and quality control 28

SM3 Proteomic analysis with novor.cloud 29

Supplementary Figures (Figures S14 to S42) 30

Supplementary Tables 60

Supplementary Data 72

Supplementary Bibliography 73

Supplementary Information:

SI1 Archaeological sites

SI1.1 Trumpington Meadows

See also descriptions in (Patten 2012; Scheib et al. 2019).

The site of Trumpington Meadows is located southwest of Cambridge (Figure S1). The site was excavated by the Cambridge Archaeological Unit in 2010 and 2011, previous to land development of the area. These excavations revealed the presence of archaeological remains from 3 different periods: Neolithic, Iron Age and Anglo-Saxon. In particular, the Neolithic activity in Area C consisted in two burial monuments (Monument I and Monument II), pits, and a possible spring. Monument II contained 3 individuals. TRM003 (F.243-Sk801) was radiocarbon dated to 3703 - 3641 calBC. A petrous bone was sampled for population genetic analysis (Scheib et al. 2019), while sediments attached to this skeletal element were also sampled for this study. The burials present evidence of post-depositional disturbance. Given the arrangement of the remains and the partially articulated and disarticulated elements, it has been suggested that the grave was reopened and used for some time afterwards, explaining the movement of the bodies. Upper fillings of the monument reveal the presence of a Collared Urn vessel, indicating that the monument was still in use up until the Bronze Age. Numerous animals remain were found in Monument II, including skeletal pieces of domestic animals such as pig, cow, dog; and remains of wild animal species such as auroch, deer, fox, boar and toad/frog.

TRM003 are the remains of an adult male. Is located southernmost inside Monument II, and is stratigraphically, non-related to the other remains in the monument. The skeletal remains of the individual have been heavily truncated or disturbed, the body is sectioned from below the pelvis. A partial mandible (F254-Sk803) is situated 50 cm apart from the remains and could belong to the same individual. An arrowhead was recovered during the sampling of the individual. A pig skeletal part was found in the same burial area.

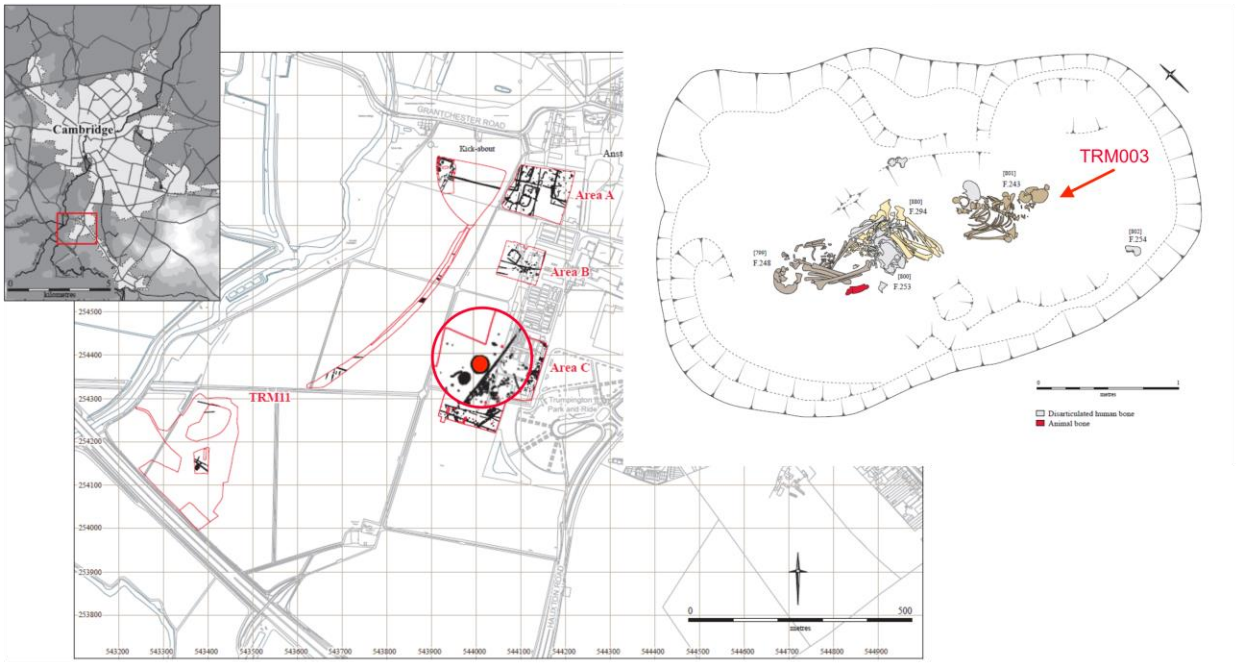

Fig S1. Location of Trumpington site in the Cambridge Area. In particular TRM003 is located in Monument II (Red dot indicated by the red circle in Area C). Disposition of TRM003 in Monument II (indicated with a red arrow). Adapted from Patten 2012 (Patten 2012).

SI1.2 Duxford

See also descriptions in (Lyons 2011; Scheib et al. 2024).

The archaeological site of Duxford is situated 11km south-east of the city of Cambridge, in the west bank of the river Granta. Site evaluation was initiated in 2001, in preparation for residential development of the area. Prior to this, the land was used as an old rectory, and in addition to that, considerable construction was done during the 1970s. During site evaluation, remains dating back to the Iron Age, Early Roman period, Anglo-Saxon, Medieval and post-Medieval period were discovered. The excavation was initiated in March 2002 by CAM ARC (now Oxford Archaeology East).

The 2 analysed samples (DUX010 and DUX012) date back to the late Iron Age - Romano British period of occupation of the site. In particular, petrous bones (Scheib et al. 2023) and sediment attached to them were sampled for both individuals. During the late Iron Age and early Roman occupation, Duxford and surrounding sites were a primarily agricultural area, with extensive field systems (Cambridgeshire Historic Environment Record - CHER 9741). During the Roman occupation, the area experienced a development of the area, mainly due its closeness to two Roman roads (north and east of the site), and two branches of the Icknield Way (Kirby and Oosthuizen, n.d.). The change in land use is reflected in the adoption of the Roman Villa farming system. Several Roman settlements and constructions are found in the close vicinity of Duxford, mainly 5 - 6 potential settlements, 2 villas, 2 farmsteads and a field system, reflecting the rural background of the area (CHER 4142; 4314; 8822; 11687; 11978a; 4153; 14689). The area saw agricultural use during the Anglo-Saxon period although with more signs of nucleation (Taylor 2002), and the

establishment of different manors and 2 parish churches during the Medieval Period (CHER 4698; 14835; 1263-5).

The analysed samples, DUX010 and DUX012 were excavated from a northern part of the site, dating to the late Iron Age and early Roman period (Figure S2). Close archaeological features (Ditch 8, Ditches 9-13, Pits 8-11) present (apart from pottery and other archaeological artefacts) and abundant animal skeletal remains, including domestic species such as cattle, sheep/goat, horse, pig, and dog; but notably also wild animals such as deer, toad (most certainly intrusional) or fish. The use of the Pits is theorised to be for feast waste disposal.

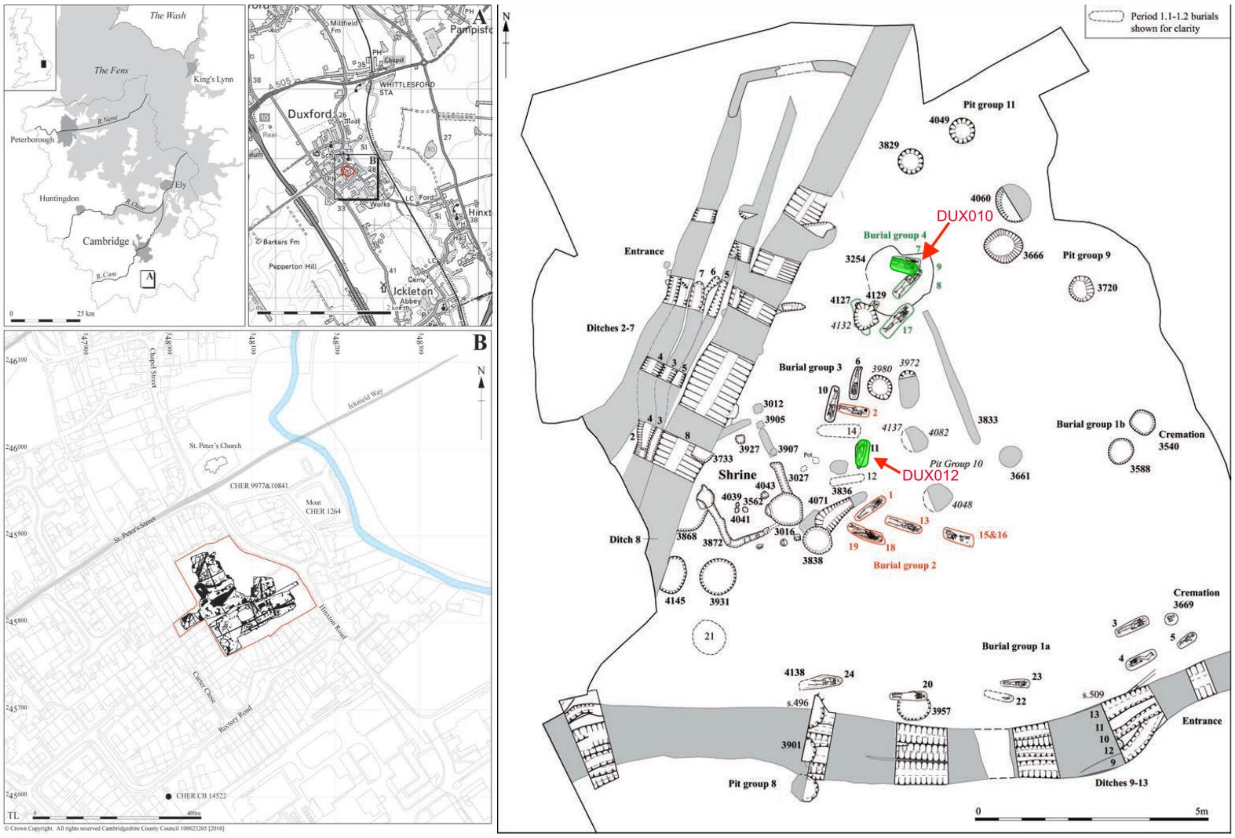

Fig S2. Location of Duxford site. Location of samples analysed (indicated with a red arrow and circle) in the northern part of the site. Adapted from Lyons 2011 (Lyons 2011).

DUX010 (Burial 9), is part of burial group 4 (Burials 7, 8, 9 and 17; Graves 4127 and 4129). Burial 9 was radiocarbon dated to the Roman period (40 BC - 240 AD). The skeletal remains were determined to belong to a male of an age of 35 years old (23 - 57), a 1,70m in height, and with pathologies in the form of arthritis and poor oral health. The burial contained middle Iron Age pottery sherds. Burial 9 was deposited overlapping the earlier Burial 8, intersecting part of it (Figure S3). Burial 8 contained, apart from middle Iron Age pottery fragments, sheep/goat teeth and heel bone, a cattle tooth, and the rib of an unidentified large mammal.

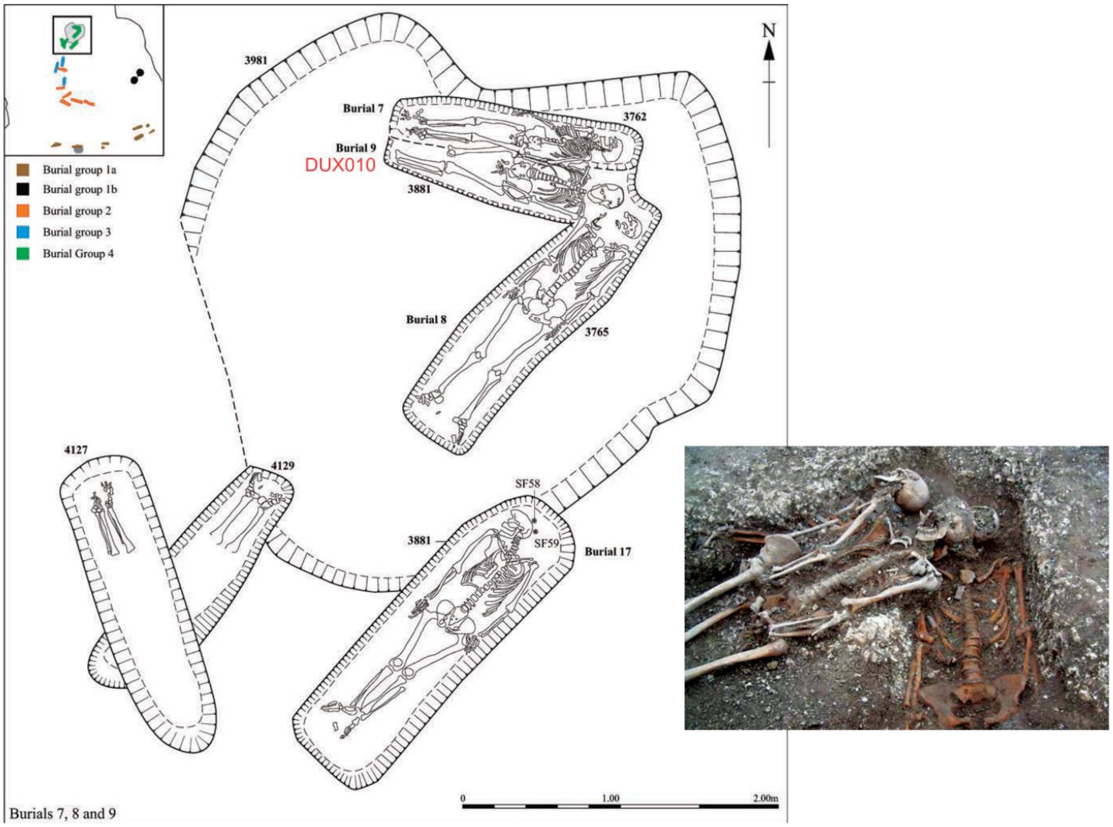

Fig S3. Details of the burial group 4. Adapted from Lyons 2011 (Lyons 2011). Indicated in red, the individual of interest.

DUX012 (Burial 11), is part of the burial group 3 (Burials 6,10 and 11; burial 10 radiocarbon dated as Roman; 80-380 AD). Burial 11 was located to the south of Burials 6 and 10 sharing the same orientation (Figure S4). Archaeological features suggest that Burial 11 could have been contemporary with Burials 6 and 10. The grave contained the skeletal remains of an adult ?male, whose age and stature could not be determined due to the fact that their feet and lower legs were sectioned during machinery. Inspection of the remains showed apparent evidence of vertebral fusion, due to pathology or congenital deformity. In the silt surrounding the skeleton several animal remains have been found. Those include horse teeth and phalanx bones, a cattle ischium, and a sheep tibia. From within the grave several materials thought to be already present at the time of deposition were found. Those were small amounts of cereal grains, charcoal, a black tarry material, bone and small coal fragments. A later medieval horseshoe was found within the grave fill and was probably deposited during the previous installation of a modern pipe east from the grave. Close to Burial 11, we can find earlier burials (middle Iron Age), 14 and 12. In particular Burial 14 also presented grains (*Hordeum spp.)* and a cattle heel bone.

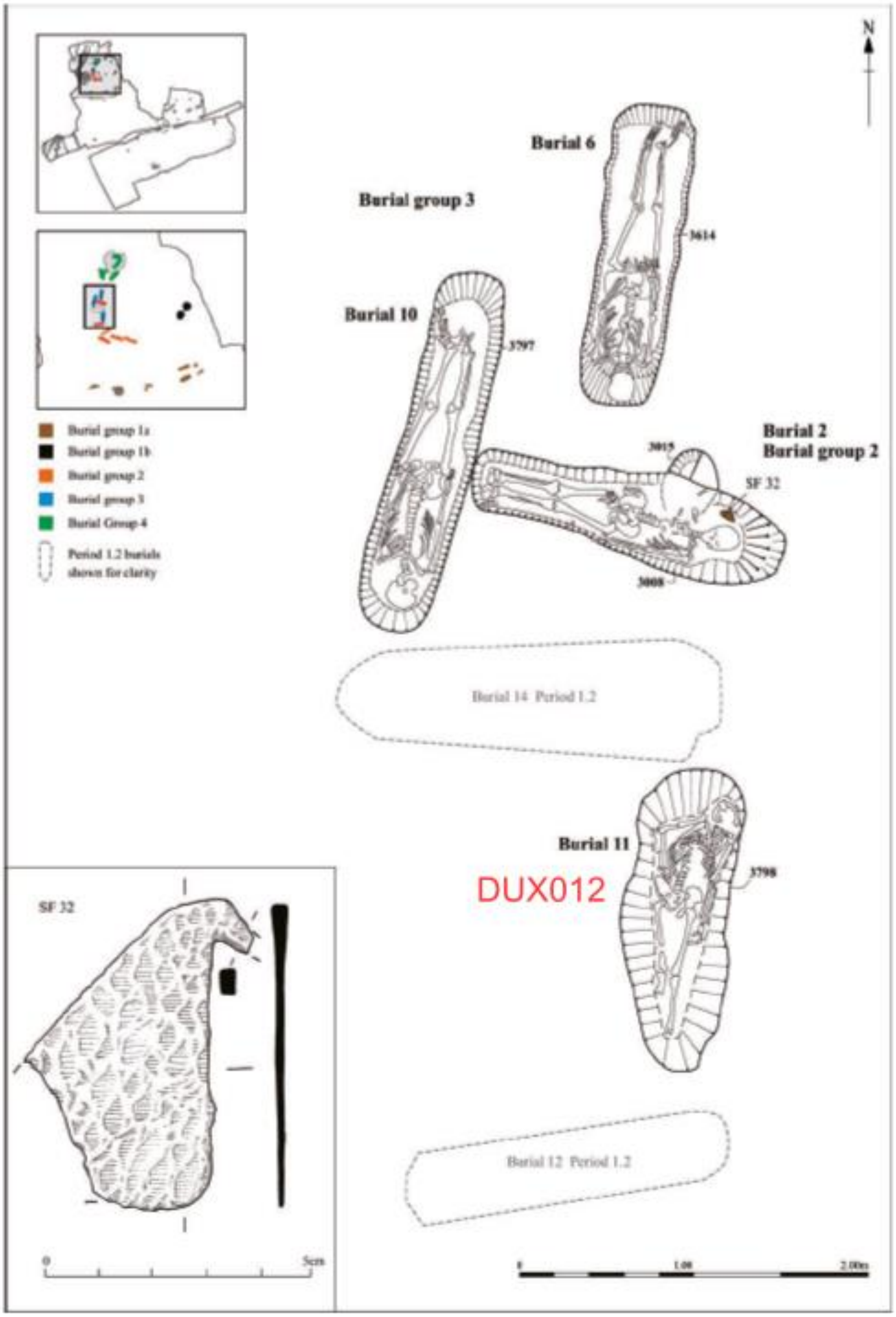

Fig S4. Details of the burial group 3. Adapted from Lyons 2011 (Lyons 2011). In red, the individual of interest.

SI1.3 Barrington Edix Hill A

See also descriptions in (Malim 1998; Guellil, Keller, et al. 2022).

The site of Edix Hill (Barrington, Cambridgeshire, UK) is an Anglo-Saxon cemetery dating between 500 and 650 CE (Malim 1998; Guellil, Keller, et al. 2022). A total of 149 individuals from 115 graves were analyzed (Figure S5), revealing a demographically representative population with equal sex distribution. Osteological data suggest a robust community with prevalent osteoarthritis, indicative of intensive manual labour. Burial goods indicate a material culture blending Anglian, Saxon, and Kentish traditions, with four distinct costume groups. The site contained one barrow mound, multiple grave markers, and various wooden grave structures. Grave orientations varied, likely influenced by topography and pre-existing Iron Age features.

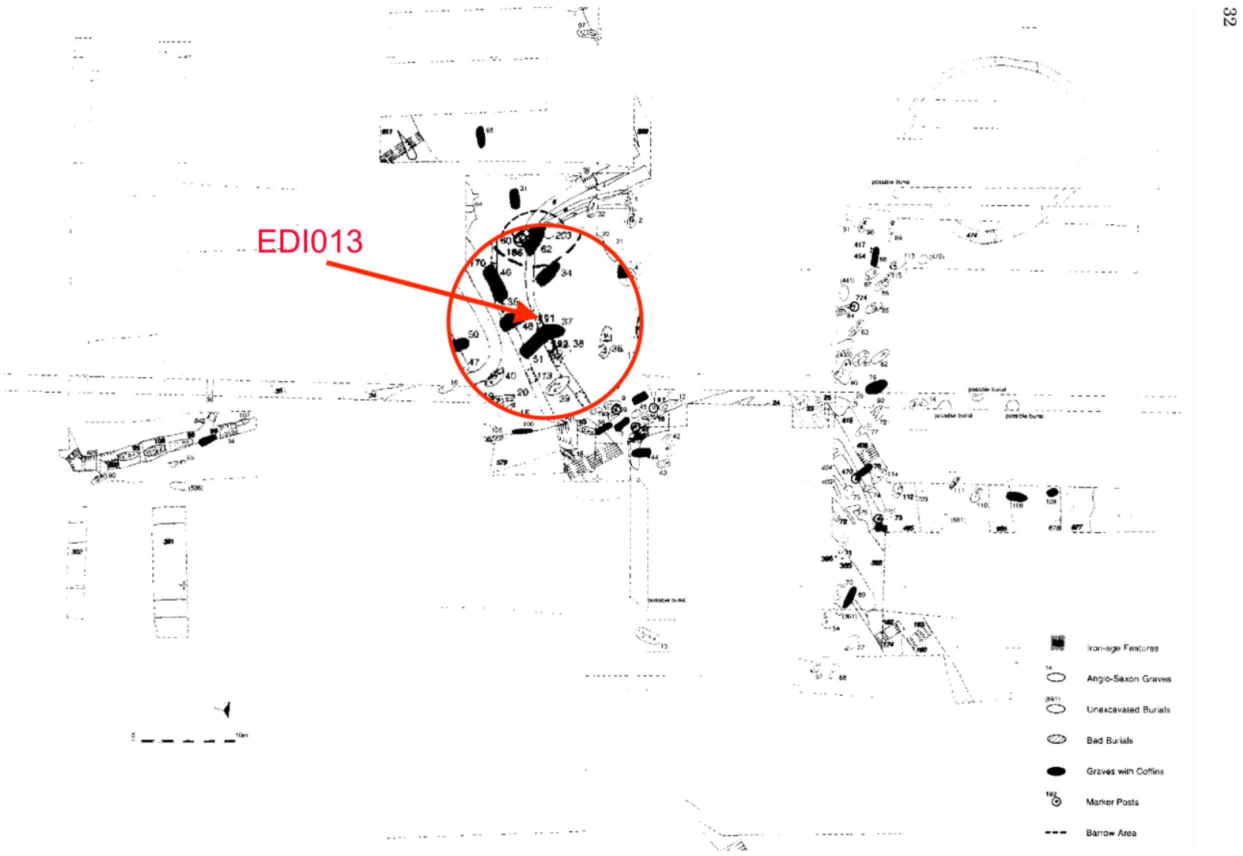

Fig S5. Distribution of graves in Edix Hill cemetery. The sample studied in the paper is indicated with a red arrow and a circle. Modified from Malim 1998 ((Malim 1998; Guellil, Keller, et al. 2022).

EDI013 (Feature 126, Skeleton 126, Grave 37), an adult female (45+ years old), of 1.64 m of height, with evidence of arthritis and dental disease, was found 0.23 m below ground (Figure S6). Petrous bone from this individual was sampled by Guellil 2022, for which we also retrieve sediments. The edges of this grave and grave 51 were very clear where they were cut into chalk, but at their interface they overlay an Iron Age ditch (F114) and it was very difficult to trace the full extent of the graves in the ditch fill. EDI013 appears to be earlier and deeper than grave 51, extending as a wide, sub-rectangular scoop in the chalk. The skeleton was largely intact, and the vertical position of the skull shows that it must have slumped down into the grave during decomposition, as can also be seen by the brooch which was found face down. Most of the feet bones are missing because they were slightly higher than the main part of the body and had rested on the edge of the grave. Such size and regularity in the grave-cut together with evidence for movement during decomposition suggests use of a coffin with the feet possibly resting against it.

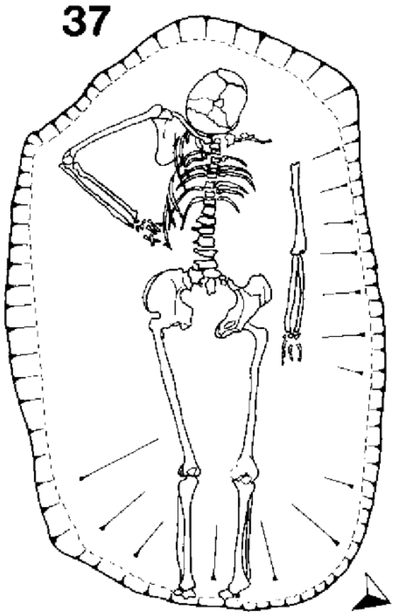

Fig S6. Disposition of the skeleton in grave 37. Adapted from Malim 1998(Malim 1998; Guellil, Keller, et al. 2022).

SI1.4 Gamlingay

See also descriptions in Murray and Macdonald 2005 (Murray and McDonald 2017).

Gamlingay is located 22 km west-southwest of Cambridge (Figure S7) and the Station Road site was excavated in 1997 by the Hertfordshire Archaeological Trust (now Archaeological Solutions) (Murray and McDonald 2006), although elements of the publication have been challenged (Hamerow 2012).

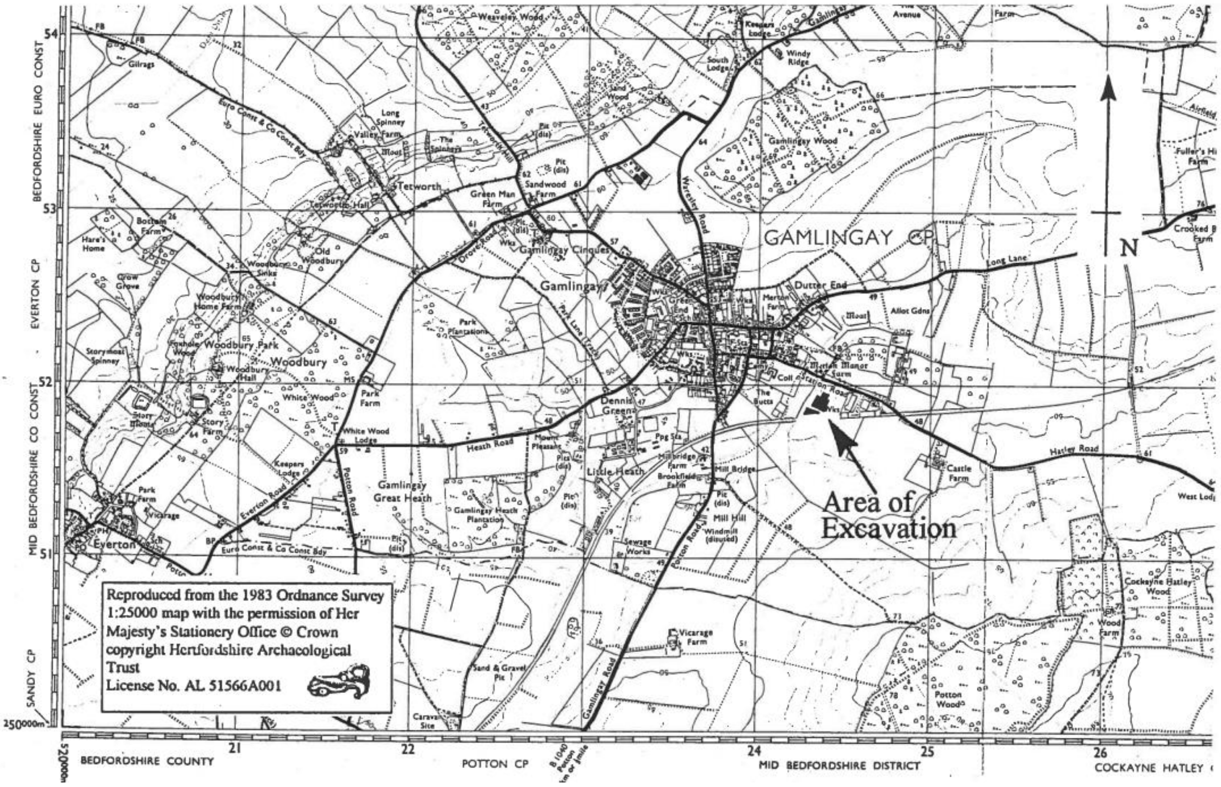

Figure S7. General site location plan (1:250,000) from McDonald, T. and Trevarthen, M., 1998 Excavations at Station Road, Gamlingay, Cambridgeshire. Interim Site Narrative (Unpublished report). SCB17202 (McDonald and Trevarthen 1998).

There is evidence for four or five phases of Anglo-Saxon activity. The occupational sequence begins with a ditched enclosure and some sunken featured buildings (Phase A), which were dated by the presence of a small quantity of fifth–sixth century Early Anglo-Saxon pottery. The suggestion that an Early Anglo-Saxon settlement had ditched enclosures is unlikely and the pottery is probably residual (Hamerow, H., 2012, p. 71 note 3)(Hamerow 2012). Whilst it is possible that some of the sunken featured buildings date to the Early Anglo-Saxon period, the Phase A occupation probably begins no earlier than the seventh century. This was followed by some rectilinear enclosures with timber buildings (Phase B) and then a later rectilinear enclosure (Phase C). After this, there was a curvilinear enclosure, located largely outside the excavated area, and two sunken featured buildings (Phase D).

The burials comprised a group of dispersed inhumations and a small formal cemetery (Figure S8). The six dispersed inhumations were located on the southern fringe of the Middle Anglo-Saxon settlement. One of these burials cut through the back-filled corner of a disused Phase B timber building and was aligned along the wall trench. These dispersed burials appear to relate to either Phase C or D of the settlement.

Located within one of the Phase C enclosures and respecting its layout and alignment is a cemetery (Figure S8). The small formal cemetery consists of approximately 110 burials covering an area of *c.* 40m by 40. The burials were in shallow rectangular east-northeast to west-southwest aligned cuts with rounded ends, the bodies were in an extended supine position with their heads to the west. A number of the graves intercut, and, in some instances, earlier skeletons have been truncated

by this. Some of the skeletons of the cemetery were dated, giving the possibility to firmly place the cemetery in the context of Late Saxon burial (for an overview see Hadley, D.M., 2010) (Cherryson and Buckberry 2010).

A rib from GAM042 (skeleton 2209, burial 48, feature 2208) and sediment attached to it were sampled for the study. The fill of the grave presented 5 sherds and 30 fragments of animal bones (76 grams).

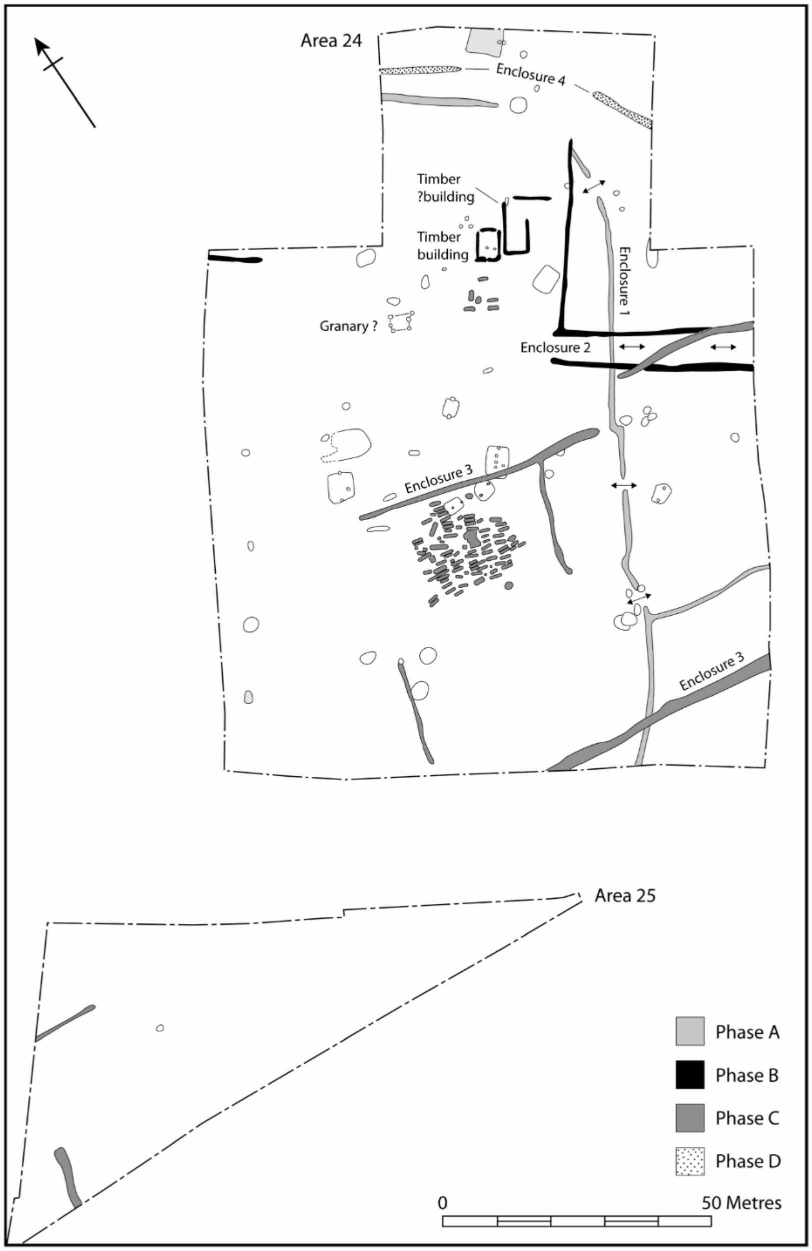

Figure S8. Site of Gamlingay with the location of burials highlighted in red. Adapted from Hamerow 2012.

SI1.5 Cherry Hinton

See also descriptions in (Cessford and Dickens 2006; Ferrante di Ruffano and Waldron 2006; Cessford and Slater 2015; Cessford et al. 2021; Pfrengle et al. 2021; Hui et al. 2024).

The settlement of Church End Cherry Hinton (Cherry Hinton) is located approximately 6 km southeast of Cambridge (Figure S9). Around the late 9th to the mid-10th century, a large aristocratic or proto-manorial center was created (Cessford and Dickens 2006). In 1999, and prior to land development, the timber chapel and graveyard were excavated by the Hertfordshire Archaeological Trust (subsequently Archaeological Solutions and now Wardell Armstrong) (Hertfordshire Archaeological Trust 1999). The burials were dated using archaeological and isotopic techniques. The skeletons were first studied by the Hertfordshire Archaeological Trust and later re-examined by the After the Plague project. From the total population of the cemetery (estimations between 1000 and 2000 individuals), only a sub-sample of 670 and 980 individuals were analysed. The main hypothesis is that the cemetery gave service to a large Late Saxon to Norman rural community. Multiple animal bones were found in the site, predominantly cattle, but also horse, dog, then sheep/goat, pig, cat, red deer, chicken and crow (Cessford and Slater 2015). Petrous bones from individuals CHRY038 and CHRY051 were sample for population genetic analysis (Hui et al. 2024), while sediments adhered to those skeletal elements were retrieved for this study.

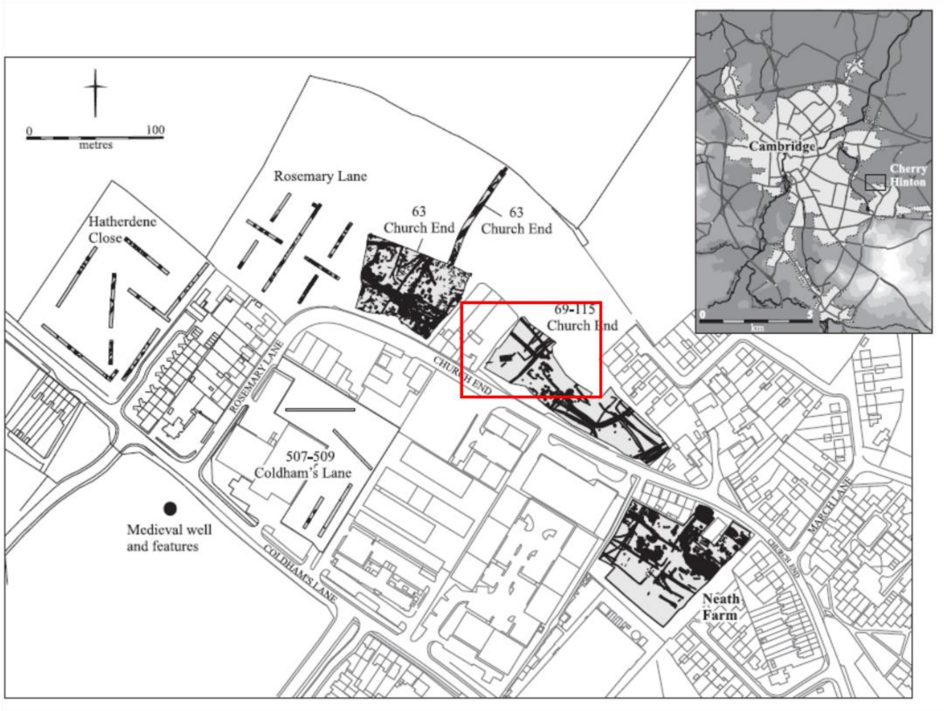

Fig S9. Location of the Cherry Hinton Site. The location of the Cemetery is highlighted in red. Modified from Cessford and Slater 2015 (Cessford and Slater 2015).

The grave containing CHRY051 (SK3463) was cut through an earlier grave containing SK3505 (Figure S10). It is probable that some of the soil in the grave containing SK3463 was filled with the earlier grave, so contamination is certainly possible. SK3505 was incomplete (described as fragmentary skull and post-cranium) so some disturbance of the skeleton by the later burial is likely. The only palaeopathology mentioned by Ferrante di Ruffano and Waldron. (Ferrante di Ruffano and Waldron 2006) for SK3505 are 'Uneven tooth wear; 3 rib fractures; ear infection; OA; Calculus; Alveolar disease; Abscess' and overall, they state that 'No direct evidence was found for infection by syphilitic, leprous or other organisms' for the whole Cherry Hinton

population. While not necessarily reliable, it does suggest that there were not obvious leprous skeletal changes to SK3505.

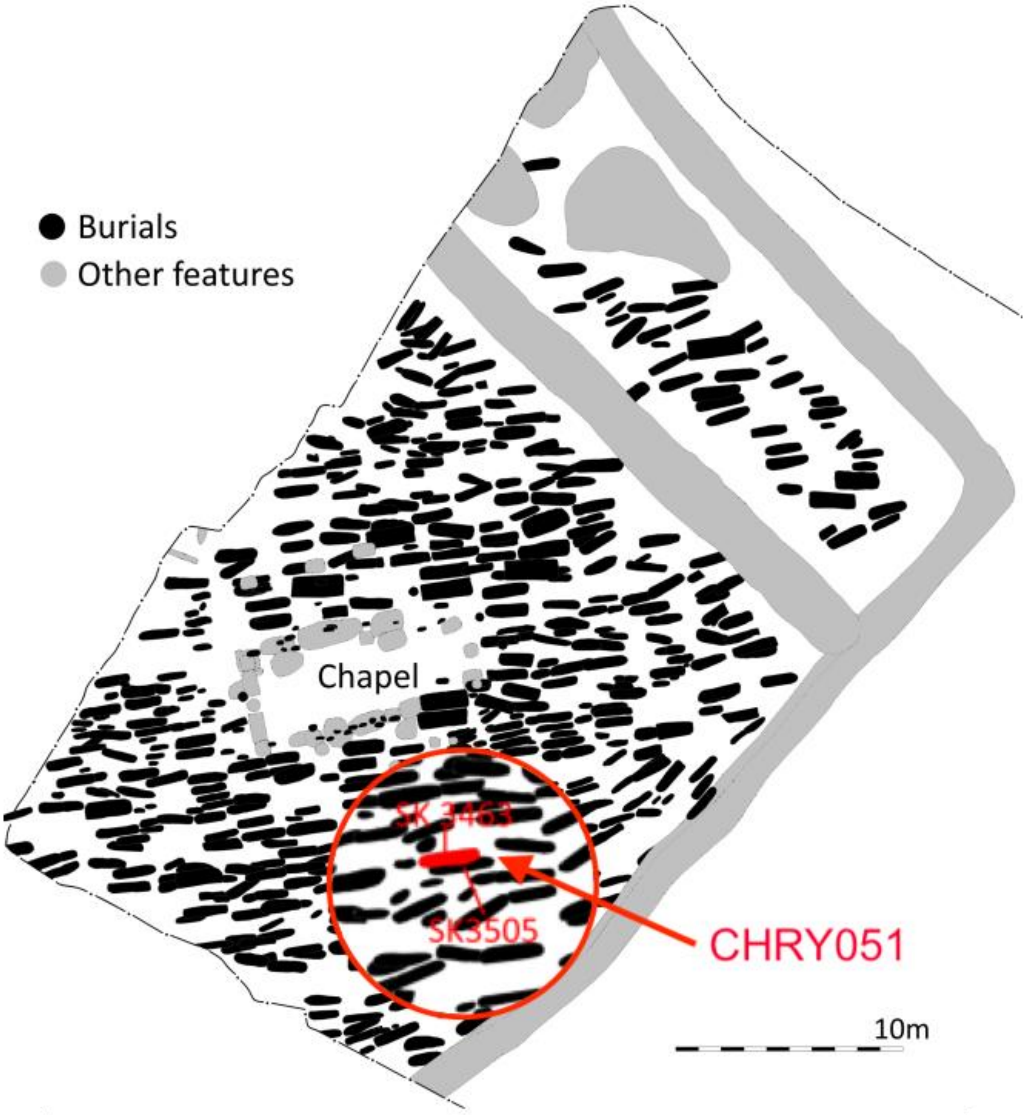

Fig S10. Location of SK3463 (CHRY051; individual with traces of *M. leprae*) within the Cherry Hinton Site. SK3463 intersects a previous burial SK3505, possibly causing the contamination of the sediments with DNA from previous depositions.

SI1.6 St John's Divinity School / Hospital of St John, Cambridge

See also descriptions in (Hui et al. 2024; Inskip et al. 2023; Guellil, van Dorp, et al. 2022; Cessford et al. 2021; Cessford 2015).

The Hospital of St John the Evangelist was established around 1195(Cessford 2015; Inskip et al. 2023). For much of its existence, it comprised a dormitory building where the inmates resided, along with a chapel and various service outbuildings (Cessford 2015; Inskip et al. 2023). During the fifteenth century, the hospital experienced a decline, ultimately leading to the founding of St John's College in 1511 (Cessford 2015; Inskip et al. 2023). The hospital's cemetery served as a burial ground for inmates and, at times, townspeople, whereas clerical staff and affluent benefactors were interred in and around the chapel (Cessford 2015; Robb et al. 2019; Faber, Napran, and T. E. Faber Estate 2006). Excavations conducted by the Cambridge Archaeological Unit in 2010–11 uncovered nearly 400 complete and partial skeletons from the cemetery (Cessford 2015). The burials primarily consist of extended supine inhumations, oriented west–east, in simple earth-cut graves, wrapped in shrouds, and without any grave goods (Cessford 2015). We sampled one vertebra and one rib respectively from individuals JDS123 and JDS157 (Fig. S11-S12).

JDS123 (Feature 737, Early-Mid phase, skeleton 1317), was an immature individual, and it was not possible to determine the sex archaeologically. The skeleton was more than 75% complete and was found with pottery dating to the 13th century. A vertebrae was retrieved in a previous study (Hui et al. 2024), and sediments attached to it were sampled for this study.

JDS157 (feature 457, Undetermined phase, skeleton 4093), an adult male, was found with less than 50% of the skeleton complete, prone and east-west aligned. In the grave there was pottery

dating from the 14th century. The C14 results date the skeleton to 693±30 years BP. A rib and sediments adhered to its surface were retrieved in this study.

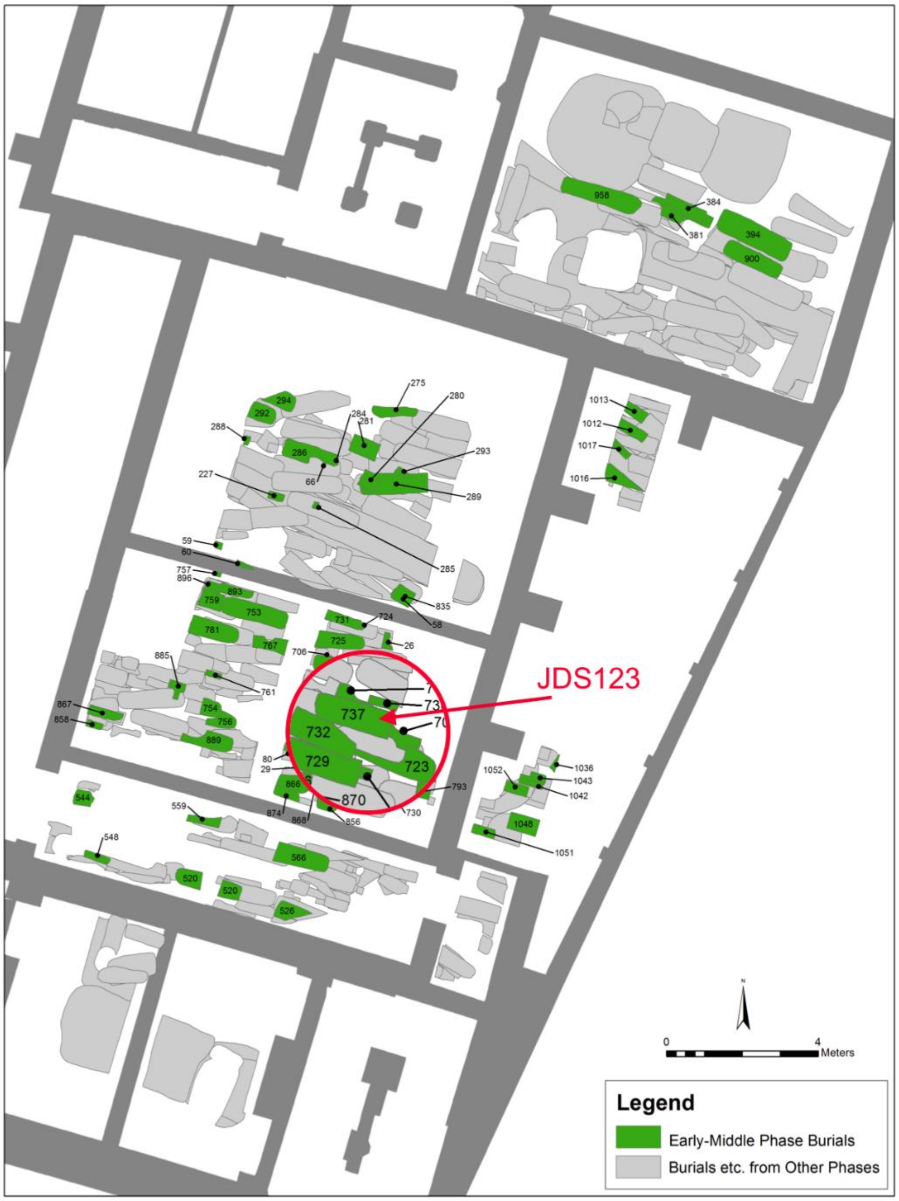

Fig S11. Distribution of Early-Middle phase burials in St. John's. The sample studied in the paper is indicated with a red arrow and a circle. Modified from Cessford 2015 (Cessford 2015).

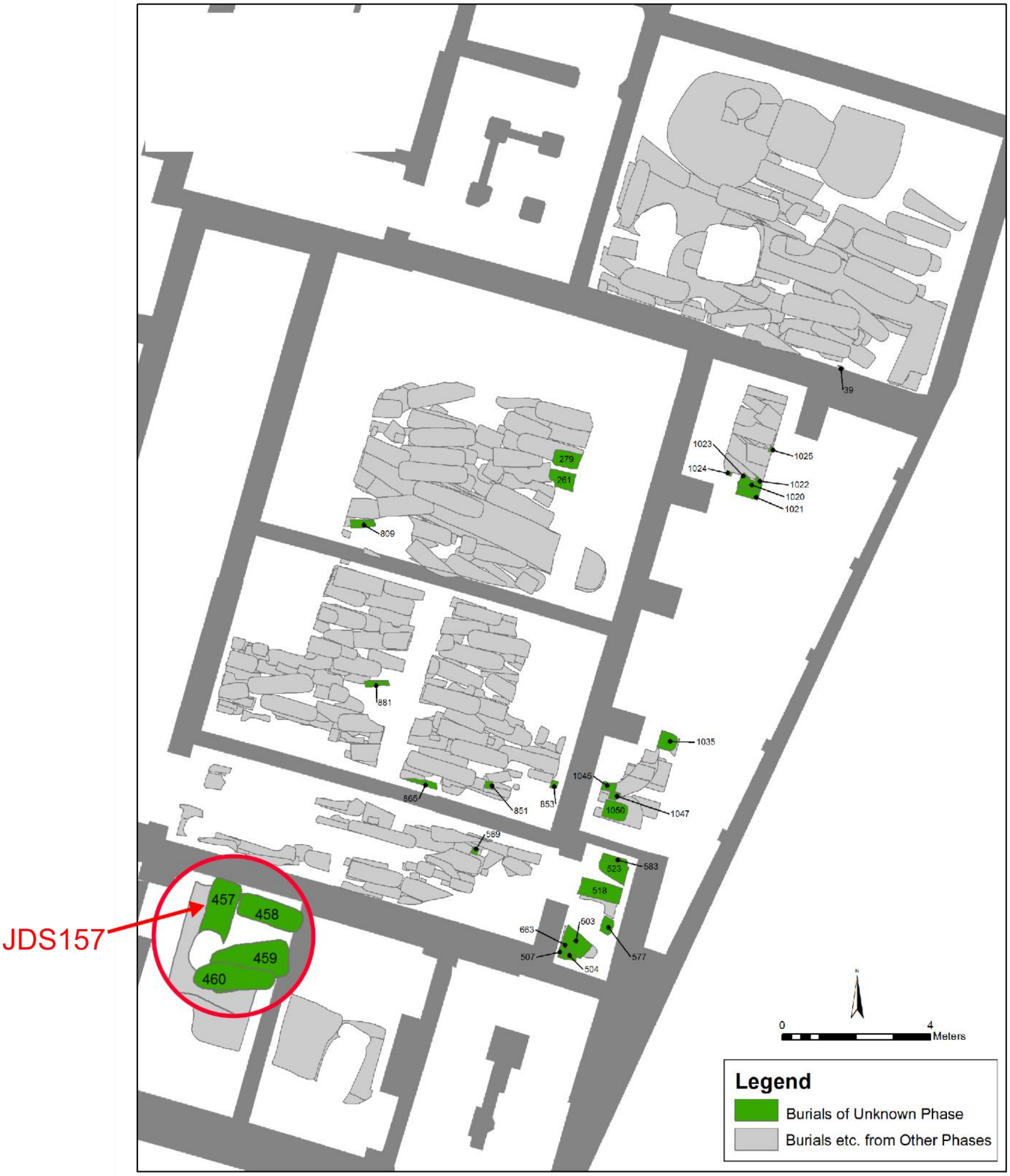

Fig S12. Distribution of Unknown phase burials in St. John's. The sample studied in the paper is indicated with a red arrow and a circle. Modified from Cessford 2015 (Cessford 2015).

SI1.7 New Museums Site / Augustinian Friary, Cambridge

See also descriptions in (Hui et al. 2024; Cessford et al. 2021; Cessford and Neil 2022; Cessford 2015; Cessford et al. 2022, 2023; Cessford 2017).

The Augustinian Friary was established in Cambridge between 1279 and 1289. The convent grew rapidly, becoming a studium generale or national house of studies in 1318 and numbering 70 friars in 1328 (Cessford 2015). It continued, until its dissolution in 1538, as one of the most important Augustinian convents in England and one of the largest institutions in Cambridge. The skeletons reported in this study were recovered during pre-development archaeological excavations of the site undertaken by the Cambridge Archaeological Unit in 2016-2017 (Cessford 2017). The samples were dated using archaeological and isotopic techniques. The skeletons were initially studied by the Cambridge Archaeological Unit and re-examined by the After the Plague project. Human remains were recovered from three sites in the convent: an early cemetery and the later chapter house and cloister (Hui et al. 2024). The individuals analysed in this study come from the Friary Sub-phase 2.1 cemetery (Figure S13). It appears that the convent acquired the burial rights of the individuals in 1290. Based on radiocarbon dating, stratigraphy, artefact typology and architectural criteria, the burials in the chapter house date to around 1275/89–1320/40 (Cessford and Neil 2022). Some of the burials were accompanied by simple buckles placed near the pelvis, indicating that the bodies were buried clothed, with surviving evidence of associated leather girdles and some evidence of textiles (Cessford et al. 2022).

The presence of animal assemblages is considerably higher in the pre-friary phase, the Friary Sub-phase 2.1 contains remains of domestic animals such as cow, sheep/goat, dog, horse, pig and *galliformes.* Later post-friary phase contains a wider range of species in terms of skeletal remains,

but much less occurrence of the remains. No evidence of rodent remains was found (Cessford 2017).

NMS022 (F.328) was most probably a male, characterised as a sub-adult, but without a precise age estimation. It was 170 cm in height and did not present clear evidence of pathology. The preservation of the remains was good. Petrous bone was sampled for a population genetic study (Hui et al. 2024), and sediment for this study.

NMS031 (F.333) was an adult male of approximately 34 years old, with good skeletal preservation. The individual was approximately 170 cm in height. This particular individual presented extense signs of pathology, including: minor bilateral cribra orbitalia, greater expression in right cribra, cranii over supraorbital ridge, notably thick diploe, osteo-artrhitis (minor spondylophytes on anterior borders of T3–T11 and L2–L5 bodies greatest expression between T5–T6), enthesophyte formations on anterior table of right patella, spur on the medial posterior metaphysis of the right fibula, and minor longitudinal cracking and mottling. This individual had an iron buckle. The petrous bone of this individual was also sampled in Hui et al. 2024, while sediments attached to the aforementioned bone were sampled for this study.

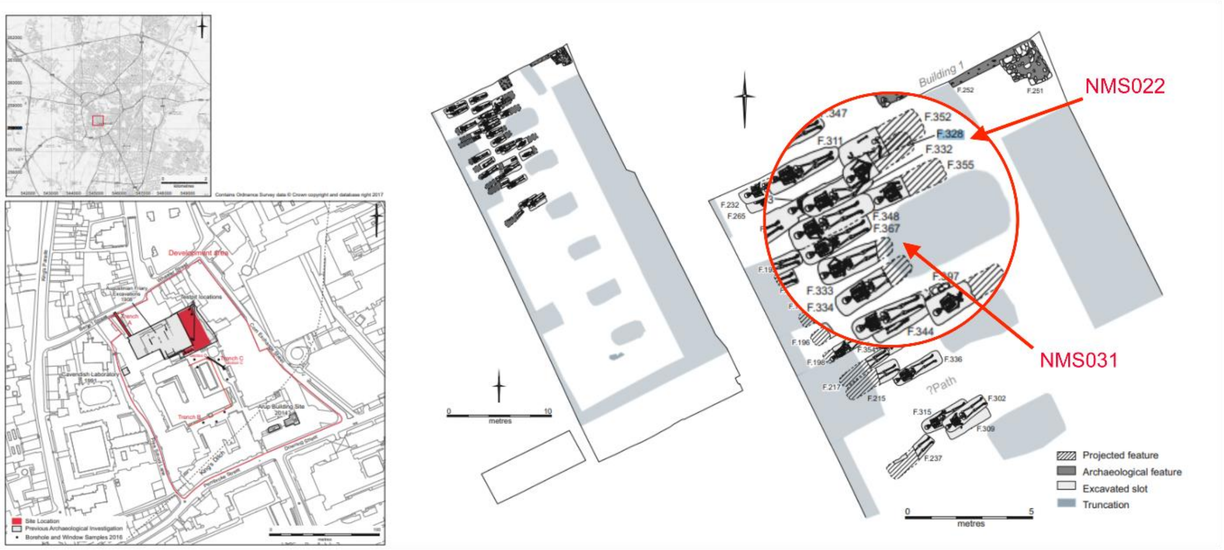

Fig S13. Location of New Museums Site within the City of Cambridge. Zoom in into the Friary Sub-phase 2.1 cemetery, with individuals NMS022 and NMS031 (F.328 and F.333 respectively) highlighted in red. Modified from Cessford 2017 (Cessford 2017).

Supplementary Methods

SM1 Metagenomic Microbial analysis

SM1.1 Reference dataset creation

We downloaded raw fastq sequences from ENA (Velsko et al. 2019; Rampelli et al. 2015; Oh et al. 2016; Mukherjee et al. 2023; Human Microbiome Project Consortium 2012; Stewart et al. 2019; Su et al. 2022; Tisza and Buck 2021; Lloyd-Price et al. 2017; Bissett et al. 2016) (Supplementary Table S29). Adapter sequences were trimmed and read pairs were merged using AdapterRemoval2, with a minimum length of 30, minimum overlap of 11 and minimum sequencing quality of 20. Low complexity sequences using prinseq and a dust value of 7 (Schmieder and Edwards 2011) and duplicated reads were collapsed using bbmap (Bushnell 2015). Deduplicated and high-quality sequences were mapped against the Human Reference Genome 37 using BWA mem (Li 2013). Human free sequences were then classified against a Microbial Database (MicrobialDB as for 16/08/2020) using KrakenUniq (Breitwieser, Baker, and Salzberg 2018; Pockrandt, Zimin, and Salzberg 2022). Raw KrakenUniq were filtered using the E-score formula described by Borry and Guellil ("A New E-Score for KrakenUniq" 2022; Guellil et al. 2018; Guellil, Keller, et al. 2022). We accepted a minimum E-score of 7 and 200 sequences as minimum values to consider a hit as true down to the taxonomic level of species.

After the initial bioinformatic processing of the individual samples, we proceeded to merge all the filtered reports in one unique dataset. To achieve this, we use a custom python script, discarding all species that were represented with under the 0.02% of the total sequences of the whole dataset.

To test the behaviour of this reference dataset, we used Sourcetracker2 strategy *leave-one-out* (loo) (Knights et al. 2011). In brief, this feature iteratively compares each different sample in the dataset

against all the samples (Suppl Figure S37). We further validate these results by performing a hierarchical cluster analysis of the Bray Curtis distances of the samples' microbial abundances using R package *cluster ("'Finding Groups in Data': Cluster Analysis Extended Rousseeuw et Al. [R Package Cluster Version 2.1.6]" 2023)* (Suppl Figure S38). We then proceed to discard the following samples *due* to their low levels of similitude to other samples in their clusters: *skin25, CowRumen-10678_0001, CowRumen-10678_0006, CowRumen-10678_0007,* and *CowRumen-10678_0013.*

Once the source dataset was properly delimited, we generated a set of source-specific species. Those were characterised using Sourcetracker2 option "*per_sink_feature_assignments*" and were estimated to human gut (HuGt=49; ruralGut and urbanGut), ruminant gut (RmGt=10; CowRumen and Sheep Rumen), skin (Skin=45), oral (Oral=64; ModernCalculus, Subplaque and Supplaque), and soil (Soil=14; Soil and FarmlandSoil) (Supplementary Table S15). Although for the most part, species are unique to a source, some level of species overlap is found between human gut and rumen (HuRm=10), soil and skin (SlSk=9), and oral and skin (OrSk=12) (Supplementary Figure S39).

SM1.2 GC content analysis

Analysis of the GC content of each source reveals a weak tendency for GC bias in short human gut and soil microbial sequences (HuGt, HuRm, Soil, SlSk $p < 0.01$), but not in oral, ruminant gut, or skin microbiomes (RmGt $p = 0.375$; Oral $p = 0.629$; OrSk $p = 0.316$; Skin $p = 0.176$) (Supplementary Figure S40). Despite this, it is important to remark bias introduced due to low coverage of the samples.

SM2 Eukaryote DNA Screening

SM2.1 KrakenUniq dataset creation

We downloaded all refseq sequences corresponding to plastids and mitochondria as of 02/11/2022. Additionally, we added the whole Human Reference Genome 38 and the contaminants database using KrakenUniq built-in function "*--download-library*". We then proceed to build the KrakenUniq database using default settings (*--kmer-len 35, --minimizer-len 31*). This operation was run using 500GB of RAM memory and 32 cpus.

In addition to that, we also build a set of reference genomes for each different genus and species. To do that we selected all the representative genomes for each different species according to NCBI. We then created the individual reference genome for the species and proceeded to obtain taxonomic information using taxonomy_ranks available in ETE toolkit (Meng et al. 2019). We then concatenated all species genomes inside a genus into a fasta and then converted into a reference.

SM2.2 Screening

We ran the preprocessed data (see Methods) against the aforementioned custom Organelle KrakenUniq database (Supplementary Figure S25). In order to validate the hits against animal mitochondria we used the E-score formula described by Guellil and Borry on the raw KrakenUniq reports. In this case, we used an E-score of 7 at a genus level and a minimum of 3 reads per hit. The reasoning behind this is that three reads match a read length of 50bp, representing around 1% of the mitochondrial genome for an animal. We used sheep bone samples Z1460, Z1461, Z1463, and Z1464 as controls (Supplementary Figure S28).

Once target species were selected, we created a reference by concatenating all species found inside the target genus into a unique reference. This step was purely done to speed up downstream analysis. We mapped the preprocessed FASTQ against this multi-genus reference genome with the common ancient DNA (Schubert et al. 2012) mapping settings in BWA *backtrack (Li and Durbin 2009)*; edit distance of 0.01 (-e 0.01), gap open penalty of 2 (-o 2) and seeding disabled (-l 10,000). All reads with a mapping quality of 0 or higher were converted back into FASTQ using the command "*samtools fastq"(Danecek et al. 2021),* keeping the original qualities found in the read. Afterwards, we started a set of parallel mappings, using those filtered FASTQ, and all the different genus references. We reclassified mapping reads according to lowest Edit Distance to each genus, discarding shared reads with equal edit distance in the process, using a custom-build python script. This was followed by a step of BAM to FASTQ reconversion by genus, and a second step of mapping, this time for individual species inside the analysed genus. Reads were reclassified by Edit Distance, obtaining individual BAMs for each different species. Basic statistics were performed for those BAMs using Qualimap2 (Okonechnikov, Conesa, and García-Alcalde 2016). We then selected the best candidates for species by the number of mapped reads, and in cases of an equal number of mapped sequences, according to average edit distance. Finally, we mapped the original pre-processed FASTQ against each different individual target reference genome and reclassified the sequences (Supplementary Figure S26. We generated basic mapping statistics with Qualimap2 and aDNA damage patterns with MapDamage2 (Jónsson et al. 2013). We also calculated the -Δ statistic (differential of edit distance) (Herbig et al. 2016) (Supplementary Figure S29). We assumed a species was present if at least 3 reads mapped to the species. Whole genome references were then created for those species to map against.

The reasoning behind this initial screening and iterative steps of mapping and reclassification is to overcome the computational limitations of the cluster. Metagenomic classification software such as Kraken2 or Centrifuge, despite being extremely fast and relatively accurate, do not allow for proper result validation (Wood, Lu, and Langmead 2019; Kim et al. 2016). KrakenUniq alone could be a good candidate (Breitwieser, Baker, and Salzberg 2018), unfortunately even with new memory saving settings present in the latest release of the software (Pockrandt, Zimin, and Salzberg 2022), building a database including all whole genomes from Eukaryotes requires a substantial amount of memory. Finally, MALT needs a prohibitive amount of RAM memory to run (Herbig et al. 2016). Following the pipeline above we manage to run our samples (10-20 million reads), in less than 2 hours using only 20GB of RAM memory and 8 cpus. All directories and files associated with this pipeline including KrakenUniq database, reference genomes and metadata files have an approximate disk space of less than 3GB.

SM2.3 Whole genome mapping and quality control

We downloaded whole genomes for the target species and created references from them. We mapped using *BWA backtrack* as previously specified and reclassified the resultant mapped reads according to their edit distance, in order to avoid spurious mappings between evolutionary close organisms (Supplementary Figure S36). To finalise the authentication process, we validated the resultant sequences according to the presence of aDNA deamination patterns, read length distribution, edit distance distribution decay, and normalised coverage distribution across the genome. (See Figure S26).

SM3 Proteomic analysis with novor.cloud

We used *novor.cloud*, a cloud version of the mass spectrometry data analysis software Novor (Ma 2015). Data was analysed using the same databases used for the final analysis with pFind. (SwissProt and cRAP). The search includes the set of modifications used in pFind search; fixed modifications (carbamidomethylation of cysteine), and variable PTMs (proline hydroxylation, glutamine and asparagine deamidation, methionine oxidation, and pyroglutamate formation from glutamine and glutamic acid). As with pFind, the precursor mass tolerance was set to 15 ppm, fragment mass tolerance to 0.02 Da, and the false-discovery rate of peptide spectrum matches equal ≤1.0%. Finally, and as in pFind analysis, protein hits were considered valid if 2 or more peptides for that protein were present.

Using this approach, 11,559 were recovered across all samples, accounting for 501 proteins (Figure S15). From those, an average of 57% of peptides across all samples are of human origin. In the case of DUX012B, a sample with a considerable number of peptides assigned to bacteria by pFind (Figure S16), novor.cloud displays similar results, with 87% of peptides in the sample being considered bacterial in origin. This is also the case for NMS022B, which is also the one with a higher diversity of peptides and proteins using this software (2,363 peptides and 96 proteins identified), most of them corresponding to rodents (R. norvegicus=1,436; M. musculus=295).

Supplementary Figures

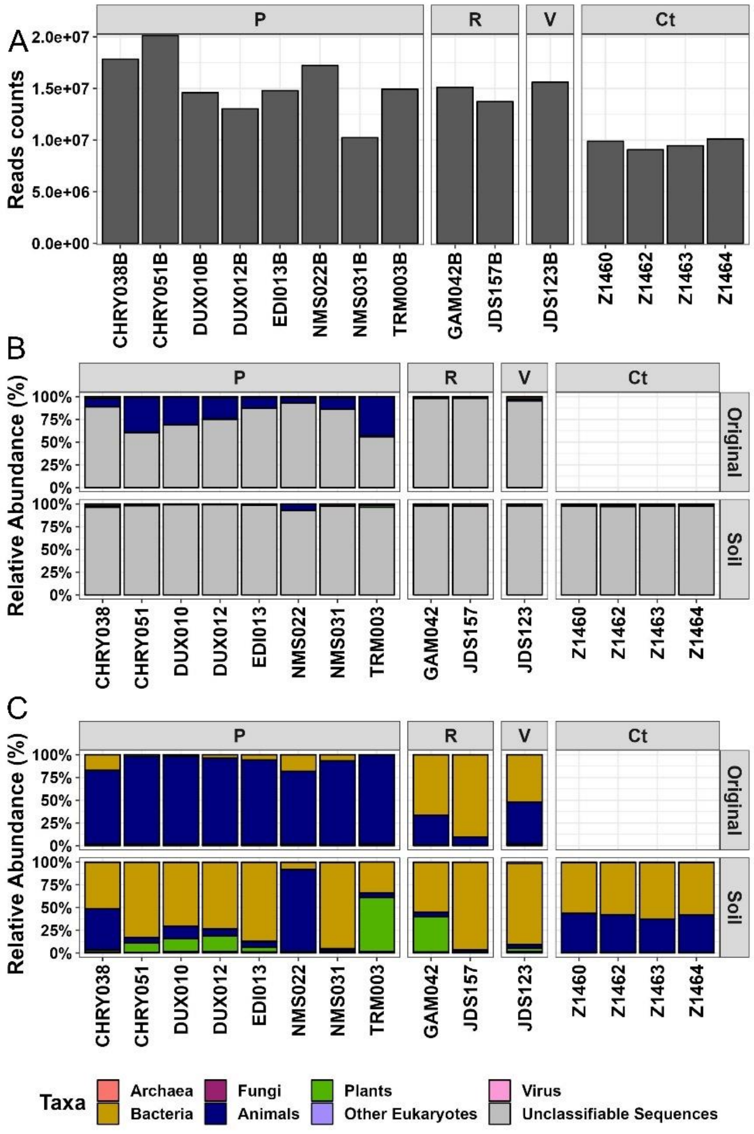

Figure S14. Sequences' abundances. Barplot displaying the number of generated sequences per sample (A). Stacked plot of the relative abundance of sequences assigned by Kraken2 to the different domains of life (Archaea, Bacteria, Eukaryota [Fungi, Animals, Plants, Other], Virus), with (B) and without (C) taking into account unclassifiable sequences for both Sediments and Original Skeletal element. Samples are grouped by their sampling origin (P = Petrous Bone Soil, R = Rib Soil, V = Vertebrae Soil, Ct = Control). Z1460, Z1462, Z1463, and Z1464 are positive controls for Eukaryotic sequences' screening (Ancient sheep bones).

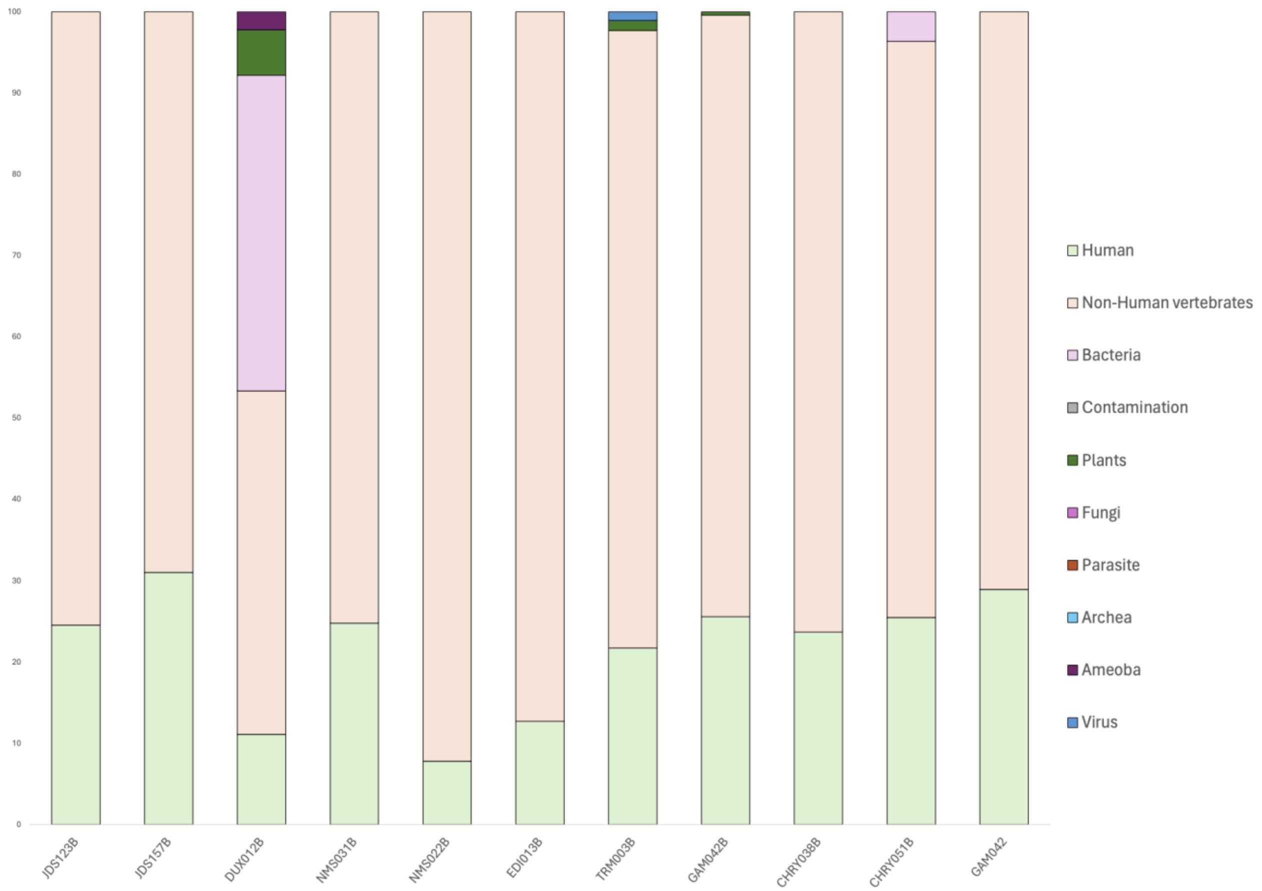

Figure S15. Relative proportions of peptide origin. Stacked bar plot showed the relative abundance for peptide origin based on pFind output.

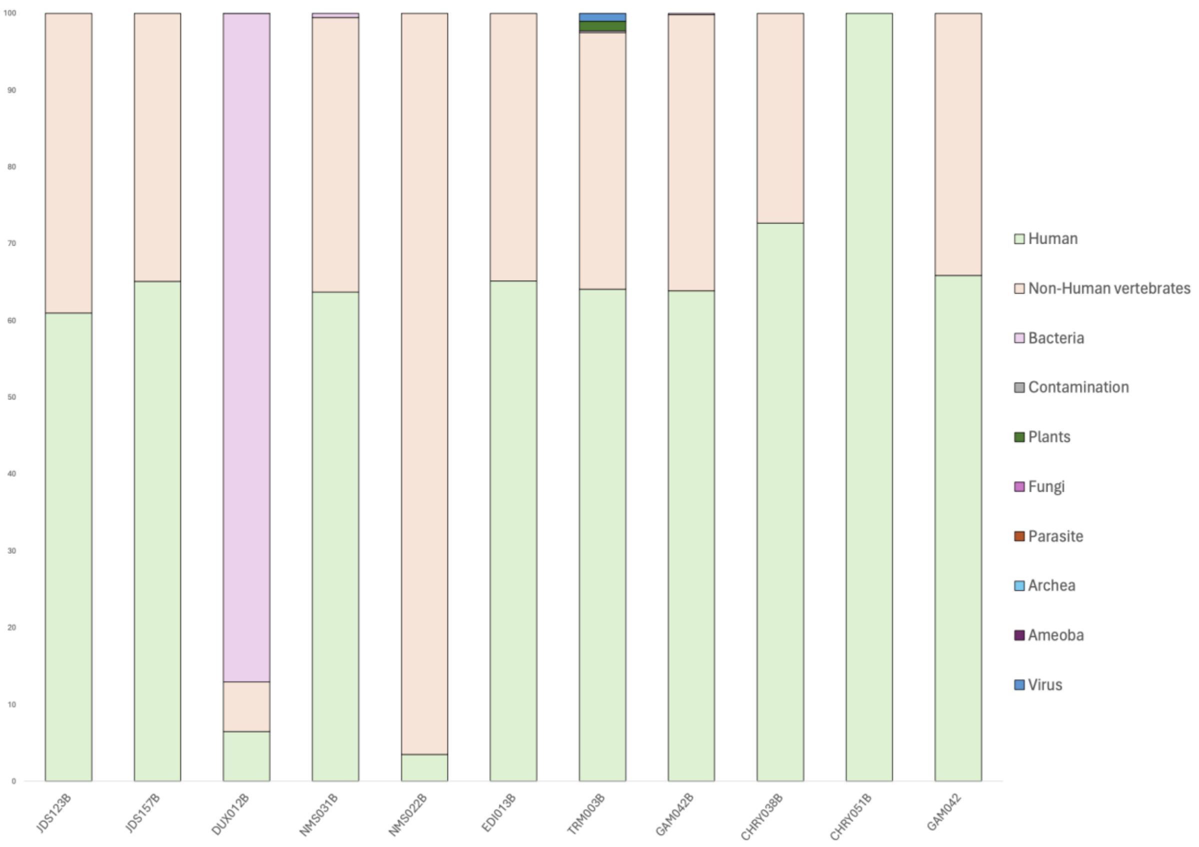

Figure S16. Relative proportions of peptide origin. Stacked bar plot showed the relative abundance for peptide origin based on novor.cloud output.

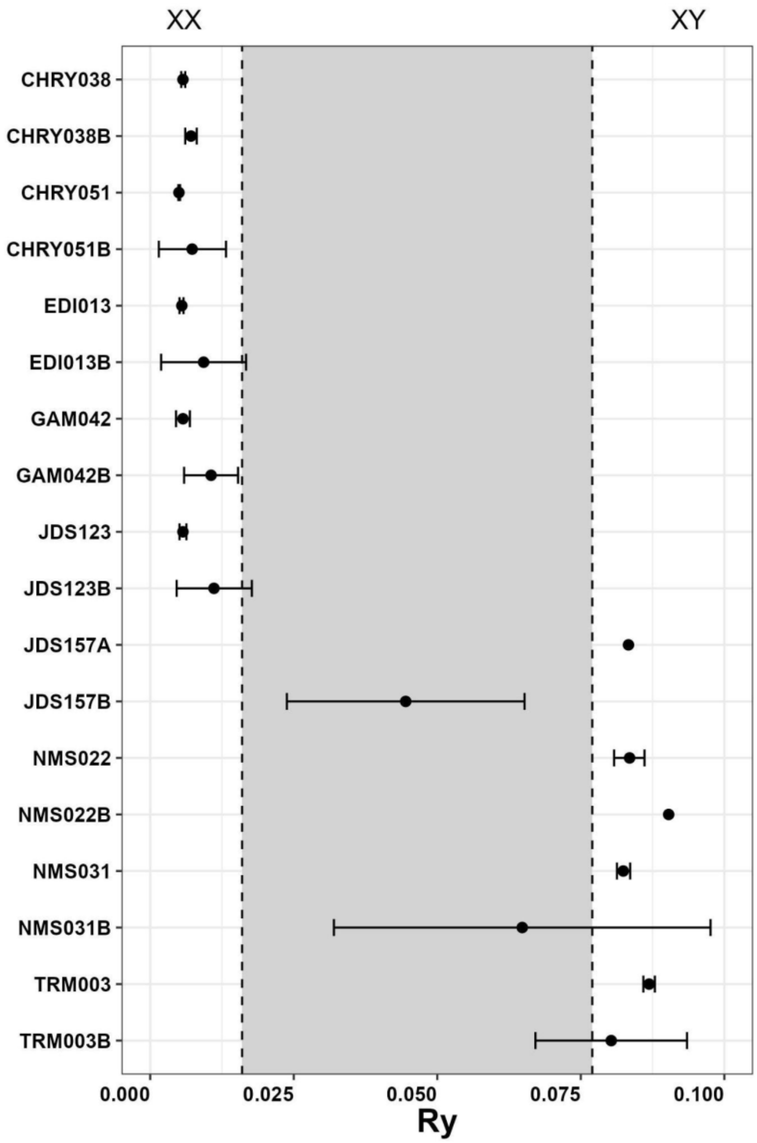

Figure S17. Sex determination for human sequences in each sample. Sample ID without letter B is the original skeletal sample as published in (Hui et al. 2024, Keller et al. 2019, Scheib et al. 2019, or Scheib et al. *in preparation*).

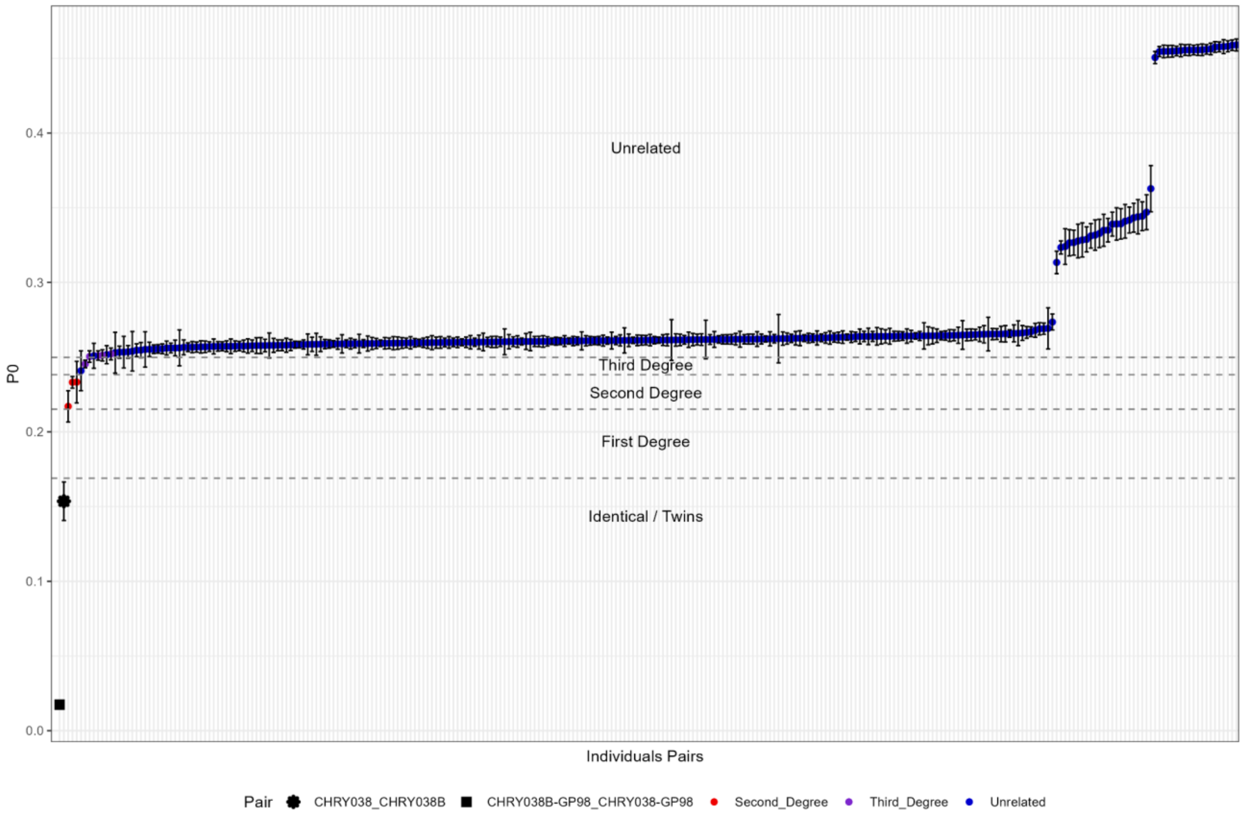

Figure S18. Degree of relatedness determined by READv2 between CHRY038, CHRY038B, and other published contemporary individuals from the Cherry Hinton site. CHRY038 and CHRY038B are designed as the same individual/identical twins before (black asterisk) and after (black square) imputation.

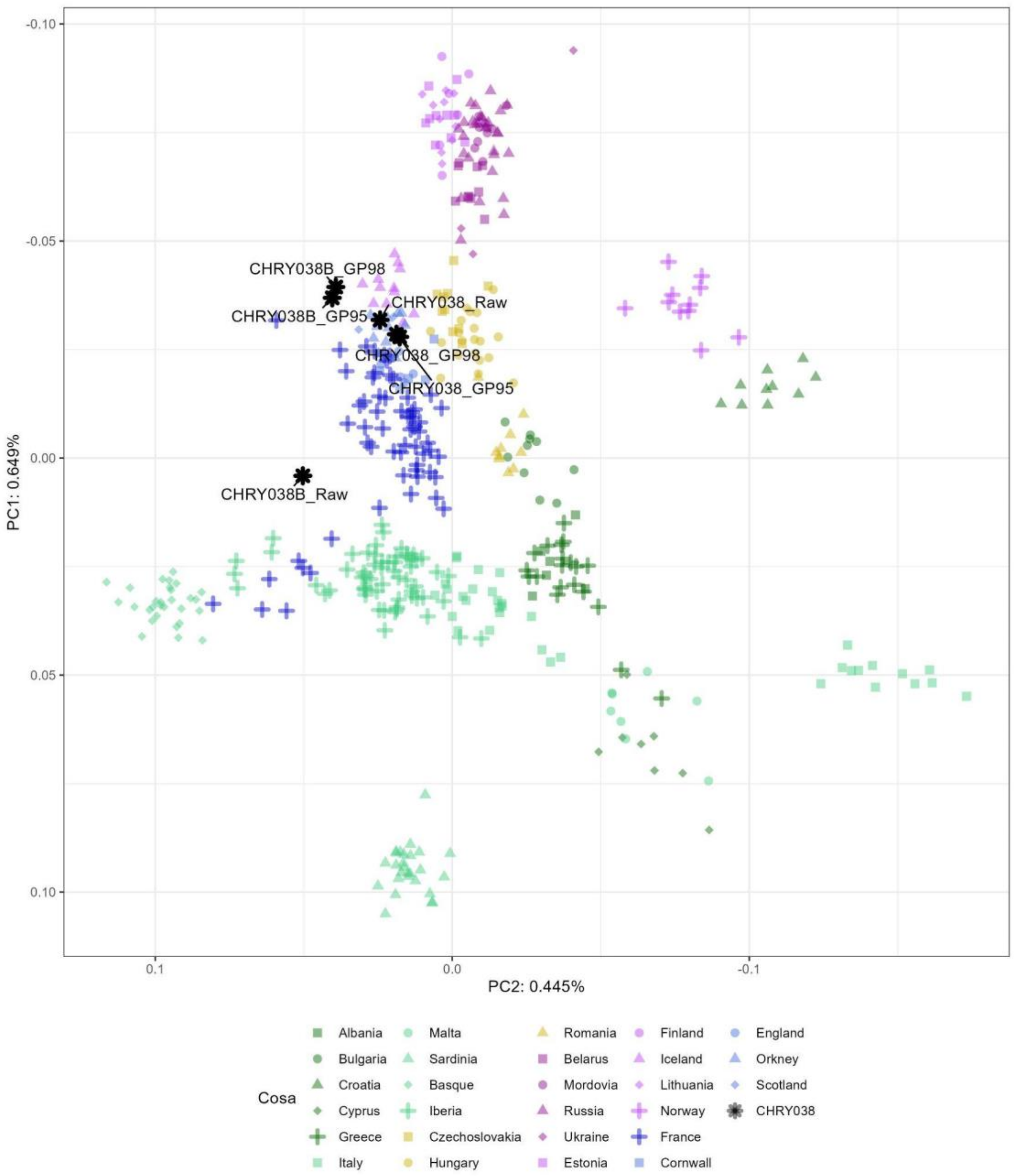

Figure S19. CHRY038 human sequences PCA, including both sequences retrieved from the original skeletal element (CHRY038) and soil (CHRY038B), with different levels of imputation; No Imputation (Raw), Genotype Probability of 95 (GP95), and Genotype Probability of 98 (GP98).\

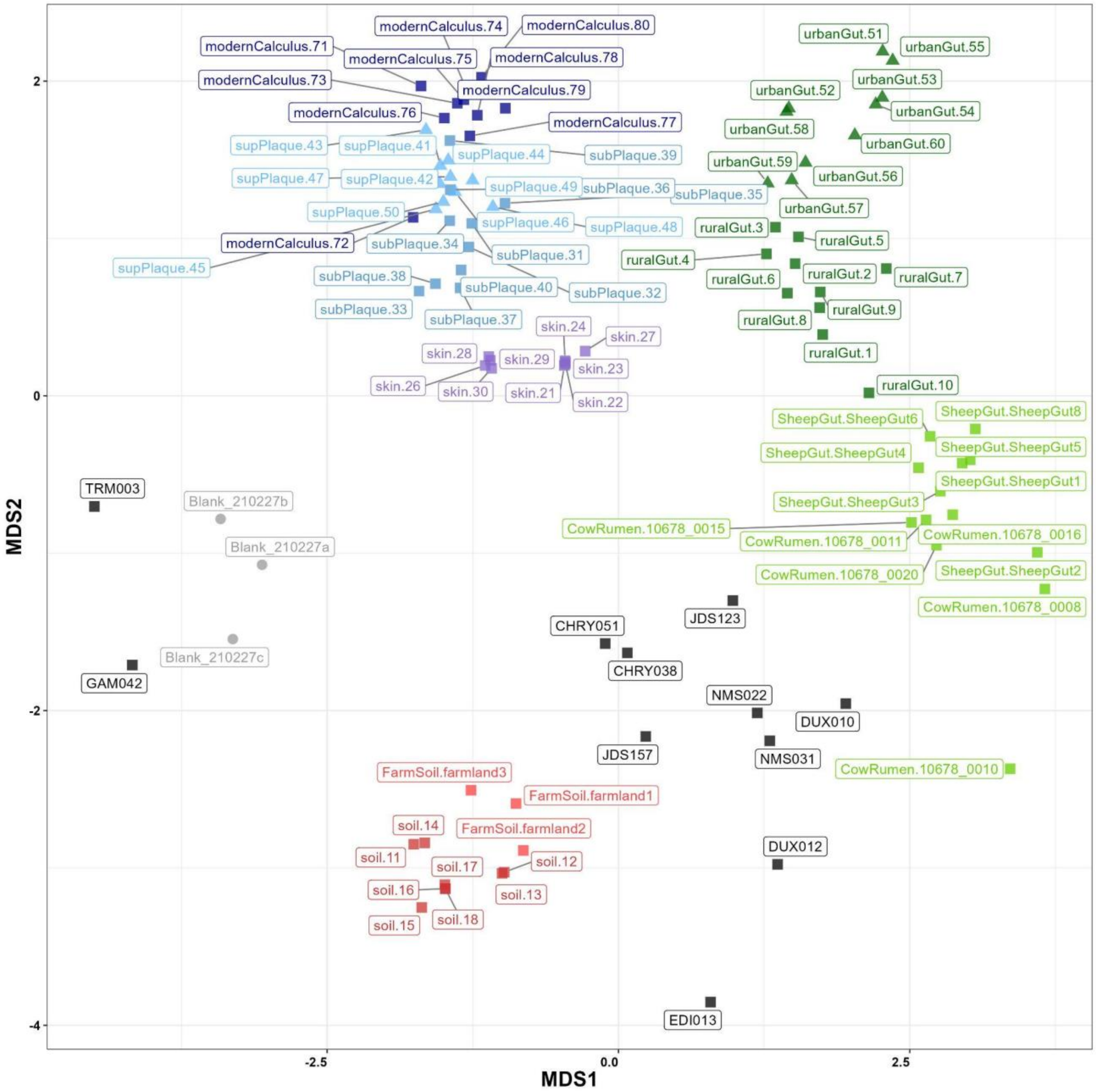

Figure S20. Non-metric dimensional scaling plot using Bray-Curtis distances of analysed ancient soil samples and a set of modern references metagenomic profiles obtained with KrakenUniq. Modern microbiome references include Extraction Blanks (Blank), soil (Soil), farmland soil (FarmSoil), human non-industrialised gut microbiome (ruralGut), human industrialised gut microbiome (urbanGut), ruminant gut microbiome (CowRumen and SheepRumen), human skin microbiome (skin), human calculus (modernCalculus), human supragingival plaque (supPlaque) and human subgingival plaque (subPlaque).

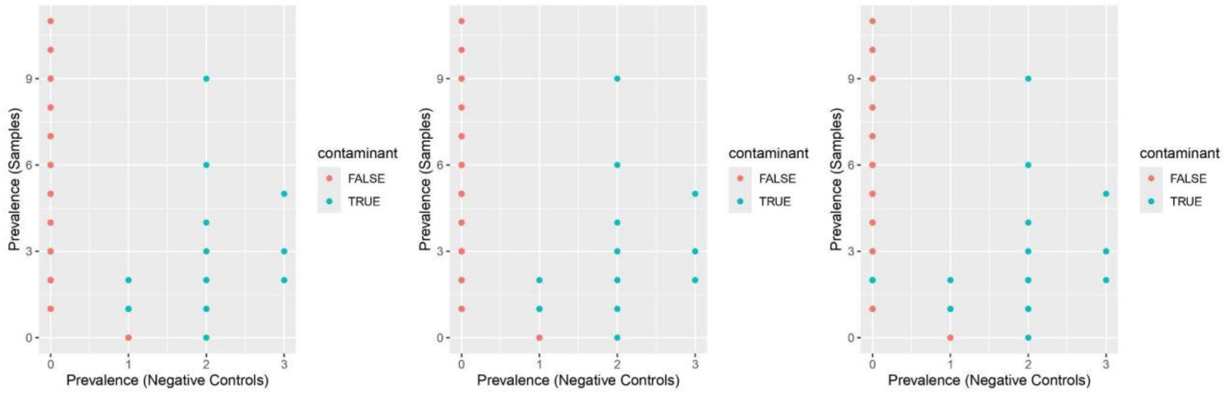

Figure S21. Contamination estimates as found by Decontam. Different thresholds were tested: 0.5 (left), 0.6 (middle) and 0.7 (right). We used the astringent value of 0.7.

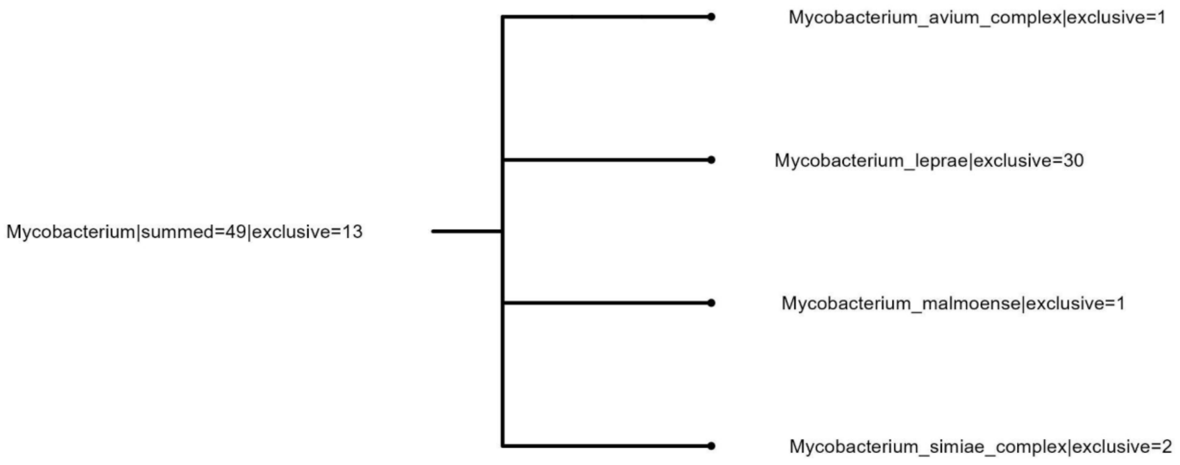

Figure S22. Blastn results of reads assigned by KrakenUniq (51) to *Mycobacterium leprae* in sample CHRY051B. From the initial number of sequences, 30 are exclusive of *M. leprae,* while 13 are common to the *Mycobacterium genus.*

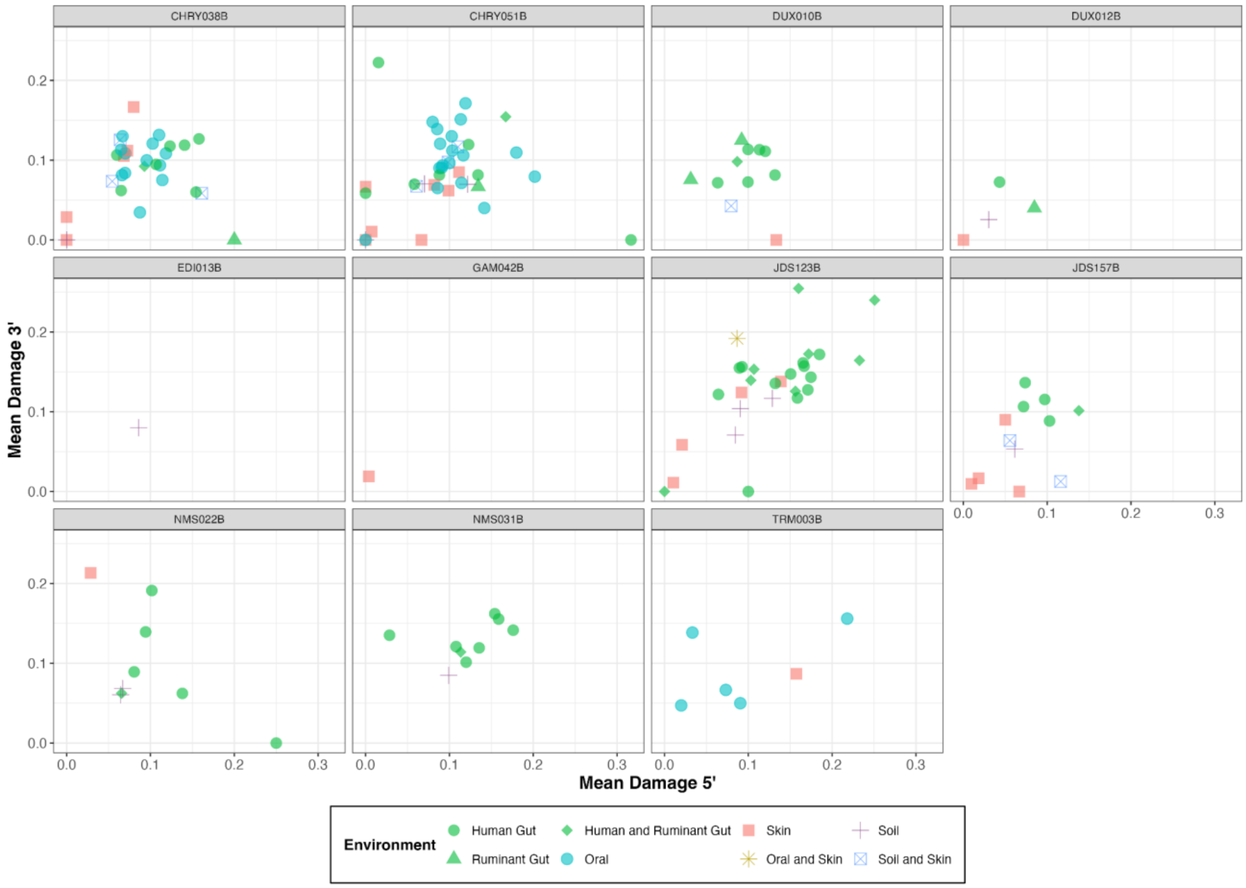

Figure S23. Average aDNA damage present at the last 5 bases of sequences mapped against bacterial genomes characteristic of sources. The 5' and 3' ends' damage is displayed in the X and Y axis respectively.

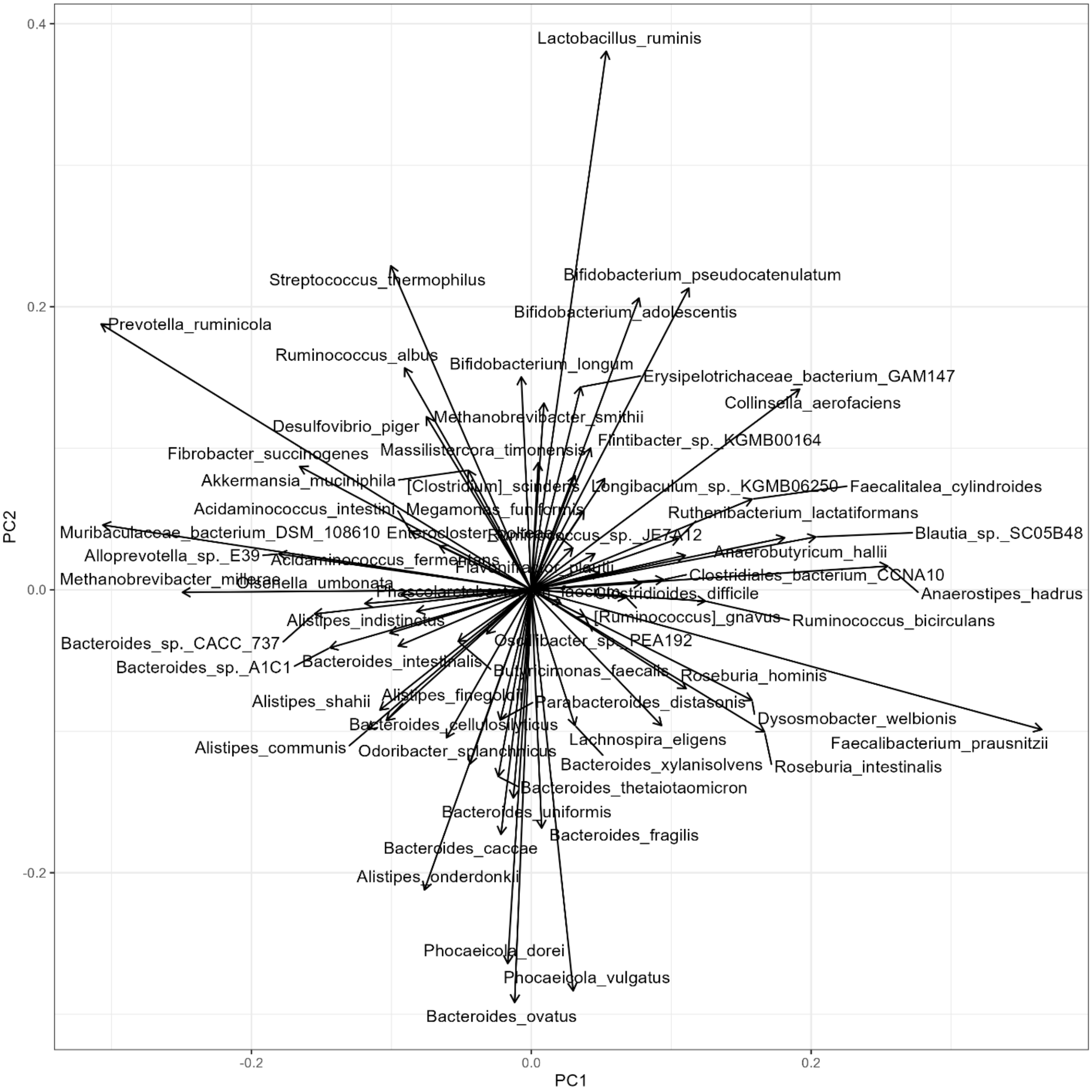

Figure S24. PCA loadings for the gut microbiome PCA with the Human and Ruminant Gut microbial species assigned by sourctracker2.

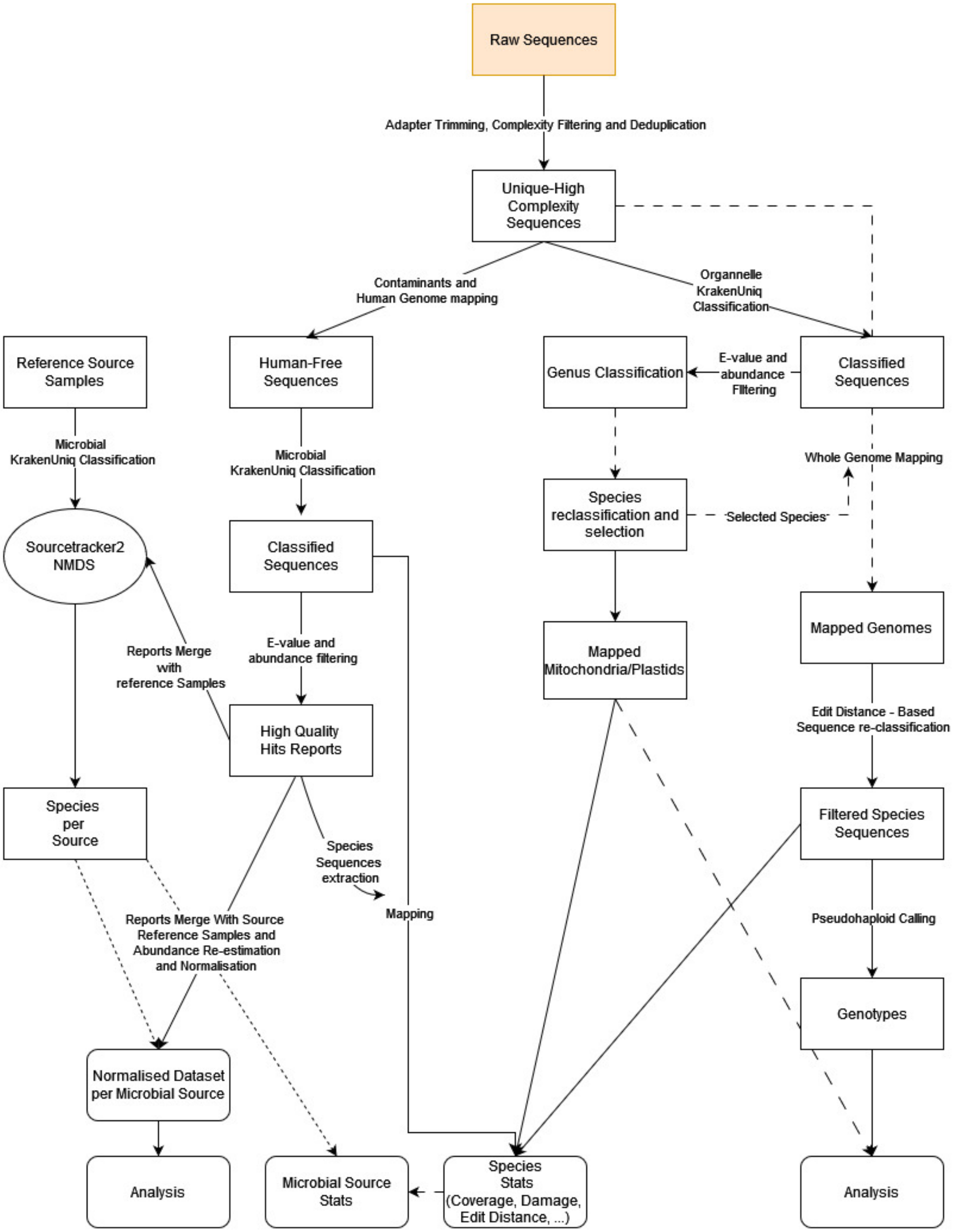

Figure S25. Diagram of custom pipeline developed for this study.

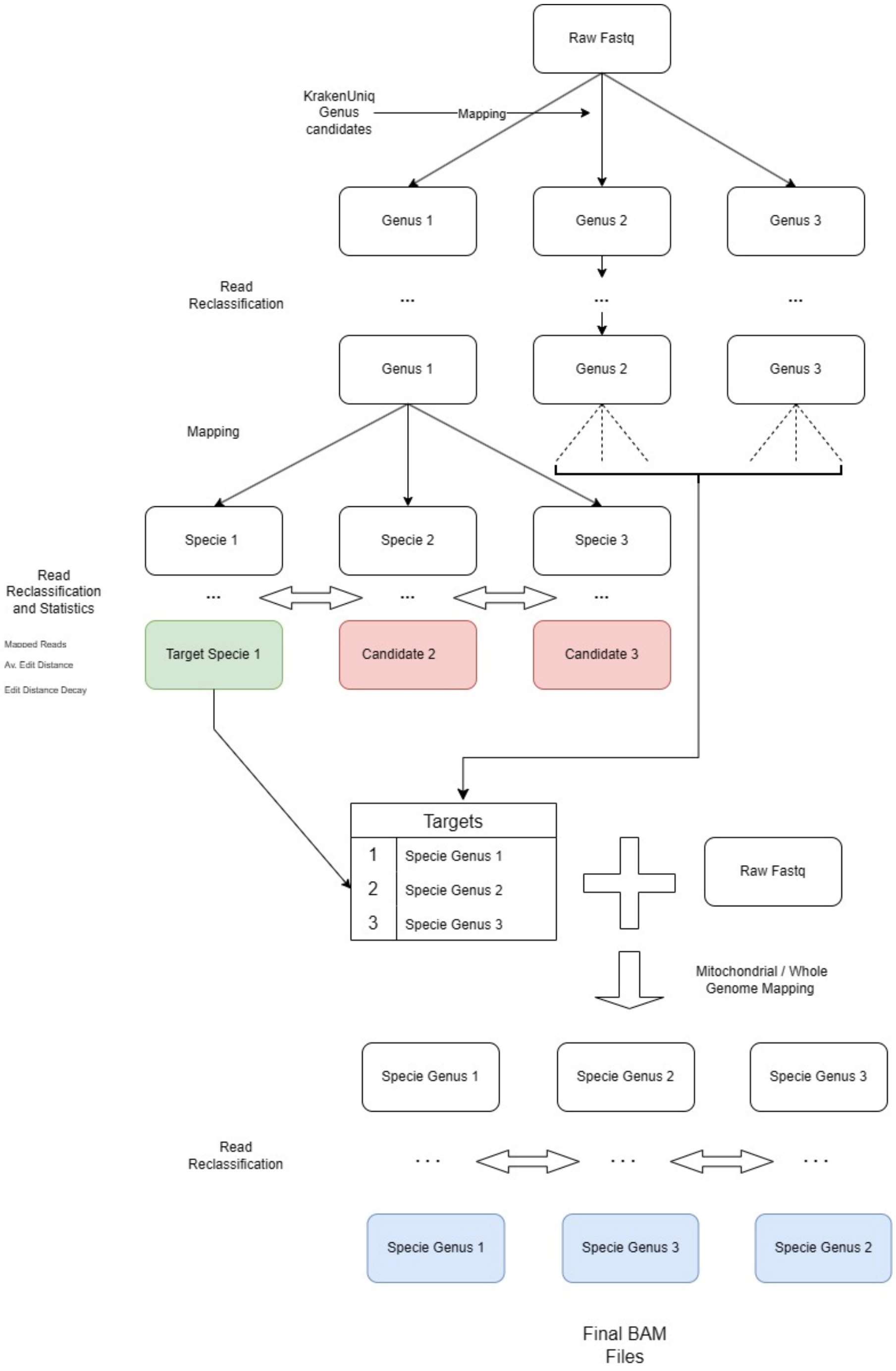

Figure S26. Diagram of the approximation used to identify Eukaryotic species.

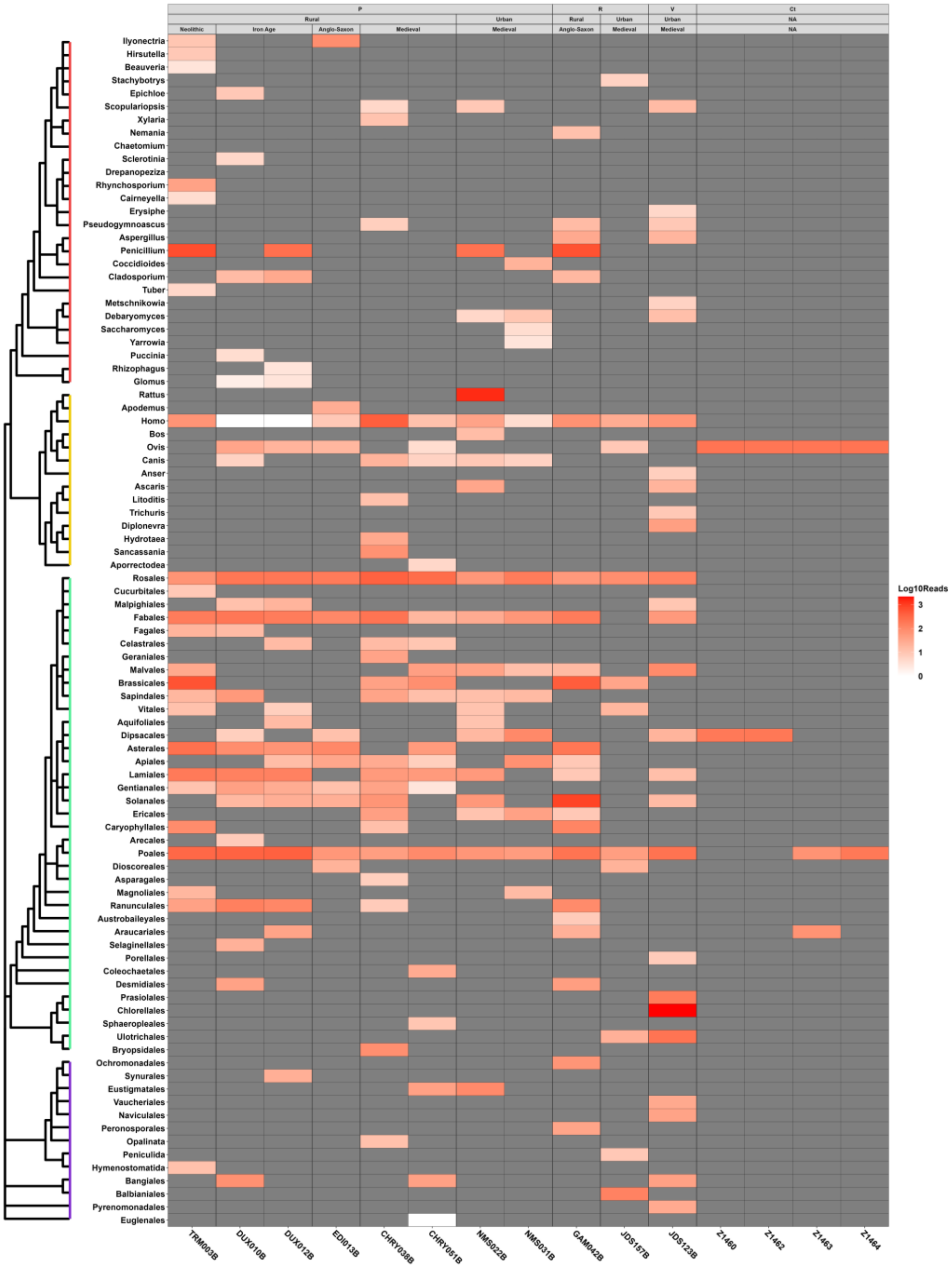

Figure S27. Heatmap of Eukaryotic Organelle DNA found in the samples (including controls). Vertical axis is ordered by the taxonomic location of detected species, with colours denoting the clades of interest (Red=Fungi, Yellow=Animals, Green=Viridiplantae, Purple=Protist). Fungi and Animals are classified down to genus level, while Plants and Protist to order level. Samples are grouped by their sampling origin (P = Petrous Bone Soil, R = Rib Soil, V = Vertebrae Soil, Ct = Control), context of site (Rural vs Urban), and site datation (Neolithic, Iron Age, Anglo-Saxon and Medieval). Cells are filled with read numbers normalised to a log10 scale, with higher numbers displaying a darker red. Z1460, Z1462, Z1463, and Z1464 are positive controls.

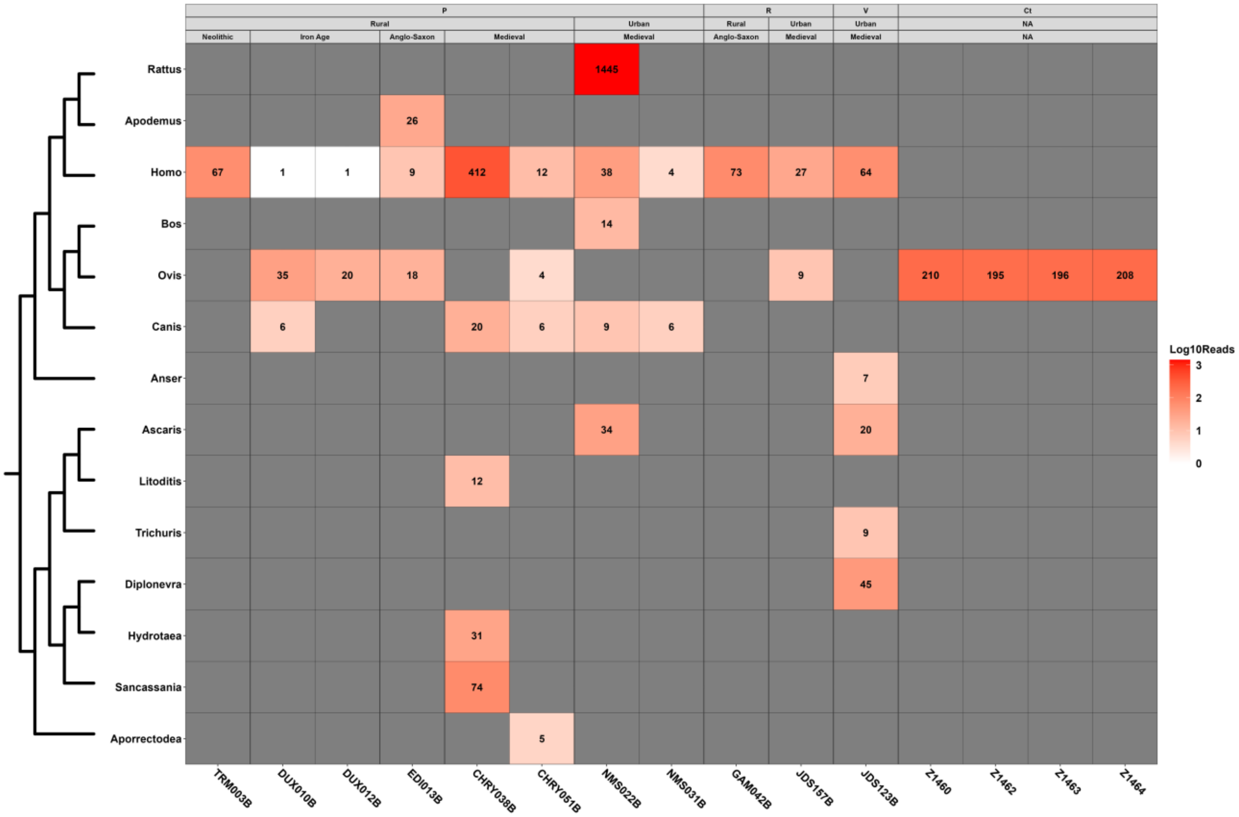

Figure S28. Zoom in of Animal mitochondrial sequences found in the samples (including controls). The number of mapped reads is displayed inside each cell. The vertical axis is ordered by the taxonomic location of detected species. Samples are grouped by their sampling origin (P = Petrous Bone Soil, R = Rib Soil, V = Vertebrae Soil, Ct = Control), context of site (Rural vs Urban), and site datation (Neolithic, Iron Age, Anglo-Saxon and Medieval). Read numbers are in a log10 scale with higher numbers displayed in a darker red. Z1460, Z1462, Z1463, and Z1464 are positive controls.

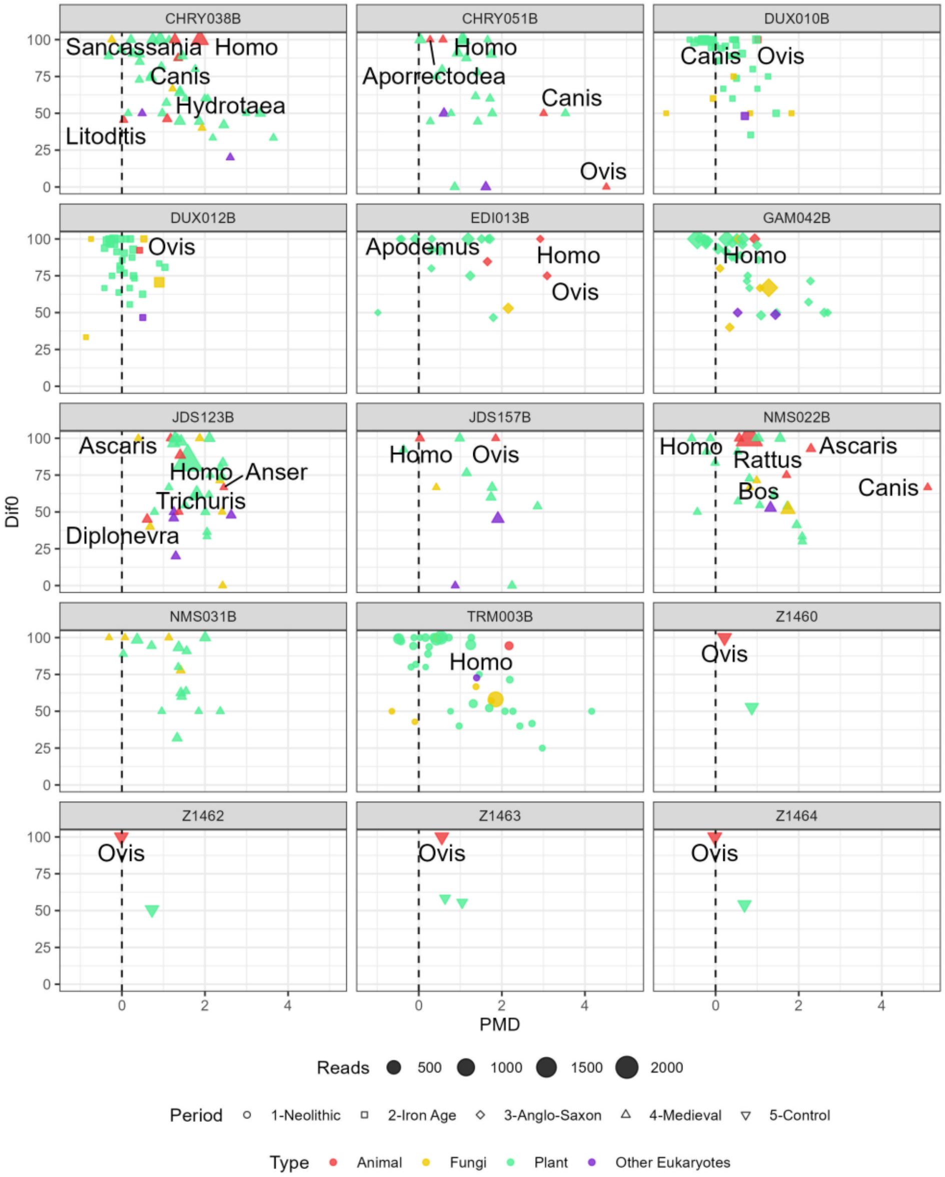

Figure S29. Edit Distance differential against PMD score in retrieved organelle sequences per genus. Size depicts number of sequences and colour taxonomic classification. Animal species are annotated in the plot. Higher PMD value denotes higher damage in the sequences, while higher differential indicates better affinity to the reference genome. Z1460, Z1462, Z1463, and Z1464 are positive controls.

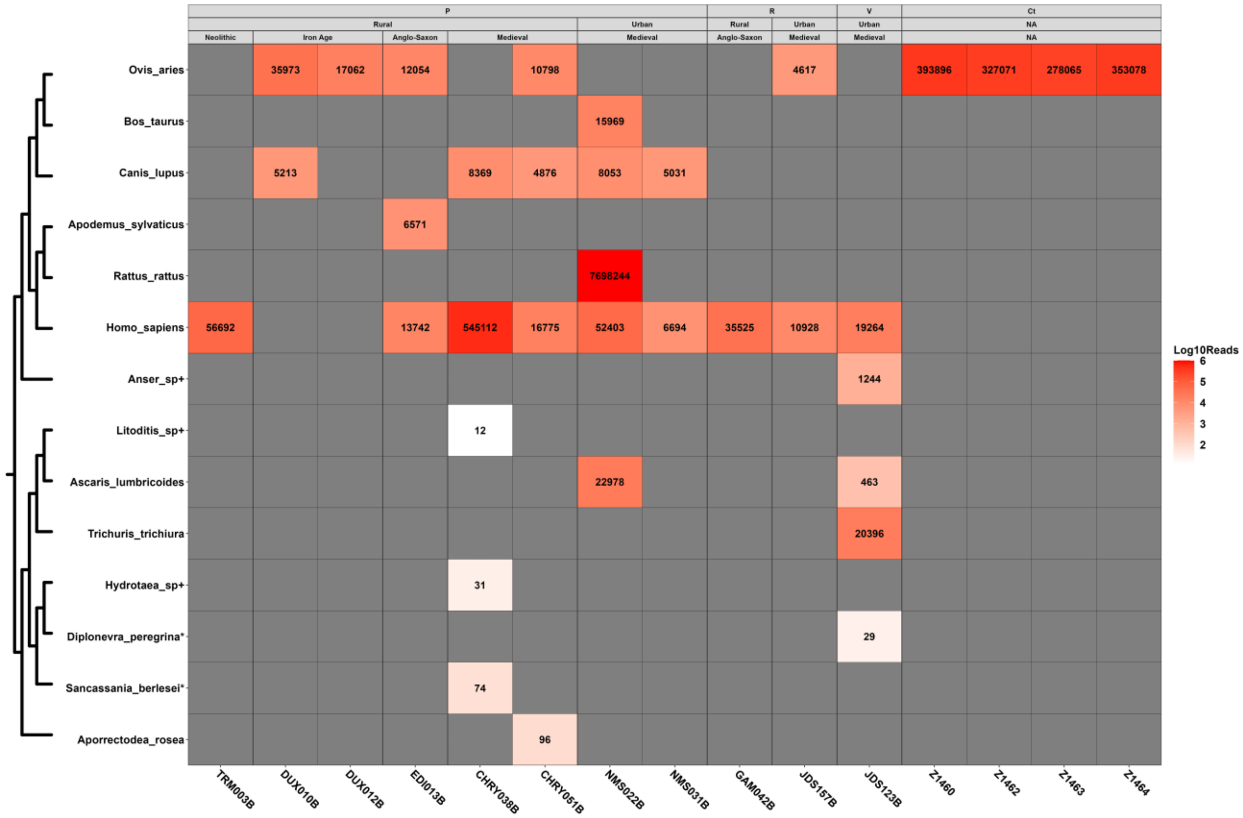

Figure S30. Number of reads from a select panel of animal species present in the samples and controls. The vertical axis is ordered by the taxonomic location of detected species, with some species marked by * (Only mitochondrial reference available) and + (Taxonomic level could not be determined below genus level). Horizontal axis is the sample. Samples are grouped by their sampling origin (P = Petrous Bone Sediment, R = Rib Sediment, V = Vertebrae Sediment, Ct = Control), context of site (Rural vs Urban), and site datation (Neolithic, Iron Age, Anglo-Saxon and Medieval). Read numbers are in a log10 scale with higher numbers displayed in a darker red. Z1460, Z1462, Z1463, and Z1464 are positive controls.

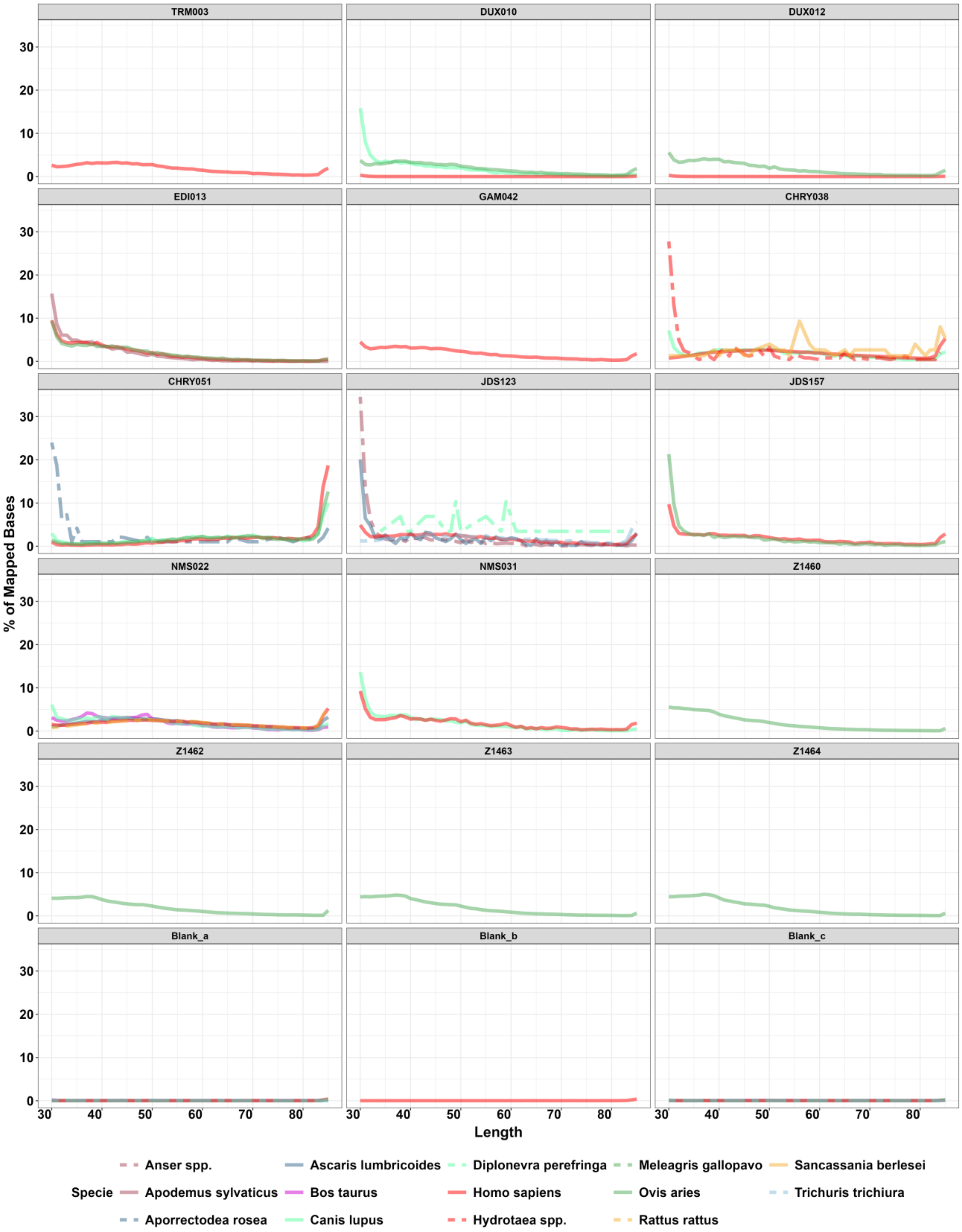

Figure S31. Read length distributions for each species per sample. Z1460, Z1462, Z1463, and Z1464 are positive controls.

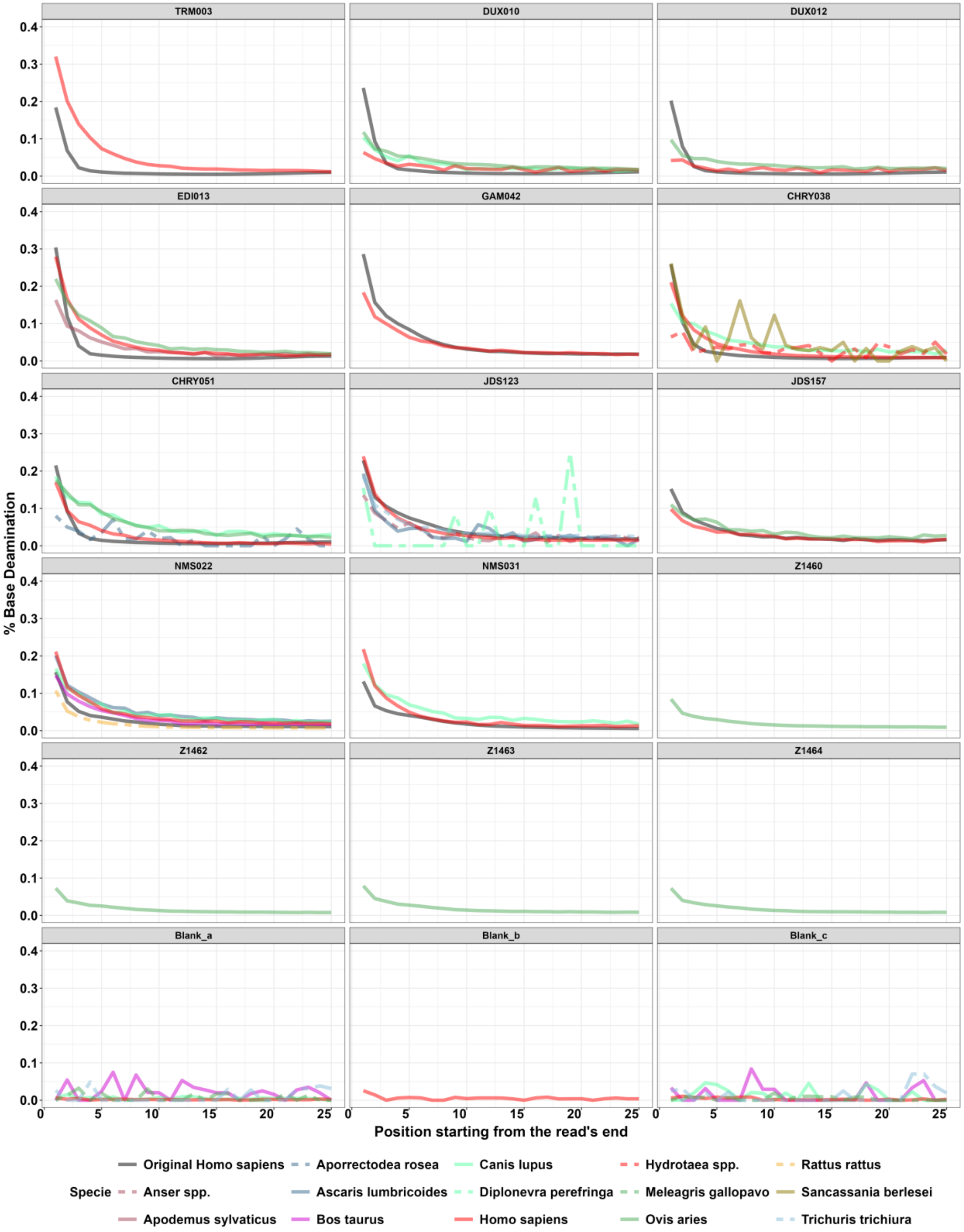

igure S32. Damage patterns from each detected species in each sample. Z1460, Z1462, Z1463, and Z1464 are positive controls.

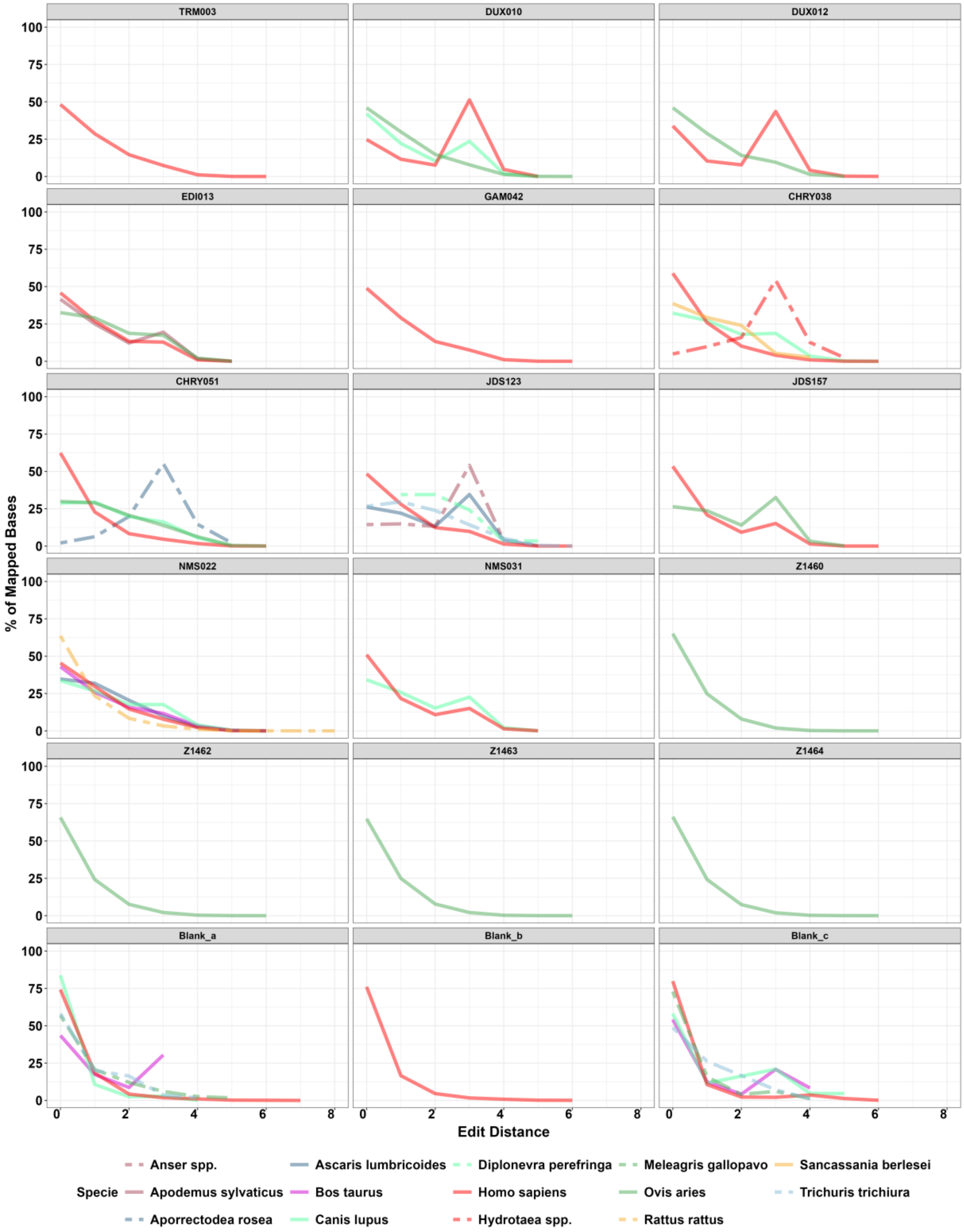

Figure S33. Edit distances from each detected species in each sample. Z1460, Z1462, Z1463, and Z1464 are positive controls.

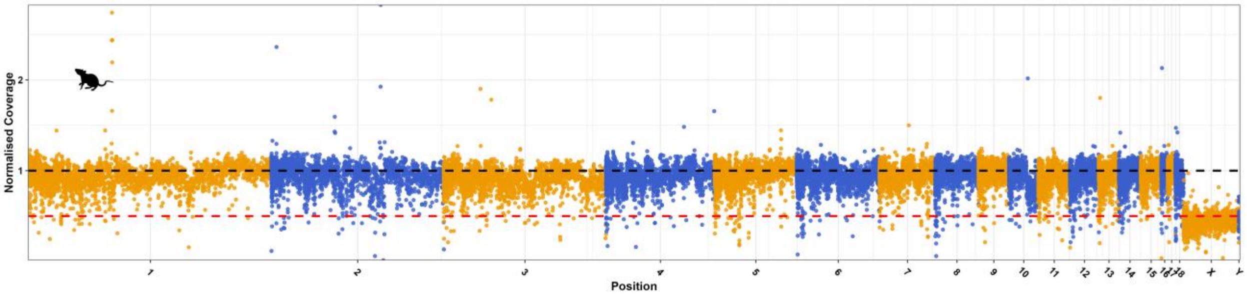

Figure S34. Normalised coverage distribution for *Rattus rattus* genome. Chromosome X and Y present a normalised coverage of approximately 0.5, indicating that the individual is most probably a male.

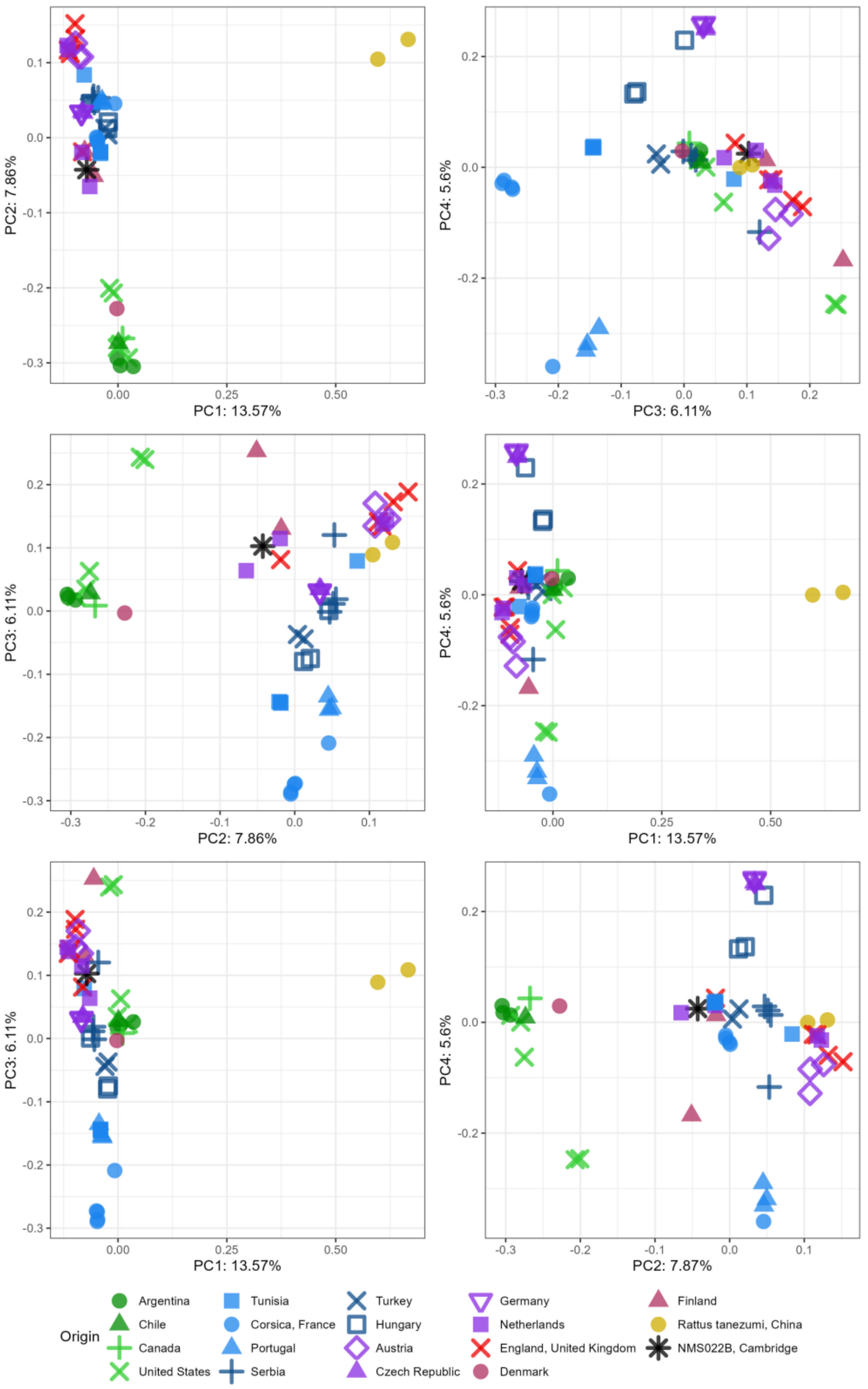

Figure S35. Major components in the *Rattus rattus* PCA, including PC1, PC2, PC3 and PC4.

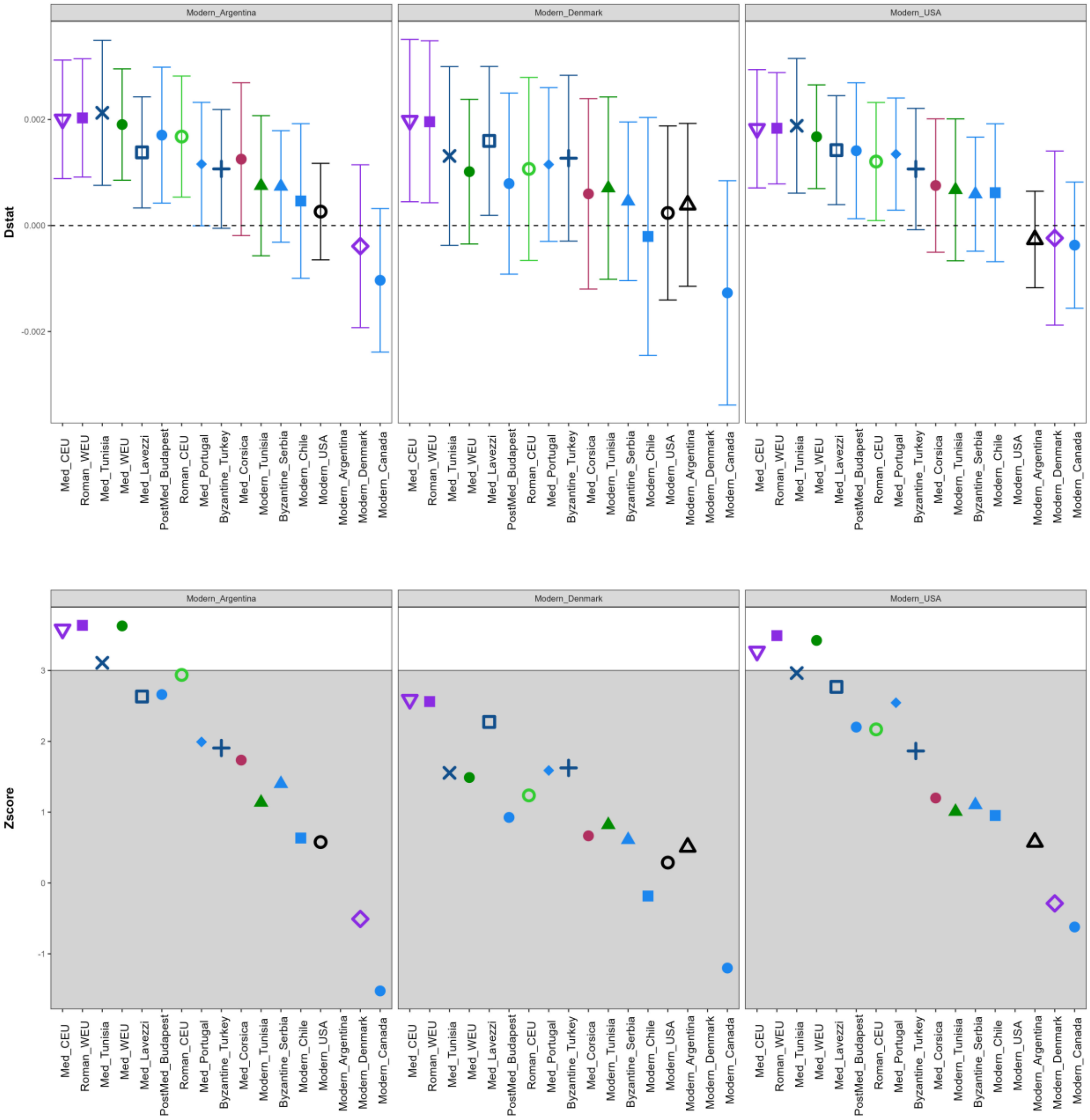

Figure S36. Additional tree structures tested with AdmixTools *Dstat* amd statistical significance assessed using block jack-knife resampling. The tested structures were (Rattus tanemuzi, NMS022B, Modern_Denmark, X), (Rattus tanemuzi, NMS022B, Modern_Argentina, X), and (Rattus tanemuzi, NMS022B, Modern_USA, X).

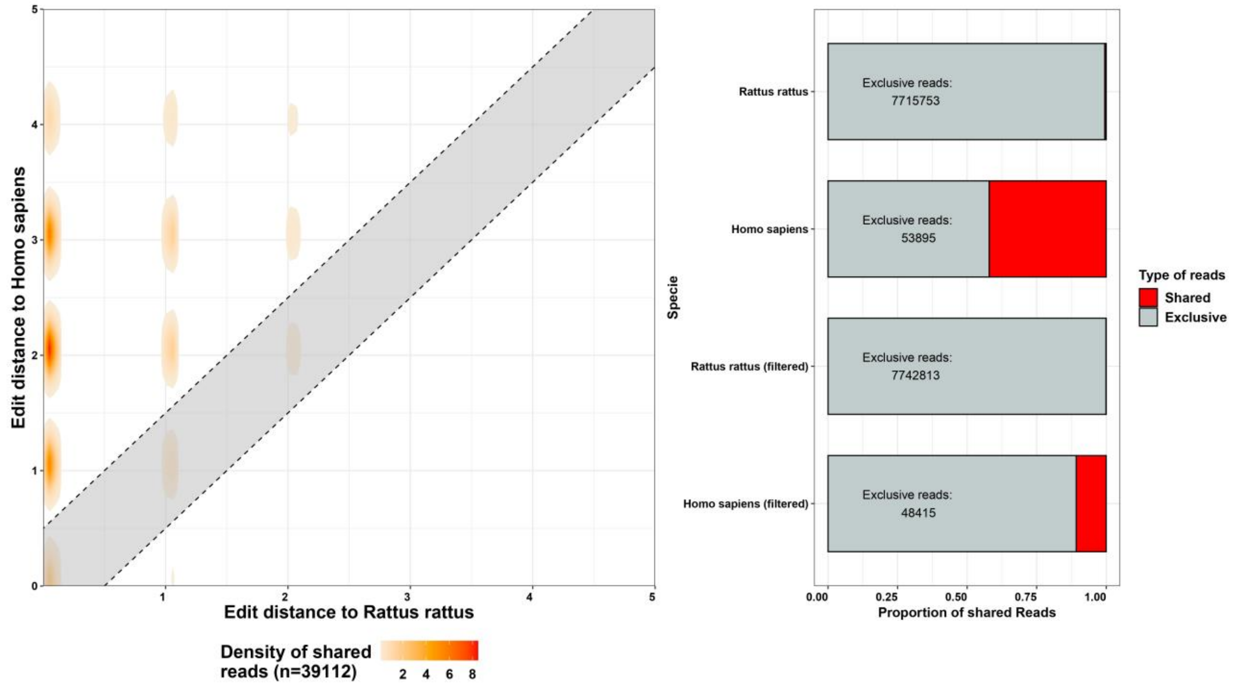

Figure S37. Example of cross mapping between 2 evolutionary close species, *Homo sapiens* and *Rattus rattus.* Approximately 40% of the sequences mapping to *H. sapiens* at NMS022B also map against *R. rattus.* Applying a filter based on edit distance to the reference genome, we can reduce this value to 10%.

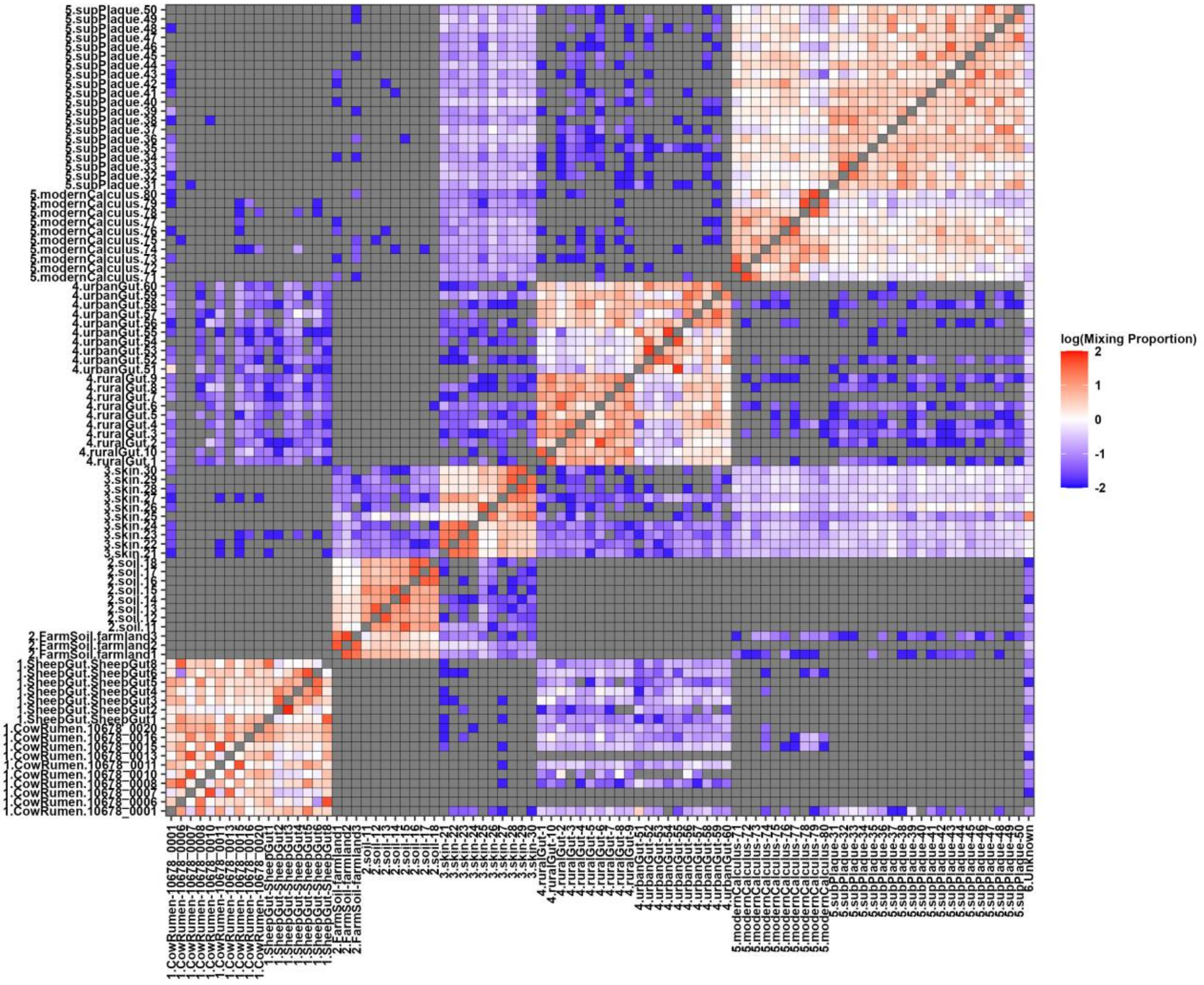

Figure S38. Microbial abundances affinity as estimated by sourcetracker2 "*leave one out*" function in the different reference microbiome sources.

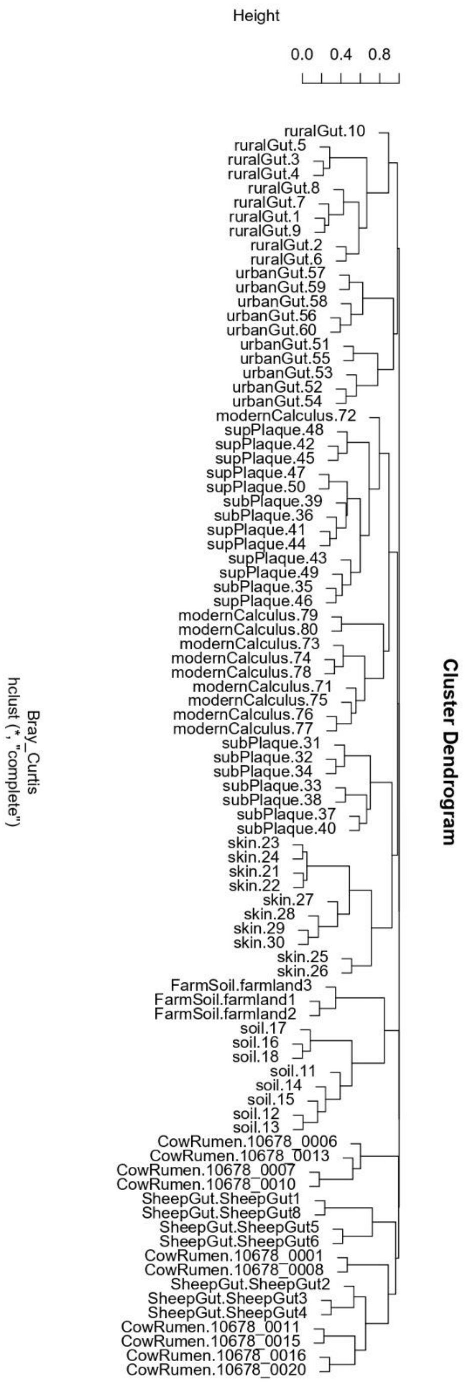

Figure S39. Hierarchical clustering analysis of the Bray-Curtis distances from the different microbial abundances in the Sourcetracker2 reference sources.

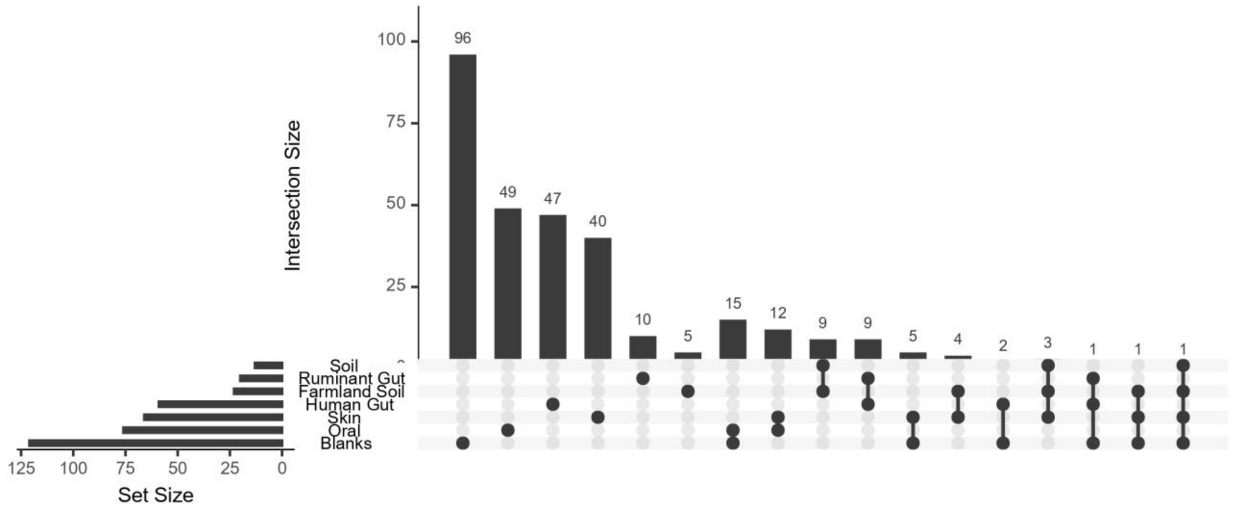

Figure S40. Number of characteristic microbial species determined by sourcetracker2 and overlap with blanks.

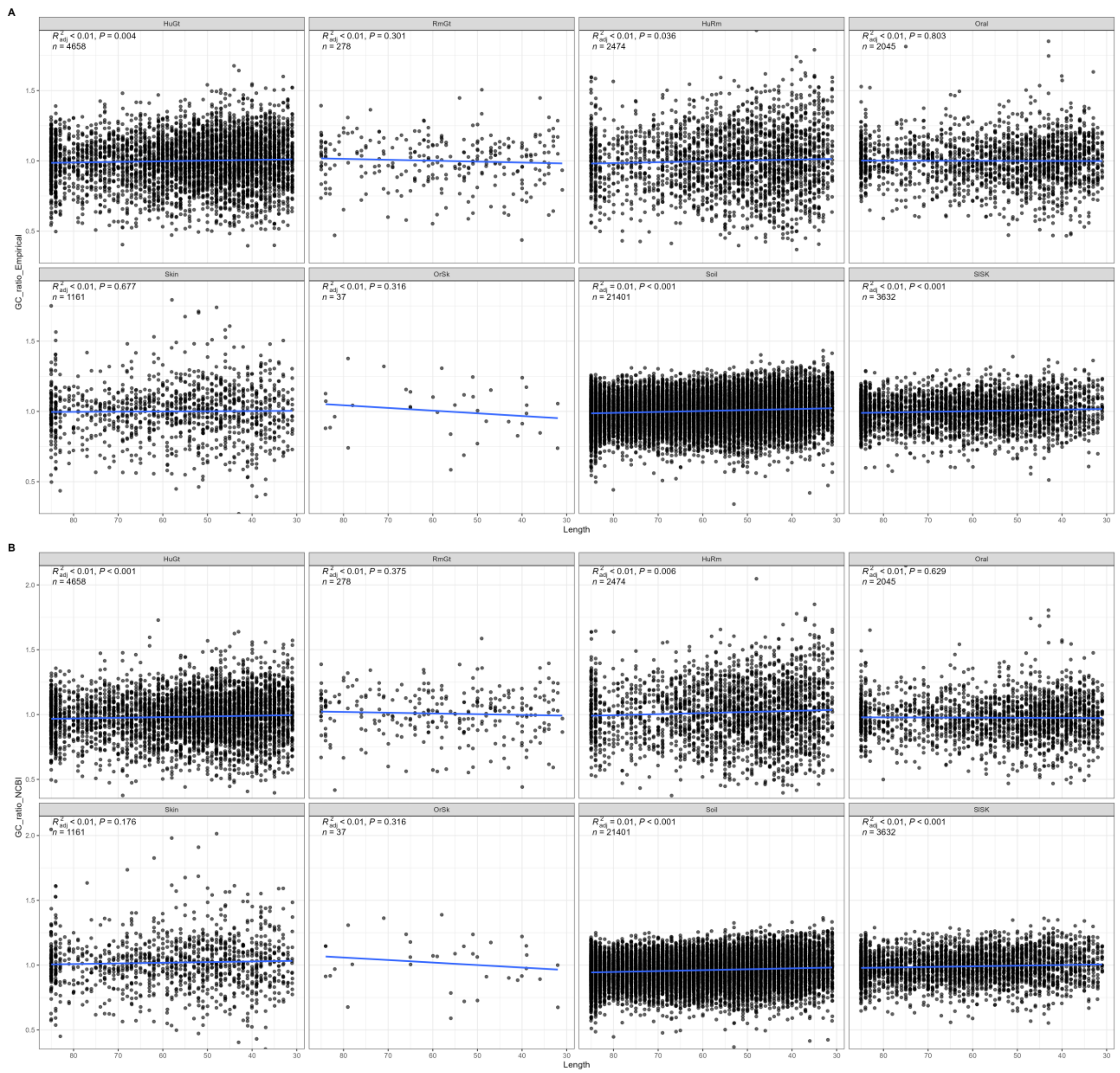

Figure S41. GC content distribution across read length of source-specific microbial species with more than 10 mapped reads after KrakenUniq E-score filtering above 7. GC content bias associated was tested using a linear regression in R. Panel A) shows GC content normalised to its empirical observed value while B) shows GC content normalised to the assembly GC ratio stated in NCBI.

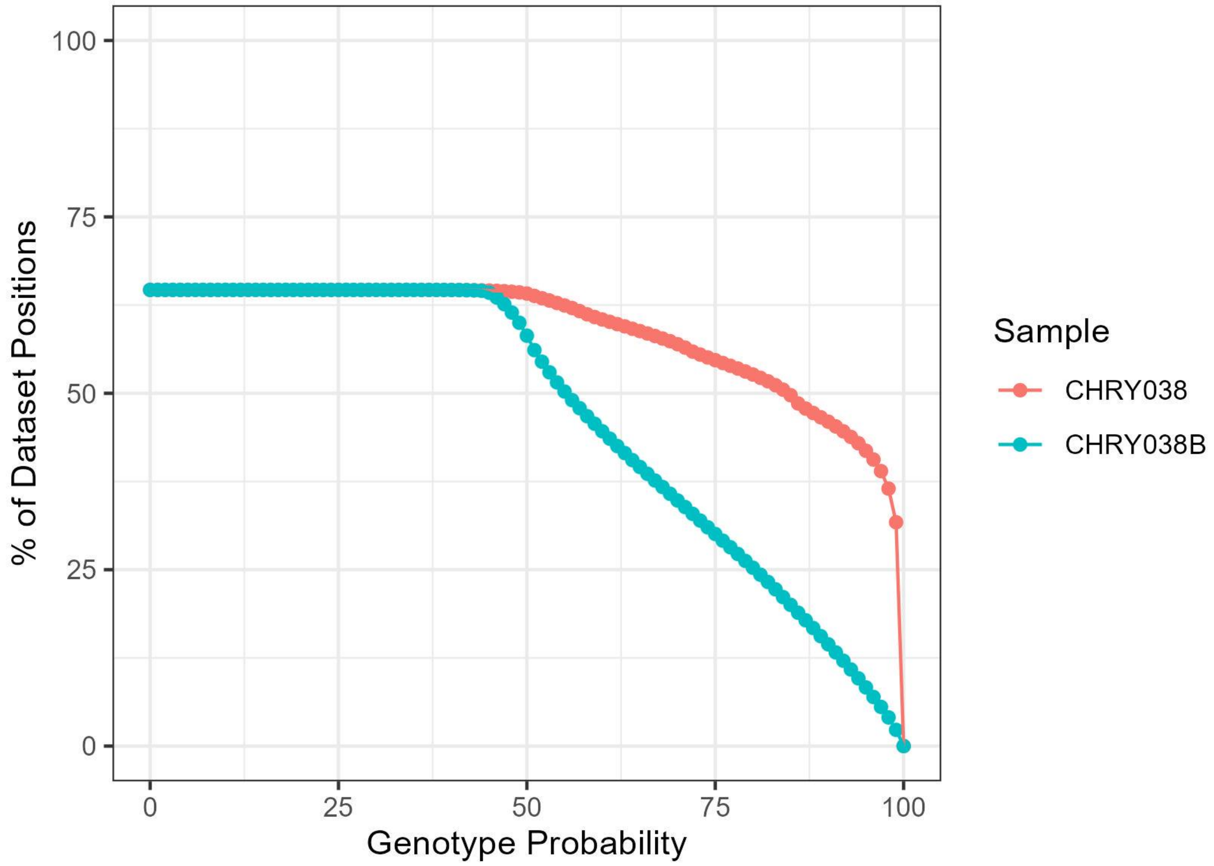

Figure S42. Percentage of the HO dataset SNPs covered after imputation in the original petrous bone sequenced (CHRY038) and in the adhered sediments (CHRY038B) in relation to the genotype probability. The number of covered SNPs in CHRY038B rapidly decay with more stringent filtering values, most probably caused by the low number of initial SNPs (8,580).

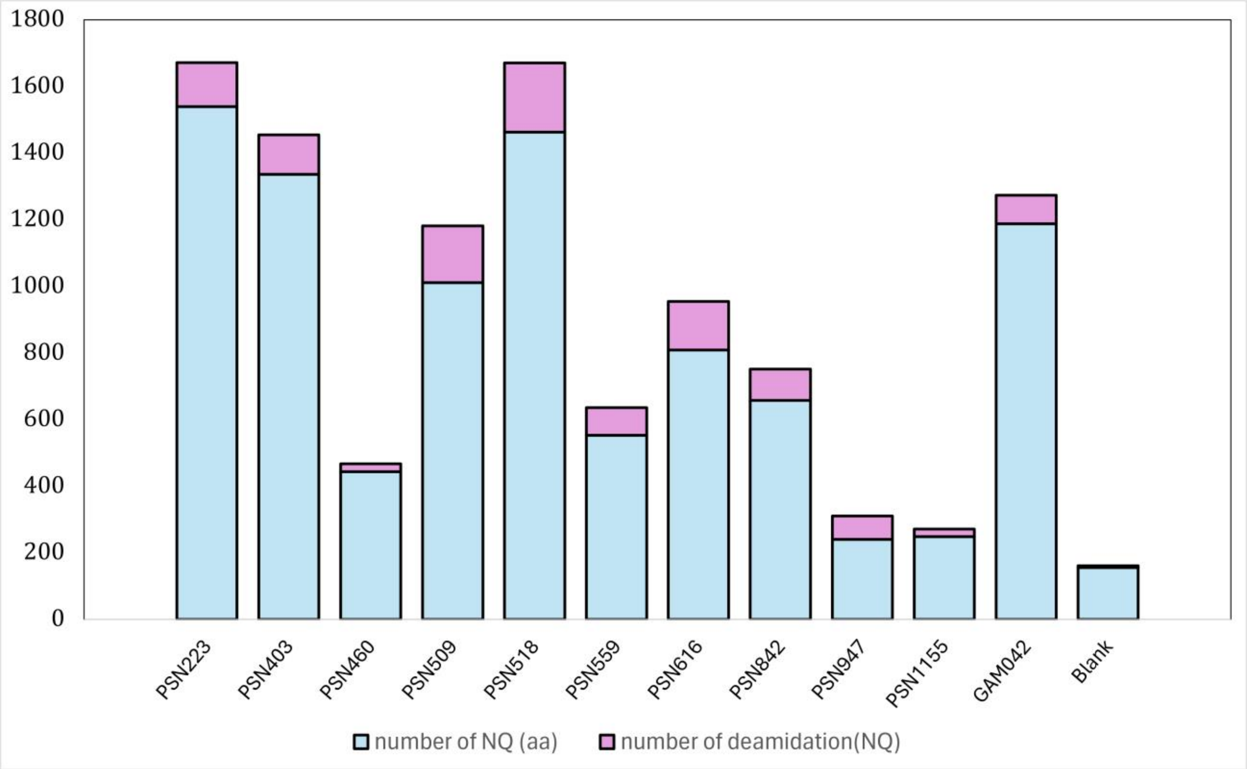

Figure S43. Number of deamidation per Glutamine/Asparagine identified in the ancient sediment samples (PSN; 223 - JDS123B; 403 - JDS157B; 548 – DUX010B; 460 – DUX012B; 509 - NMS031B; 518 – NMS022B; 559 – EDI013B; 616 – TRM003B; 842 – GAM042B; 947 – CHR038B; 1155 – CHRY051B) and skeletal element (GAM042) metaproteomic analysis, only suggestive of postmortem modification of the protein and age since deamidation can happen *in vivo*, and is affected by protein extraction methods or the chemistry of the sediment were they were deposited. Despite this, extraction blank displays minimal NQ deamidation in comparison to our sediment and bone samples.

Supplementary Tables

Table S1. Raw sequences generated for each sample before and after prefiltering. All pre-filtered reads are at least 30bp and quality 30 as stated in the main methods section

Sample	Raw Reads	Unique High Complexity Reads
CHRY038B	19,964,480	17,88,2745
CHRY051B	21,743,979	20,161,737
DUX010B	17,455,2580	14,704,196
DUX012B	15,229,7970	13,046,691
EDI013B	19,470,0280	14,815,381
GAM042B	19,753,5280	15,138,649
JDS123B	18,537,8100	15,643,459
JDS157B	17,856,6780	13,749,513
NMS022B	20,149,396	17,253,437
NMS031B	16,004,4520	10,258,581
TRM003B	17,596,592	14,956,668
GAM042	32,248,147	18,431,240
JDS157A	16,405,420	4,477,441
Z1460 (Control-1)	19,823,619	9,997,561
Z1462 (Control-2)	18,134,387	9,165,025
Z1463 (Control-3)	18,938,113	9,557,912
Z1464 (Control-4)	20,088,743	10,202,713

Table S2. Proteomics screening results and specific protein identification.

Table S3. Human mapping statistics and sex determination.

Table S4. Readv2 results.

Table S5. Human population genetics dataset.

Table S6. CHRY038 Original and Soil phenotypic comparison.

Table S7. Sourcetracker2 results; bacterial species abundances.

Table S8. KrakenUniq classified Genus' sequences of microbial Pathogens. Sequences were extracted and validated by blastn.

Sample	KrUn Reads	Genus	Species	Blast Reads
JDS175B	236	*Yersinia*	*Yersinia enterocolitica*	10
NMS022B	91	*Acanthamoeba*	*Acanthamoeba castellanii*	5
TRMO03B	20	*Entamoeba*	-	0
JDS123B	10	*Giardia*	-	2
CHRY051B	53	*Mycobacterium*	*Mycobacterium leprae*	30

Table S9. Bacterial damages raw data.

Table S10. Eukaryotic screening reference genomes.

Table S11. *Rattus* population genetics dataset.

Table S12. *Rattus rattus* proteins identified from the NMS0022B sample with more than 5% coverage. The identification of tissues in which the proteins are expressed in rats or mice was done using the Uniprot database.

Species	Protein	Coverage with *novor cloud*	coverage with *Pfind*	Expressed in
Rattus rattus	alpha-2-Hs-glycoprotein isoform XI	70.2%	73.6%	Blood
Rattus rattus	collagen alpha-2 (I) chain	74.1%	73.1%	Tissues
Rattus rattus	collagen alpha-1 (I) chain	71.4%	72.5%	Tissues
Rattus rattus	alpha-2-Hs-glycoprotein isoform	NA	72%	Blood
Rattus rattus	collagen alpha-1 (II) chain	67.1%	70.6%	Chondrocytes
Rattus rattus	biglycan	46.1%	50.7%	Tissues
Rattus rattus	chondroadherin	35.9%	33.9%	Cartilage/bone/bone marrow
Rattus rattus	secreted phosphoprotein	28.6%	28.6%	Tissues
Rattus rattus	collagen alpha-3 (VI) chain	24%	27.4%	NA
Rattus rattus	matrix Gla protein	24.3%	27.2%	NA
Rattus rattus	collagen alpha-1 (IX) chain	22.5%	26.3%	NA
Rattus rattus	pigment epithelium-derived factor	16.7%	22.5%	Tissues
Rattus rattus	hyaluroan and proteoglycan link protein 1	21.5%	21.5%	Tissues
Rattus rattus	osteopotin	5.7%	20.8%	NA
Rattus rattus	fibromodulin	20.5%	20.5%	NA
Rattus rattus	SPARC	24.3%	19.9%	Tissues

Rattus rattus	collagen alpha-3 (IX) chain	19%	19%	NA
Rattus rattus	cartilage oliogomeric matrix protein	24.9%	18.5%	NA
Rattus rattus	collagen alpha-2 (XI) chain	12.2%	16.4%	Tissues
Rattus rattus	C-type lectin domain family 3 member A	16.3%	16.3%	NA
Rattus rattus	collagen alpha-2 (IX) chain	10.5%	16%	NA
Rattus rattus	collagen alpha-1 (XII) chain	16.4%	15.1%	NA
Rattus rattus	lactadherin	11%	14.7%	Tissues
Rattus rattus	phosphoethanolamine/phosphocholine phosphatase isoform X2	21%	13.1%	NA
Rattus rattus	cartilage intermediate layer protein 2	8%	13%	Tissues
Rattus rattus	matrilin-3	NA	12.9%	Tissues
Rattus rattus	serum albumin	11.7%	12.8%	Blood
Rattus rattus	osteomodulin	12.5%	12.5%	Tissues
Rattus rattus	phosphoethanolamine/phosphocholine phosphatase isoform X1	NA	12.1%	NA
Rattus rattus	coagulation factor VII	9.4%	11.9%	Blood
Rattus rattus	fibronectin isoform X10	8.5%	11.9%	NA
Rattus rattus	collagen-alpha-1 (III) chain	10.7%	10.9%	Tissues
Rattus rattus	collagen alpha-1 (XI) chain	6.9%	10.6%	Tissues
Rattus rattus	vitronectin	11.3%	9.9%	Tissues

Rattus rattus	collagen alpha-2 (V) chain	4.7%	8.9%	Tissues
Rattus rattus	annexin A1	8.7%	8.7%	Blood
Rattus rattus	prolargin	9.3%	7.2%	Tissues
Rattus rattus	EMILIN-1 isoform X2	2.3%	6.9%	NA
Rattus rattus	collagen alpha-1 (X) chain	10.7%	6.3%	Tissues
Rattus rattus	basement membrane-specific heparan sulfate proteoglycan core protein	5.3%	5.7%	NA
Rattus rattus	aggrecan core protein	5.5%	5.7%	NA
Rattus rattus	periostin isoform X4	2.8%	5.7%	NA

Table S13. Laboratory workflow data.

Table S14. Information of the software used

Software	Version	Reference
AdapterRemoval2	2.3.3	(Schubert, Lindgreen, and Orlando 2016)
prinseq	0.20.04	(Schmieder and Edwards 2011)
bbmap	38.18	(Bushnell 2015)
Kraken2	2.1.2	(Wood, Lu, and Langmead 2019)
BWA	0.7.17-r1188	(Li and Durbin 2009; Li 2013)
KrakenUniq	1.0.4	(Breitwieser, Baker, and Salzberg 2018; Pockrandt, Zimin, and Salzberg 2022)
Sourcetracker2	2.0.1.dev0	(Knights et al. 2011)
vegan	vegan 2.6-4	(Dixon 2003)
krakentools	1.2	(Lu et al. 2022)
MapDamage2	2.2.1	(Jónsson et al. 2013)
Qualimap2	2.2.2-dev	(Okonechnikov, Conesa, and García-Alcalde 2016)
pmdtools	0.60	(Skoglund et al. 2014)
compositions	2.0-8	(van den Boogaart and Tolosana-Delgado 2008)
mixOmics	6.23.4	(Rohart et al. 2017)
BLAST	2.15.0	(Altschul et al. 1990)
MEGAN6	6.25.1	(Beier, Tappu, and Huson 2017)
schmutzi	1.5.7	(Renaud et al. 2015)
GATK	3.7	(McKenna et al. 2010)
RAxML	8.2.12	(Stamatakis 2014)
haplogrep3	3.2.1	(Schönherr et al. 2023)
bcftools	1.8	(Danecek et al. 2021)

Software	Version	Reference
ry_compute	0.4	(Skoglund et al. 2013)
angsd	0.940-dirty	(Korneliussen, Albrechtsen, and Nielsen 2014)
pcangsd	0.940-dirty	(Meisner and Albrechtsen 2018)
AdmixtTools	7.0.2	(Patterson et al. 2012)
sequenceTools	1.5.2	(Schiffels, n.d.)
PLINK	1.90b6.21	(Purcell et al. 2007)
QUILT	1.0.2	(Davies et al. 2021)
Eigensoft (smartpca)	8.0.0	(Price et al. 2006)
READv2	2.01	(Alaçamlı et al. 2024)
pFind	3.2.0	(Wang et al. 2007)
picard	2.20.8	(“Picard,” n.d.)
SAMtools	1.12	(Danecek et al. 2021)
decontam	1.20.0	(Davis et al. 2018)
ggtree	3.8.2	(Yu et al. 2017)
cluster	2.1.6	(Rousseeuw et al. 2023)
IGV (web)	https://igv.org	(Robinson et al. 2011)

Table S14. Original skeletal element samples.

Sample ID	Alternative ID	Skeletal Element	Reference
CHRY038	PSN947	Petrous bone	(Hui et al. 2024)
CHRY051	PSN1155	Petrous bone	(Hui et al. 2024)
DUX010	PSN458	Petrous bone	(Scheib et al. 2023)
DUX012	PSN460	Petrous bone	(Scheib et al. 2023)
EDI013	PSN559	Petrous bone	(Keller et al. 2019)
GAM042	PSN842	Rib	This article
JDS123	J-07 / PSN223	Vertebrae	(Hui et al. 2024)
JDS157A	PSN403	Rib	This article
NMS022	PSN518	Petrous bone	(Hui et al. 2024)
NMS031	PSN509	Petrous bone	(Hui et al. 2024)
TRM003	PSN616	Petrous bone	(Scheib et al. 2023)

Table S16. Metagenomic sources used for microbial source-tracking analysis.

SampleID	Source	Environment	SRA	Publication
modernCalculus-71	Calculus	Oral	ERS3395764	Velsko 2019
modernCalculus-72	Calculus	Oral	ERS3395765	Velsko 2019
modernCalculus-73	Calculus	Oral	ERS3395766	Velsko 2019
modernCalculus-74	Calculus	Oral	ERS3395767	Velsko 2019
modernCalculus-75	Calculus	Oral	ERS3395768	Velsko 2019
modernCalculus-76	Calculus	Oral	ERS3395769	Velsko 2019
modernCalculus-77	Calculus	Oral	ERS3395770	Velsko 2019
modernCalculus-78	Calculus	Oral	ERS3395771	Velsko 2019
modernCalculus-79	Calculus	Oral	ERS3395772	Velsko 2019
modernCalculus-80	Calculus	Oral	ERS3395773	Velsko 2019
ruralGut-10	Human_ruralGut	Human gut	SRR1930145	Rampelli 2015
ruralGut-1	Human_ruralGut	Human gut	SRR1761698	Rampelli 2015
ruralGut-2	Human_ruralGut	Human gut	SRR1761705	Rampelli 2015
ruralGut-3	Human_ruralGut	Human gut	SRR1761710	Rampelli 2015
ruralGut-4	Human_ruralGut	Human gut	SRR1761718	Rampelli 2015
ruralGut-5	Human_ruralGut	Human gut	SRR1761721	Rampelli 2015
ruralGut-6	Human_ruralGut	Human gut	SRR1929408	Rampelli 2015
ruralGut-7	Human_ruralGut	Human gut	SRR1930121	Rampelli 2015
ruralGut-8	Human_ruralGut	Human gut	SRR1930123	Rampelli 2015
ruralGut-9	Human_ruralGut	Human gut	SRR1930141	Rampelli 2015
skin-21	Skin	Skin	SRR1631060	Oh 2016
skin-22	Skin	Skin	SRR1631061	Oh 2016
skin-23	Skin	Skin	SRR1631063	Oh 2016
skin-24	Skin	Skin	SRR1631064	Oh 2016
skin-26	Skin	Skin	SRR3184100	Oh 2016
skin-27	Skin	Skin	SRR3184876	Oh 2016
skin-28	Skin	Skin	SRR3189411	Oh 2016

skin-29	Skin	Skin	SRR3189416	Oh 2016
skin-30	Skin	Skin	SRR3189418	Oh 2016
soil-11	Soil	Soil	ERR671927	Bissett 2016
soil-12	Soil	Soil	ERR671931	Bissett 2016
soil-13	Soil	Soil	ERR671933	Bissett 2016
soil-14	Soil	Soil	ERR671934	Bissett 2016
soil-15	Soil	Soil	ERR671935	Bissett 2016
soil-16	Soil	Soil	ERR671936	Bissett 2016
soil-17	Soil	Soil	ERR671938	Bissett 2016
soil-18	Soil	Soil	ERR687883	Bissett 2016
subPlaque-31	SubPlaque	Oral	SRR061294	HMP 2012
subPlaque-32	SubPlaque	Oral	SRR062298	HMP 2012
subPlaque-33	SubPlaque	Oral	SRR062299	HMP 2012
subPlaque-34	SubPlaque	Oral	SRR513165	HMP 2012
subPlaque-35	SubPlaque	Oral	SRR513768	HMP 2012
subPlaque-36	SubPlaque	Oral	SRR513775	HMP 2012
subPlaque-37	SubPlaque	Oral	SRR514202	HMP 2012
subPlaque-38	SubPlaque	Oral	SRR514239	HMP 2012
subPlaque-39	SubPlaque	Oral	SRR514306	HMP 2012
subPlaque-40	SubPlaque	Oral	SRR514329	HMP 2012
supPlaque-41	SupPlaque	Oral	SRR061192	HMP 2012
supPlaque-42	SupPlaque	Oral	SRR061320	HMP 2012
supPlaque-43	SupPlaque	Oral	SRR061365	HMP 2012
supPlaque-44	SupPlaque	Oral	SRR061562	HMP 2012
supPlaque-45	SupPlaque	Oral	SRR062083	HMP 2012
supPlaque-46	SupPlaque	Oral	SRR063517	HMP 2012
supPlaque-47	SupPlaque	Oral	SRR1804664	HMP 2012
supPlaque-48	SupPlaque	Oral	SRR1804823	HMP 2012
supPlaque-49	SupPlaque	Oral	SRR512767	HMP 2012

supPlaque-50	SupPlaque	Oral	SRR513828	HMP 2012
urbanGut-51	Human_urbanGut	Oral	SRR059389	HMP 2012
urbanGut-52	Human_urbanGut	Oral	SRR059425	HMP 2012
urbanGut-53	Human_urbanGut	Oral	SRR059455	HMP 2012
urbanGut-54	Human_urbanGut	Oral	SRR059917	HMP 2012
urbanGut-55	Human_urbanGut	Oral	SRR060358	HMP 2012
urbanGut-56	Human_urbanGut	Oral	SRR1761677	HMP 2012
urbanGut-57	Human_urbanGut	Oral	SRR1761682	HMP 2012
urbanGut-58	Human_urbanGut	Oral	SRR1761688	HMP 2012
urbanGut-59	Human_urbanGut	Oral	SRR1761692	HMP 2012
urbanGut-60	Human_urbanGut	Oral	SRR1761697	HMP 2012
CowRumen-10678_0008	Ruminant_Gut	Ruminant Gut	ERR3201382	Stewart 2019
CowRumen-10678_0010	Ruminant_Gut	Ruminant Gut	ERR3201384	Stewart 2019
CowRumen-10678_0011	Ruminant_Gut	Ruminant Gut	ERR3201385	Stewart 2019
CowRumen-10678_0015	Ruminant_Gut	Ruminant Gut	ERR3201388	Stewart 2019
CowRumen-10678_0016	Ruminant_Gut	Ruminant Gut	ERR3201389	Stewart 2019
CowRumen-10678_0020	Ruminant_Gut	Ruminant Gut	ERR3201390	Stewart 2019
FarmSoil-farmland1	Farm_Soil	Soil	SRR22318749	China Agricultural University
FarmSoil-farmland2	Farm_Soil	Soil	SRR22318750	China Agricultural University
FarmSoil-farmland3	Farm_Soil	Soil	SRR22318751	China Agricultural University
SheepGut-SheepGut1	Ruminant_Gut	Ruminant Gut	SRR17509605	Su 2022
SheepGut-SheepGut2	Ruminant_Gut	Ruminant Gut	SRR17509606	Su 2022
SheepGut-SheepGut3	Ruminant_Gut	Ruminant Gut	SRR17509607	Su 2022
SheepGut-SheepGut4	Ruminant_Gut	Ruminant Gut	SRR17509608	Su 2022
SheepGut-SheepGut5	Ruminant_Gut	Ruminant Gut	SRR17509609	Su 2022
SheepGut-SheepGut6	Ruminant_Gut	Ruminant Gut	SRR17509611	Su 2022
SheepGut-SheepGut8	Ruminant_Gut	Ruminant Gut	SRR17509620	Su 2022

Table S17. Cherry Hinton samples used for kinship analysis. It includes dataset coverage, Number of 1240K SNPs covered per sample, range of overlap SNPs used in READv2 and overlap in READv2 for different iterations of sample CHRY038 (Original Petrous bone - CHRY038, Genotype probability of 98% Imputed Petrous bone - CHRY038-P98, Sediment from Petrous bone - CHRY038B, and Genotype probability of 98% Imputed Sediment from Petrous Bone - CHRY038B-P98).

Individual	Dataset Coverage	Number SNPs	Range Overlap Readv2	Overlap CHRY038B-P98	Overlap CHRY038-P98	Overlap CHRY038B	Overlap CHRY038
CHRY001	25.9%	311,329	4,184-99,368	14,145	73,018	4,184	23,515
CHRY003	8.94%	107,452	1,502-34,123	4,972	25,242	1,502	8,199
CHRY005	8.7%	104,646	1,415-34,143	4,84	24,687	1,415	8,013
CHRY009	24.15%	290,326	3,876-92,313	13,237	67,677	3,876	22,145
CHRY011	9.82%	118,009	1,715-40,202	5,325	27,869	1,715	9,391
CHRY012	9.32%	112,02	1,503-37,449	5,062	26,474	1,503	8,915
CHRY015	6.31%	75,795	1,010-24,287	3,42	17,77	1,01	5,809
CHRY017	9.25%	111,18	1,453-35,526	5,061	25,789	1,453	8,518
CHRY020	20.29%	243,886	3,304-78,424	11,147	57,082	3,304	18,688
CHRY022	14.4%	173,091	2,385-55,872	7,888	40,427	2,385	13,333
CHRY027	9.47%	113,848	1,528-35,942	5,151	26,298	1,528	8,619
CHRY028	12.61%	151,618	2,034-48,200	6,938	35,531	2,034	11,497
CHRY029	8.79%	105,706	1,423-34,225	4,865	24,677	1,423	8,106
CHRY034	23.03%	276,906	3,751-90,905	12,466	65,018	3,751	21,407
CHRY036	11.19%	134,554	1,858-43,227	6,163	31,518	1,858	10,258
CHRY037	29.09%	349,656	4,621-10,4290	15,824	81,79	4,621	26,848
CHRY041	18.19%	218,727	2,836-69,432	10,024	50,837	2,836	16,467
CHRY046	25.41%	305,491	4,266-104,290	13,866	72,591	4,266	24,385
CHRY050	15.56%	187,04	2,518-62,390	8,446	44,17	2,518	14,823
CHRY058	26.57%	319,457	4,267-101,689	14,683	74,776	4,267	24,251
CHRY038	6.92%	83,187	1,081-26,848	3,809	22,605	1,081	
CHRY038-P98	38.87%	466,63	3,469-81,790	45,08		3,469	22,605
CHRY038B	1.2%	14,466	1,010-4,621	1,42	3,469		1,081
CHRY038B-P98	4.18%	50,248	1,420-45,080		45,08	3,809	1,42

Supplementary Files

File S1. Proteins used to build the *Rattus rattus* and *Rattus norvegicus* comparison database. This database contain protein sequences obtained from the reference genome of Rattus rattus_CSIRO and Rattus norvegicus_GRCr8.

Supplementary Bibliography

Alaçamlı, Erkin, Thijessen Naidoo, Şevval Aktürk, Merve N. Güler, Igor Mapelli, Kıvılcım Başak Vural, Mehmet Somel, Helena Malmström, and Torsten Günther. 2024. "READv2: Advanced and User-Friendly Detection of Biological Relatedness in Archaeogenomics." *bioRxiv*. https://doi.org/10.1101/2024.01.23.576660.

Altschul, S. F., W. Gish, W. Miller, E. W. Myers, and D. J. Lipman. 1990. "Basic Local Alignment Search Tool." *Journal of Molecular Biology* 215 (3): 403–10.

"A New E-Score for KrakenUniq." 2022. *Maxime Borry* (blog). May 10, 2022. https://maximeborry.com/post/kraken-uniq/.

Beier, Sina, Rewati Tappu, and Daniel H. Huson. 2017. "Functional Analysis in Metagenomics Using MEGAN 6." In *Functional Metagenomics: Tools and Applications*, edited by Trevor C. Charles, Mark R. Liles, and Angela Sessitsch, 65–74. Cham: Springer International Publishing.

Bissett, Andrew, Anna Fitzgerald, Thys Meintjes, Pauline M. Mele, Frank Reith, Paul G. Dennis, Martin F. Breed, et al. 2016. "Introducing BASE: The Biomes of Australian Soil Environments Soil Microbial Diversity Database." *GigaScience* 5 (1). https://doi.org/10.1186/s13742-016-0126-5.

Boogaart, K. Gerald van den, and R. Tolosana-Delgado. 2008. "'compositions': A Unified R Package to Analyze Compositional Data." *Computers & Geosciences* 34 (4): 320–38.

Breitwieser, F. P., D. N. Baker, and S. L. Salzberg. 2018. "KrakenUniq: Confident and Fast Metagenomics Classification Using Unique K -Mer Counts." *Genome Biology* 19 (1): 1–10.

Bushnell, Brian. 2015. "BBMap."

Cessford, Craig. 2015. "The St. John's Hospital Cemetery and Environs, Cambridge: Contextualizing the Medieval Urban Dead." *Archaeological Journal* 172 (1): 52–120.

Cessford 2017. "Former Old Examination Hall, North Range Buildings, New Museums Site, Cambridge. An Archaeological Excavation." Cambridge Archaeological Unit, Department of Archaeology, University of Cambridge. https://doi.org/10.17863/CAM.101304.

Cessford, Craig, and Alison Dickens. 2006. "The Manor of Hintona: The Origins and Development of Church End, Cherry Hinton." https://doi.org/10.5284/1073333.

Cessford, Craig, Andrew Hall, Bram Mulder, Benjamin Neil, Ian Riddler, Justin Wiles, with

CONTRIBUTIONS BY, Esther Cameron, and Quita Mould. 2022. "Buried with Their Buckles On: Clothed Burial at the Augustinian Friary, Cambridge." *Medieval Archaeology* 66 (1): 151–87.

Cessford, Craig, and Benjamin Neil. 2022. "The People of the Cambridge Austin Friars." *Archaeological Journal* 179 (2): 385–446.

Cessford, Craig, Mark Samuel, Vicki Herring, Nick Holder, and Philip Mills. 2023. "THE ARCHITECTURE OF THE AUGUSTINIAN FRIARY, CAMBRIDGE." *The Antiquaries Journal*, August, 1–33.

Cessford, Craig, Christiana L. Scheib, Meriam Guellil, Marcel Keller, Craig Alexander, Sarah A. Inskip, and John E. Robb. 2021. "Beyond Plague Pits: Using Genetics to Identify Responses to Plague in Medieval Cambridgeshire." *European Journal of Archaeology*, 1–23.

Cessford, Craig, and Adam Slater. 2015. "Beyond the Manor of Hintona Further Thoughts on the Development of Church End, Cherry Hinton: The Neath Farm Site." https://doi.org/10.5284/1073473.

Cherryson, Annia, and Jo Buckberry. 2010. *Burial in Later Anglo-Saxon England, c.650-1100 AD*. Studies in Funerary Archaeology 4. Oxford, England: Oxbow Books.

Danecek, Petr, James K. Bonfield, Jennifer Liddle, John Marshall, Valeriu Ohan, Martin O. Pollard, Andrew Whitwham, et al. 2021. "Twelve Years of SAMtools and BCFtools." *GigaScience* 10 (2). https://doi.org/10.1093/gigascience/giab008.

Davies, Robert W., Marek Kucka, Dingwen Su, Sinan Shi, Maeve Flanagan, Christopher M. Cunniff, Yingguang Frank Chan, and Simon Myers. 2021. "Rapid Genotype Imputation from Sequence with Reference Panels." *Nature Genetics* 53 (7): 1104–11.

Davis NM, Proctor DM, Holmes SP, Relman DA, Callahan BJ. 2018. Simple statistical identification and removal of contaminant sequences in marker-gene and metagenomics data. *Microbiome* 6:226.

Dixon, Philip. 2003. "VEGAN, a Package of R Functions for Community Ecology." *Journal of Vegetation Science: Official Organ of the International Association for Vegetation Science* 14 (6): 927–30.

Faber, T. E., L. Napran, and T. E. Faber Estate. 2006. *An Intimate History of the Parish of St Clement in Cambridge, 1250-1950*. Privately Published.

Ferrante di Ruffano, L., and T. Waldron. 2006. "The Skeletal Analysis of an Anglo-Saxon Population from Cherry Hinton, Cambridgeshire." *Unpublished Report HAT358/1).*

Archaeological Solutions Ltd. Bury St. Edmunds, England.

Guellil, Meriam, Lucy van Dorp, Sarah A. Inskip, Jenna M. Dittmar, Lehti Saag, Kristiina Tambets, Ruoyun Hui, et al. 2022. “Ancient Herpes Simplex 1 Genomes Reveal Recent Viral Structure in Eurasia.” *Science Advances* 8 (30): eabo4435.

Guellil, Meriam, Marcel Keller, Jenna M. Dittmar, Sarah A. Inskip, Craig Cessford, Anu Solnik, Toomas Kivisild, Mait Metspalu, John E. Robb, and Christiana L. Scheib. 2022. “An Invasive Haemophilus Influenzae Serotype B Infection in an Anglo-Saxon Plague Victim.” *Genome Biology* 23 (1): 1–27.

Guellil, Meriam, Oliver Kersten, Amine Namouchi, Egil L. Bauer, Michael Derrick, Anne Ø. Jensen, Nils C. Stenseth, and Barbara Bramanti. 2018. “Genomic Blueprint of a Relapsing Fever Pathogen in 15th Century Scandinavia.” *Proceedings of the National Academy of Sciences of the United States of America* 115 (41): 10422–27.

Hamerow, Helena. 2012. “Settlement Forms and Community Structures.” In *Rural Settlements and Society in Anglo-Saxon England*, 67–119. Oxford University Press.

Herbig, Alexander, Frank Maixner, Kirsten I. Bos, Albert Zink, Johannes Krause, and Daniel H. Huson. 2016. “MALT: Fast Alignment and Analysis of Metagenomic DNA Sequence Data Applied to the Tyrolean Iceman.” *bioRxiv*. https://doi.org/10.1101/050559.

Hertfordshire Archaeological Trust. 1999. “Land at 69-115 Church End, Cherry Hinton, Cambridge. An Archaeological Evaluation.” Archaeology Data Service. https://doi.org/10.5284/1021789.

Hui, Ruoyun, Christiana L. Scheib, Eugenia D’Atanasio, Sarah A. Inskip, Craig Cessford, Simone A. Biagini, Anthony W. Wohns, et al. 2024. “Genetic History of Cambridgeshire before and after the Black Death.” *Science Advances* 10 (3): eadi5903.

Human Microbiome Project Consortium. 2012. “Structure, Function and Diversity of the Healthy Human Microbiome.” *Nature* 486 (7402): 207–14.

Inskip, Sarah, Craig Cessford, Jenna Dittmar, Alice Rose, Bram Mulder, Piers D. Mitchell, Christiana Scheib, et al. 2023. “Pathways to the Medieval Hospital: Collective Osteobiographies of Poverty and Charity.” *Antiquity* 97 (396): 1581–97.

Jónsson, Hákon, Aurélien Ginolhac, Mikkel Schubert, Philip L. F. Johnson, and Ludovic Orlando. 2013. “mapDamage2.0: Fast Approximate Bayesian Estimates of Ancient DNA Damage Parameters.” *Bioinformatics* 29 (13): 1682–84.

Keller, Marcel, Maria A. Spyrou, Christiana L. Scheib, Gunnar U. Neumann, Andreas Kröpelin,

Brigitte Haas-Gebhard, Bernd Päffgen, et al. 2019. “Ancient Yersinia Pestis Genomes from across Western Europe Reveal Early Diversification during the First Pandemic (541-750).” *Proceedings of the National Academy of Sciences of the United States of America* 116 (25): 12363–72.

Kim, Daehwan, Li Song, Florian P. Breitwieser, and Steven L. Salzberg. 2016. “Centrifuge: Rapid and Sensitive Classification of Metagenomic Sequences.” *Genome Research* 26 (12): 1721–29.

Kirby, T., and S. Oosthuizen, eds. n.d. “An Atlas of Cambridgeshire and Huntingdonshire History (Cambridge, Centre for Regional Studies).” In *Anglia Polytechnic University)*.

Knights, Dan, Justin Kuczynski, Emily S. Charlson, Jesse Zaneveld, Michael C. Mozer, Ronald G. Collman, Frederic D. Bushman, Rob Knight, and Scott T. Kelley. 2011. “Bayesian Community-Wide Culture-Independent Microbial Source Tracking.” *Nature Methods* 8 (9): 761–63.

Korneliussen, Thorfinn Sand, Anders Albrechtsen, and Rasmus Nielsen. 2014. “ANGSD: Analysis of Next Generation Sequencing Data.” *BMC Bioinformatics* 15 (1): 356.

Li, Heng. 2013. “Aligning Sequence Reads, Clone Sequences and Assembly Contigs with BWA-MEM.” *arXiv [q-bio.GN]*. arXiv. http://arxiv.org/abs/1303.3997.

Li, Heng, and Richard Durbin. 2009. “Fast and Accurate Short Read Alignment with Burrows-Wheeler Transform.” *Bioinformatics* 25 (14): 1754–60.

Lloyd-Price, Jason, Anup Mahurkar, Gholamali Rahnavard, Jonathan Crabtree, Joshua Orvis, A. Brantley Hall, Arthur Brady, et al. 2017. “Strains, Functions and Dynamics in the Expanded Human Microbiome Project.” *Nature* 550 (7674): 61–66.

Lu, Jennifer, Natalia Rincon, Derrick E. Wood, Florian P. Breitwieser, Christopher Pockrandt, Ben Langmead, Steven L. Salzberg, and Martin Steinegger. 2022. “Metagenome Analysis Using the Kraken Software Suite.” *Nature Protocols* 17 (12): 2815–39.

Lyons, Alice. 2011. “Life and Afterlife at Duxford, Cambridgeshire: Archaeology and History in a Chalkland Community.” *East Anglian Archaeology*.

Ma, Bin. 2015. “Novor: Real-Time Peptide de Novo Sequencing Software.” *Journal of the American Society for Mass Spectrometry* 26 (11): 1885–94.

Malim, Tim. 1998. *The Anglo-Saxon Cemetery at Edix Hill (Barrington A), Cambridgeshire*. CBA Research Reports, No. 112. York, England: Council for British Archaeology.

McDonald, T., and M. Trevarthen. 1998. “Excavations at Station Road, Gamlingay, Cambridgeshire. Interim Site Narrative (HAT Report No. 317).” 317. Hertfordshire Archaeological Trust.

McKenna, Aaron, Matthew Hanna, Eric Banks, Andrey Sivachenko, Kristian Cibulskis, Andrew Kernytsky, Kiran Garimella, et al. 2010. “The Genome Analysis Toolkit: A MapReduce Framework for Analyzing next-Generation DNA Sequencing Data.” *Genome Research* 20 (9): 1297–1303.

Meisner, Jonas, and Anders Albrechtsen. 2018. “Inferring Population Structure and Admixture Proportions in Low-Depth NGS Data.” *Genetics* 210 (2): 719–31.

Meng, Guanliang, Yiyuan Li, Chentao Yang, and Shanlin Liu. 2019. “MitoZ: A Toolkit for Animal Mitochondrial Genome Assembly, Annotation and Visualization.” *Nucleic Acids Research* 47 (11): e63.

Mukherjee, Supratim, Dimitri Stamatis, Cindy Tianqing Li, Galina Ovchinnikova, Jon Bertsch, Jagadish Chandrabose Sundaramurthi, Mahathi Kandimalla, et al. 2023. “Twenty-Five Years of Genomes OnLine Database (GOLD): Data Updates and New Features in v.9.” *Nucleic Acids Research* 51 (D1): D957–63.

Murray, Jon, and Tom McDonald. 2006. “Excavations at Station Road, Gamlingay, Cambridgeshire.” *Anglo-Saxon Studies in Archaeology and History* 13 (Anglo-Saxon Stud Archaeol Hist 13): 173–330.

Murray and McDonald 2017. “Excavations at Station Road, Gamlingay, Cambridgeshire.” In *Anglo-Saxon Studies in Archaeology and History 13*, 173–330. Oxford University School of Archaeology.

Oh, Julia, Allyson L. Byrd, Morgan Park, NISC Comparative Sequencing Program, Heidi H. Kong, and Julia A. Segre. 2016. “Temporal Stability of the Human Skin Microbiome.” *Cell* 165 (4): 854–66.

Okonechnikov, Konstantin, Ana Conesa, and Fernando García-Alcalde. 2016. “Qualimap 2: Advanced Multi-Sample Quality Control for High-Throughput Sequencing Data.” *Bioinformatics* 32 (2): 292–94.

Patten, R. 2012. “Trumpington Meadows, Cambridge An Archaeological Excavation.” Archaeology Data Service. https://doi.org/10.5284/1092743.

Patterson, Nick, Priya Moorjani, Yontao Luo, Swapan Mallick, Nadin Rohland, Yiping Zhan, Teri Genschoreck, Teresa Webster, and David Reich. 2012. “Ancient Admixture in Human History.” *Genetics* 192 (3): 1065–93.

Pfrengle, Saskia, Judith Neukamm, Meriam Guellil, Marcel Keller, Martyna Molak, Charlotte Avanzi, Alena Kushniarevich, et al. 2021. "Mycobacterium Leprae Diversity and Population Dynamics in Medieval Europe from Novel Ancient Genomes." *BMC Biology* 19 (1): 220.

"Picard." n.d. Accessed October 7, 2024. https://broadinstitute.github.io/picard/.

Pockrandt, Christopher, Aleksey V. Zimin, and Steven L. Salzberg. 2022. "Metagenomic Classification with KrakenUniq on Low-Memory Computers." *bioRxiv*. https://doi.org/10.1101/2022.06.01.494344.

Price, Alkes L., Nick J. Patterson, Robert M. Plenge, Michael E. Weinblatt, Nancy A. Shadick, and David Reich. 2006. "Principal Components Analysis Corrects for Stratification in Genome-Wide Association Studies." *Nature Genetics* 38 (8): 904–9.

Purcell, Shaun, Benjamin Neale, Kathe Todd-Brown, Lori Thomas, Manuel A. R. Ferreira, David Bender, Julian Maller, et al. 2007. "PLINK: A Tool Set for Whole-Genome Association and Population-Based Linkage Analyses." *American Journal of Human Genetics* 81 (3): 559–75.

Rampelli, Simone, Stephanie L. Schnorr, Clarissa Consolandi, Silvia Turroni, Marco Severgnini, Clelia Peano, Patrizia Brigidi, Alyssa N. Crittenden, Amanda G. Henry, and Marco Candela. 2015. "Metagenome Sequencing of the Hadza Hunter-Gatherer Gut Microbiota." *Current Biology: CB* 25 (13): 1682–93.

Renaud, Gabriel, Viviane Slon, Ana T. Duggan, and Janet Kelso. 2015. "Schmutzi: Estimation of Contamination and Endogenous Mitochondrial Consensus Calling for Ancient DNA." *Genome Biology* 16 (October):224.

Robb, John, Sarah A. Inskip, Craig Cessford, Jenna Dittmar, Toomas Kivisild, Piers D. Mitchell, Bram Mulder, et al. 2019. "Osteobiography: The History of the Body as Real Bottom-Line History." *Bioarchaeology International* 3 (1): 16–31.

Robinson JT, Thorvaldsdóttir H, Winckler W, Guttman M, Lander ES, Getz G, Mesirov JP. 2011. Integrative genomics viewer. *Nat. Biotechnol*. 29:24–26.

Rohart, Florian, Benoît Gautier, Amrit Singh, and Kim-Anh Lê Cao. 2017. "mixOmics: An R Package for 'Omics Feature Selection and Multiple Data Integration." *PLoS Computational Biology* 13 (11): e1005752.

Rousseeuw et al. 2017."'Finding Groups in Data': Cluster Analysis Extended [R Package Cluster Version 2.1.6]." 2023, December. https://cran.r-project.org/package=cluster.

Scheib, Christiana L., Ruoyun Hui, Eugenia D'Atanasio, Anthony Wilder Wohns, Sarah A. Inskip, Alice Rose, Craig Cessford, et al. 2019. "East Anglian Early Neolithic Monument Burial

Linked to Contemporary Megaliths." *Annals of Human Biology* 46 (2): 145–49.

Scheib, Christiana L., Ruoyun Hui, Alice K. Rose, Eugenia D'Atanasio, Sarah A. Inskip, Jenna Dittmar, Craig Cessford, et al. 2024. "Low Genetic Impact of the Roman Occupation of Britain in Rural Communities." *Molecular Biology and Evolution* 41 (9): msae168.

Scheib, Christiana L., Ruoyun Hui, Alice K. Rose, Anu Solnik, Eugenia D'Atanasio, Sarah A. Inskip, Craig Cessford, et al. 2023. "Local Population Structure in Cambridgeshire during the Roman Occupation." *bioRxiv*. https://doi.org/10.1101/2023.07.31.551265.

Schiffels, Stephan. n.d. *sequenceTools*. Github. Accessed July 24, 2024. https://github.com/stschiff/sequenceTools.

Schmieder, Robert, and Robert Edwards. 2011. "Quality Control and Preprocessing of Metagenomic Datasets." *Bioinformatics* 27 (6): 863–64.

Schönherr, Sebastian, Hansi Weissensteiner, Florian Kronenberg, and Lukas Forer. 2023. "Haplogrep 3 - an Interactive Haplogroup Classification and Analysis Platform." *Nucleic Acids Research* 51 (W1): W263–68.

Schubert, Mikkel, Aurelien Ginolhac, Stinus Lindgreen, John F. Thompson, Khaled A. S. Al-Rasheid, Eske Willerslev, Anders Krogh, and Ludovic Orlando. 2012. "Improving Ancient DNA Read Mapping against Modern Reference Genomes." *BMC Genomics* 13 (May):178.

Schubert, Mikkel, Stinus Lindgreen, and Ludovic Orlando. 2016. "AdapterRemoval v2: Rapid Adapter Trimming, Identification, and Read Merging." *BMC Research Notes* 9 (February):88.

Skoglund, Pontus, Bernd H. Northoff, Michael V. Shunkov, Anatoli P. Derevianko, Svante Pääbo, Johannes Krause, and Mattias Jakobsson. 2014. "Separating Endogenous Ancient DNA from Modern Day Contamination in a Siberian Neandertal." *Proceedings of the National Academy of Sciences of the United States of America* 111 (6): 2229–34.

Skoglund, Pontus, Jan Storå, Anders Götherström, and Mattias Jakobsson. 2013. "Accurate Sex Identification of Ancient Human Remains Using DNA Shotgun Sequencing." *Journal of Archaeological Science* 40 (12): 4477–82.

Stamatakis, Alexandros. 2014. "RAxML Version 8: A Tool for Phylogenetic Analysis and Post-Analysis of Large Phylogenies." *Bioinformatics* 30 (9): 1312–13.

Stewart, Robert D., Marc D. Auffret, Amanda Warr, Alan W. Walker, Rainer Roehe, and Mick Watson. 2019. "Compendium of 4,941 Rumen Metagenome-Assembled Genomes for Rumen Microbiome Biology and Enzyme Discovery." *Nature Biotechnology* 37 (8): 953–61.

Su, Manchun, Ziyun Hao, Huibin Shi, Taotao Li, Huihui Wang, Qiao Li, Yong Zhang, and Youji Ma. 2022. "Metagenomic Analysis Revealed Differences in Composition and Function Between Liquid-Associated and Solid-Associated Microorganisms of Sheep Rumen." *Frontiers in Microbiology* 13 (May):851567.

Taylor, C. C. 2002. "Nucleated Settlement: A View from the Frontier." *Landscape History: Journal of the Society for Landscape Studies* 24 (1): 53–71.

Tisza, Michael J., and Christopher B. Buck. 2021. "A Catalog of Tens of Thousands of Viruses from Human Metagenomes Reveals Hidden Associations with Chronic Diseases." *Proceedings of the National Academy of Sciences* 118 (23): e2023202118.

Velsko, Irina M., James A. Fellows Yates, Franziska Aron, Richard W. Hagan, Laurent A. F. Frantz, Louise Loe, Juan Bautista Rodriguez Martinez, et al. 2019. "Microbial Differences between Dental Plaque and Historic Dental Calculus Are Related to Oral Biofilm Maturation Stage." *Microbiome* 7 (1): 102.

Yu, G., Smith, D.K., Zhu, H., Guan, Y., and Lam, T.T.-Y. 2017. Ggtree: An r package for visualization and annotation of phylogenetic trees with their covariates and other associated data. Methods Ecol. Evol. 8:28–36.

Wang, Le-Heng, De-Quan Li, Yan Fu, Hai-Peng Wang, Jing-Fen Zhang, Zuo-Fei Yuan, Rui-Xiang Sun, Rong Zeng, Si-Min He, and Wen Gao. 2007. "pFind 2.0: A Software Package for Peptide and Protein Identification via Tandem Mass Spectrometry." *Rapid Communications in Mass Spectrometry: RCM* 21 (18): 2985–91.

Wood, Derrick E., Jennifer Lu, and Ben Langmead. 2019. "Improved Metagenomic Analysis with Kraken 2." *Genome Biology* 20 (1): 257.
